# Computational Study of the Human Dystrophin Repeats: Interaction Properties and Molecular Dynamics

**DOI:** 10.1371/journal.pone.0023819

**Published:** 2011-08-25

**Authors:** Baptiste Legrand, Emmanuel Giudice, Aurélie Nicolas, Olivier Delalande, Elisabeth Le Rumeur

**Affiliations:** 1 Université de Rennes 1, Rennes, France; 2 Equipe RMN-ILP, Faculté de médecine, UMR CNRS 6026, Rennes, France; 3 Equipe SDM, UMR CNRS 6026, Rennes, France; 4 Université Européenne de Bretagne, Rennes, France; Koç University, Turkey

## Abstract

Dystrophin is a large protein involved in the rare genetic disease Duchenne muscular dystrophy (DMD). It functions as a mechanical linker between the cytoskeleton and the sarcolemma, and is able to resist shear stresses during muscle activity. In all, 75% of the dystrophin molecule consists of a large central rod domain made up of 24 repeat units that share high structural homology with spectrin-like repeats. However, in the absence of any high-resolution structure of these repeats, the molecular basis of dystrophin central domain's functions has not yet been deciphered. In this context, we have performed a computational study of the whole dystrophin central rod domain based on the rational homology modeling of successive and overlapping tandem repeats and the analysis of their surface properties. Each tandem repeat has very specific surface properties that make it unique. However, the repeats share enough electrostatic-surface similarities to be grouped into four separate clusters. Molecular dynamics simulations of four representative tandem repeats reveal specific flexibility or bending properties depending on the repeat sequence. We thus suggest that the dystrophin central rod domain is constituted of seven biologically relevant sub-domains. Our results provide evidence for the role of the dystrophin central rod domain as a scaffold platform with a wide range of surface features and biophysical properties allowing it to interact with its various known partners such as proteins and membrane lipids. This new integrative view is strongly supported by the previous experimental works that investigated the isolated domains and the observed heterogeneity of the severity of dystrophin related pathologies, especially Becker muscular dystrophy.

## Introduction

The stability of muscle cells depends on the ability of cytoskeletal proteins to dynamically resist the mechanical shear stresses which occur during muscle activity. Dystrophin is one of these skeletal muscle cytoskeletal proteins [1]–[2] and is part of the large dystrophin-glycoprotein sarcolemmal complex [3]–[4]. Its complete genetic deficit in Duchenne muscular dystrophy (DMD) [5] leads to frequent sarcolemma ruptures followed by cell degeneration. Therefore, the current hypothesis is that dystrophin protects muscle cell membranes from rupture [6].

Dystrophin is a huge scaffolding protein of 427 kDa, made up of four major domains [4], [7]. The two N-terminal calponin homology sub-domains constitute an actin-binding domain (#1). After a first hinge is the large central rod domain (#2), composed of 24 spectrin-like repeats interrupted by two more hinges. This domain interacts with membrane phospholipids and with a number of cytosolic proteins such as filamentous actin (F-actin), n-nitric oxide synthase (nNOS) and microtubules. After a fourth hinge, there is the cysteine-rich domain (#3), which anchors dystrophin to the intrinsic membrane protein β-dystroglycan. Finally, the coiled-coil structured C-terminal domain (#4) interacts with the cytoplasmic proteins syntrophin and dystrobrevin. Through these numerous interactions, dystrophin covers the sub-sarcolemma surface with a dense network and may resist elongation during muscle contraction [4], [7].

The dystrophin central rod domain represents about 75% of the entire protein and this conserved structural domain makes it a member of the spectrin-like protein family, which also includes utrophin, spectrin and α-actinin [8]. The sequence similarity between members of this family is rather low, and their main common feature is the presence of numerous repeated sequences of approximately 100–110 residues called spectrin-like repeats. The structural basis of these repeats is the presence of heptad patterns, i.e., periodic patterns of seven hydrophobic and hydrophilic/charged residues usually denoted by the letters “a” through “g”. The residues in positions “a” and “d” are hydrophobic and ensure folding into triple alpha-helical anti-parallel coiled-coils [9]–[10]. The residues in the other positions are usually hydrophilic and/or charged. In spectrin and α-actinin, the contiguous repeats are connected by helical linkers that ensure continuity between the last helix of the first repeat and the first helix of the next repeat.

Although we and other groups have tried to solve the three-dimensional (3D) structures of different parts of the dystrophin central domain by X-ray crystallography and NMR, no atomic structures are yet available. At this time, the 3D structures of one isolated spectrin repeat [11], eight multi-repeat spectrin domains [12]–[19] and the α-actinin four-repeat domain [20]–[21] are the only structures that have been solved by X-ray crystallography. Only one 3D spectrin repeat structure has been solved by NMR [22]. The structural study of both spectrin and α-actinin may have been facilitated because they naturally exist as oligomers [8], [23]. In the crystals, spectrin and α-actinin repeats always appear as dimers, but dystrophin is not expected to. In consequence, the only structural information available for the dystrophin repeats has been obtained by circular dichroism and tryptophan fluorescence [24]–[29].

Because of the lack of experimental 3D structural data for dystrophin rod domain repeats, it is necessary to use comparative modeling and structural prediction to study their molecular properties. The utility of such approaches in designing experiments and interpreting experimental results is now widely recognized [30]–[31]. In this context, the goal of the present work is to highlight the structural differences among dystrophin repeats in complementarity of their known biochemical properties. Through the combination of homology modeling and the comparison of surface properties and molecular dynamics simulations, we have proposed a molecular description of the whole dystrophin central rod domain. We have shown that, despite their similar helical coiled-coil structures, dystrophin repeats display a huge diversity of surface electrostatics and hydrophobicity, as well as varying flexibility. The succession of repeats with specific properties and the presence of flexible junctions delineate seven independent structural regions, each of which may play a specific role in dystrophin activity. Our results provide evidence for the role of the dystrophin central rod domain as a scaffold platform interacting with various partners, such as proteins and membrane lipids, through a wide range of surface features and biophysical properties.

## Methods

### Sequence alignment

The sequence of human dystrophin was retrieved from NCBI Dp427m and is consistent with the cDNA sequence of the plasmid pTG11025 harboring the cDNA for the Dp427m muscle isoform of human dystrophin (National Center for Biotechnology Information Nucleotide Data Base NM-004006, provided by S. Braun Transgene, France) used in previous experimental work. Two sequence alignments of the dystrophin repeats are still commonly used, even though they differ somewhat from each other. The first appeared very early after the discovery of dystrophin [32], and the second includes utrophin sequences [33]. It appears that for 14 of the 24 repeats, the repeat starting point shifts by one residue between the two alignments. To optimize the first alignment, Koenig and Kunkel deleted a few residues and introduced some gaps [32]. In the alignment by Winder, there is no deletion. For this reason, we chose to use the alignment by Winder to define the boundaries of the different repeats [33], as shown in Figure S1.

### Secondary and 3D structure prediction

**Figure 1:**
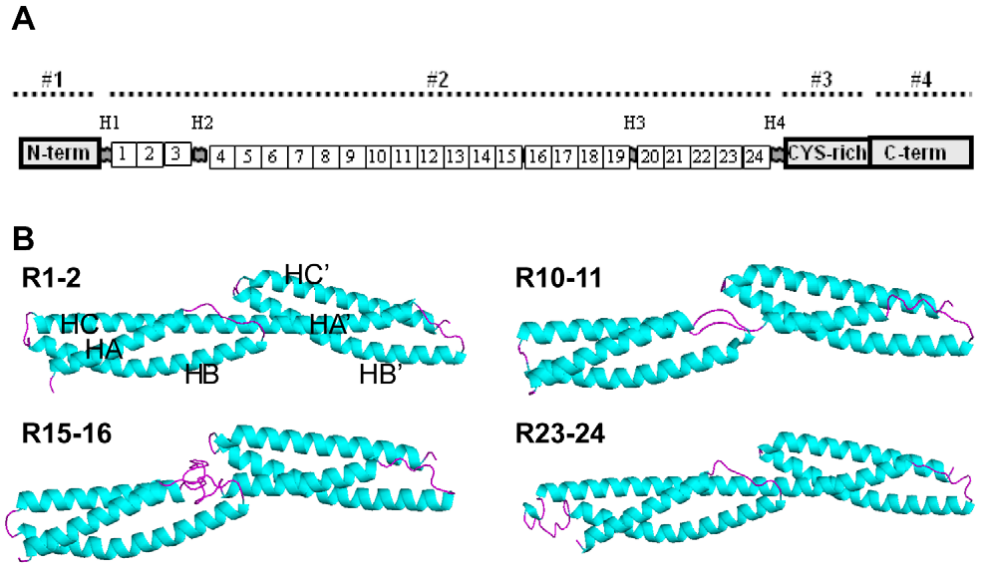
Dystrophin representation and 3D models of four representative tandem repeats. (A) Shown are the N-terminal (N-term), 24 spectrin-like repeats, cysteine-rich, C-terminal (C-term) domains and the four hinges (H1 to H4). The succession of the four main domains 1 to 4 is indicated above the diagram by a dotted line. (B) Four representative tandem-repeat models are shown as Cα backbone traces. Helical segments are colored in blue and loops are in pink. The N-terminal is on the left, the C-terminal is on the right, and the helix A of the N-terminal repeat of each tandem is at the front of the image. As a reminder, the nomenclature of the helices is given for the R1-2 model. HA, HB and HC are the helices of the first repeat, and HA', HB' and HC' are the helices of the second repeat of the tandem.

The secondary structure was predicted using PSIPRED [34]–[35]. For 3D structure prediction, I-TASSER combines various techniques such as threading, *ab initio* modeling and structure refinement approaches [36]–[37]. The sequences of two successive tandem repeats were submitted with an overlap of one repeat for the next submission, i.e., first the repeat 1–2, then the repeat 2–3, leading to a total of 21 models. The two tandem repeats that would include known hinges, R3-4 and R19-20, were omitted (Fig. 1A). This strategy was used to obtain models for the potential helical linkers between adjacent repeat pairs. I-TASSER produced one to five models for each of the two-repeat sequences submitted, and only the model with the best C-score for each tandem repeat was retained. These representative structures were analyzed on a graphical display with PyMOL [38] and their quality assessed using PROCHECK, ProSA-web [39]–[40] and Verify3D [41]–[42].

### Surface-property comparisons: hydrophobicity

Surface hydrophobicity was obtained using PLATINUM, which is designed to calculate match or mismatch in receptor-ligand complexes [43]–[44]. This program allows the calculation and visualization of the molecular hydrophobic/hydrophilic surface properties using the concept of molecular hydrophobicity potential (MHP). The PLATINUM algorithm also provides the total hydrophobic and hydrophilic surfaces for a molecule. All the models were submitted, and the PLATINUM web site provided the calculated maps, which were then visualized using PyMOL.

### Surface-property comparisons: electrostatics

The web-based version of PIPSA (Protein Interaction Property Similarity Analysis) [45]–[48] was used to compare the tandem repeats with respect to their electrostatic potentials. PIPSA quantifies the similarity in the surface properties of homologous proteins and is particularly useful for comparing the surface properties of the dystrophin tandem repeats. The previously fitted models were submitted to the PIPSA server [48], and we chose the Adaptive Poisson-Boltzmann Solver (APBS) software to calculate the electrostatic potentials [49]. A low concentration (<10 mM) of monovalent ion tends to accentuate differences between the electrostatic patches, while at cellular concentration (150 mM), the contrast between the different parts of the electrostatic surface is strongly attenuated. For this reason, we chose to present maps calculated for an intermediate ionic strength of 50 mM. Similarity indexes (SI) for each pair of surface electrostatic potentials were calculated [45] and converted into a distance matrix ranging from 0.5 to 1.5 [46]–[47]. The distance matrix was subsequently subjected to a clustering procedure, and the corresponding dendrogram was transformed into an electrostatic similarity tree using the neighboring-joining algorithm by PHYLIP [50]. Visualization of the electrostatic potentials on the molecular surfaces of the repeats was produced using PyMOL and the APBS algorithm for consistency.

### Molecular dynamics relaxation

To simulate our systems of tandem repeats, water and ions, we used the program NAMD 2.7b2 [51] and the CHARMM27 force field [52]–[55]. The initial models of the dystrophin tandem repeats were oriented along the z axis and then solvated in rectangular water boxes generated using the *Solvate* plugin of VMD [56]. We thus ensured that there was a 30 Å thick layer of TIP3P water in the x and y directions and a 15 Å thick layer in the z direction. Subsequently, the VMD plug-in *Autoionize* was used to place ions randomly to neutralize the system while maintaining a 150 mM NaCl concentration. To adjust the position of the solvent (water and ions) around the molecules, each system was energy minimized for 10000 steps using the conjugate gradient method while restraining the solute atoms with a 25 kcal mol^−1^ Å^−2^ harmonic restraint. The box size was chosen to be big enough to prevent any bias of the Periodic Boundary Conditions on the simulations. The simulated system dimensions are given in Table S2.

The entire system (solvent + solute) was then subjected to another 10000 steps of energy minimization to relieve any major stresses, followed by a slow heating to 310 K at constant volume over a period of 50 ps. The production phase was performed for 31 ns under periodic boundary conditions with a 2 fs time step using the SHAKE algorithm. Van der Waals interactions were computed using a cut-off distance of 12 Å with a switching function starting at 10 Å, while long-range electrostatic forces were calculated using the particle–mesh Ewald method with a grid density of 1 Å**^−^** ^3^. To further reduce the cost of computing full electrostatics, a multiple-time-stepping procedure was employed to calculate long-range electrostatics every 4 fs. Berendsen baths were used to maintain the system temperature and pressure at 310K and 1 atm, respectively.

The post-processing analysis of the MD trajectories was performed with VMD 1.8.7 [56] and Ptraj using the last 20 ns of simulation. To extract representative structures, the coordinate frames from the trajectory were clustered using the K-means algorithm. After testing different values, we chose to split the trajectory into two clusters using the pairwise RMSD between frames as a metric to compare the Cα atoms of the protein [57]. The Atomic coordinates of the snapshots closest to the center of the resulting clusters (C1 and C2) are available as pdb files in MD-Clusters S1. The results were verified by repeating the simulation for 20 ns using an identical protocol, the same initial model but a different initial velocity distribution.

## Results

### Sequence analysis and secondary structure prediction

Despite a low overall similarity, the sequence alignment showed that most of the residues in the (a) and (d) positions were well conserved, while most of the residues in the other positions were not (Fig. S1A). In all but seven cases, the heptad pattern was maintained through the linker (Fig. S1B). In the R4-5 and R10-11 linkers, one residue was missing and in the R1-2 and R13-14 linkers, two residues were missing. In contrast, insertions of 2, 20 and 7 residues were present in R5-6, R15-16 and R18-19, respectively (Fig. S1B). 79 to 91% of the total structure was predicted to form α-helices by PSIPRED (not shown). This result was in agreement with the assumption that the dystrophin repeats are essentially folded in a triple α-helical coiled-coil, and also with experimental data obtained on *in vitro* produced repeats of dystrophin [27]–[29]. It is also worth noting that a decreased tendency to form a helix was predicted for the center of helix B of each repeat.

### Structure models of tandem spectrin-like repeats of dystrophin

Not surprisingly, the I-TASSER threading procedure identified spectrin repeats as the best templates, specifically 1U4Q (chicken-brain α-spectrin repeat R15-17) [14], 1S35 (erythroid β-spectrin R8-9) [13] and 3EDV (β2-spectrin repeat R14-16) [15]. As expected for spectrin-like repeats, the identity score was low, ranging between 0.08 and 0.18. However, the sequence coverage was very good, with values ranging between 86 and 99%, and the C-scores ranged between -0.72 and 0.76, which indicated that all models had correct folds (Table S1). The models were further assessed with VERIFY3D [42], ProSA [39]–[40] and PROCHECK [58], with results indicative of high-quality models (Annex S1). All the models are available as pdb files in Models S1.

The models of the tandem repeats all appeared as elongated triple helical coiled-coils of roughly 100×50 Å dimensions (Fig. 1B, Fig. S2), which is in accord with the structural templates. Each repeat consisted of three helices and two loops. The helices were not straight but curved gently to form a left-handed super coil. Compared to the available spectrin-like repeat structures, the canonical kink in the center of helix B of each repeat was observed in all repeats, except for R9 and R14 in the R9-R10 and the R14-15 tandem models, respectively.

A long helical linker between the two consecutive repeats was found in 17 of the 21 tandem models. For R5-6, R10-11, R13-14 and R15-16, the linker was not helical but was a short loop (R5-6, R10-11), a long loop (R15-16) or a break (R13-14). In R5-6, R10-11 and R13-14, the absence of the helical linker may have been due to the presence of one or two proline residues which might impair the helical folding of the linker. In R15-16, a 20-residue insertion between the two repeats has been previously suggested to form a small loop [59], as it appears in our model.

The Cα-atoms RMSD of our models ranged from 0.250 to 4.241 Å. These values were comparable to those calculated between the various spectrin repeat structures, which ranged from 0.761 to 4.413 Å for the template structures (1U4Q, 1S35 and 3EDV) used in our modeling (Fig. S3).

### Molecular descriptors

**Figure 2:**
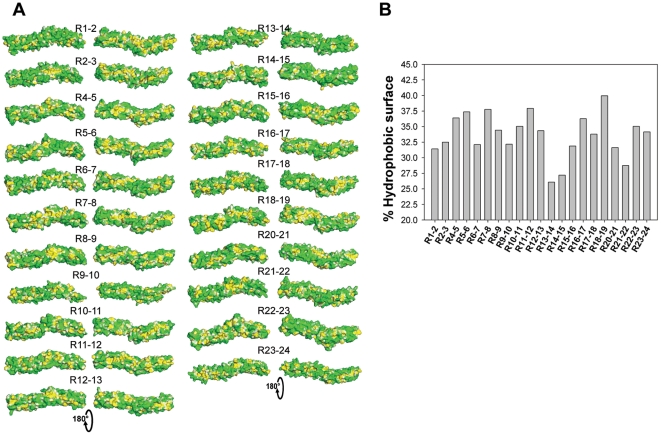
Molecular hydrophobicity potential of the dystrophin tandem-repeat models. Molecular hydrophobicity potential surfaces were obtained with PLATINUM and displayed using PyMOL. (A) As in Figure 1B, for the image on the left of each model, the molecules are presented with the helix A in front, the N-terminal end on the left and the C-terminal on the right. The right-hand image of each model is rotated 180° along the molecule's long axis, as indicated on the bottom. The hydrophobicity scale is green-white-yellow, with green representing the most hydrophilic regions and yellow the most hydrophobic. (B) Plot of the calculated % of hydrophobic surface of each tandem repeat by PLATINUM.

As is often the case with cytoplasmic proteins, the overall tandem repeat surfaces were mostly hydrophilic with an average of 66.4 ± 3.5% hydrophilic surfaces (Fig. 2A, B). R13-14 and R14-15 constituted a central region that was highly hydrophilic compared to other regions (by less than one standard deviation from the mean), while R18-19 was highly hydrophobic (more than one standard deviation from the mean) (Fig 2B). We were also able to detect numerous hydrophobic patches dispersed on the surface and small hydrophobic grooves (R2-3, R5-6 and R10-11) that might constitute interaction sites.

**Figure 3:**
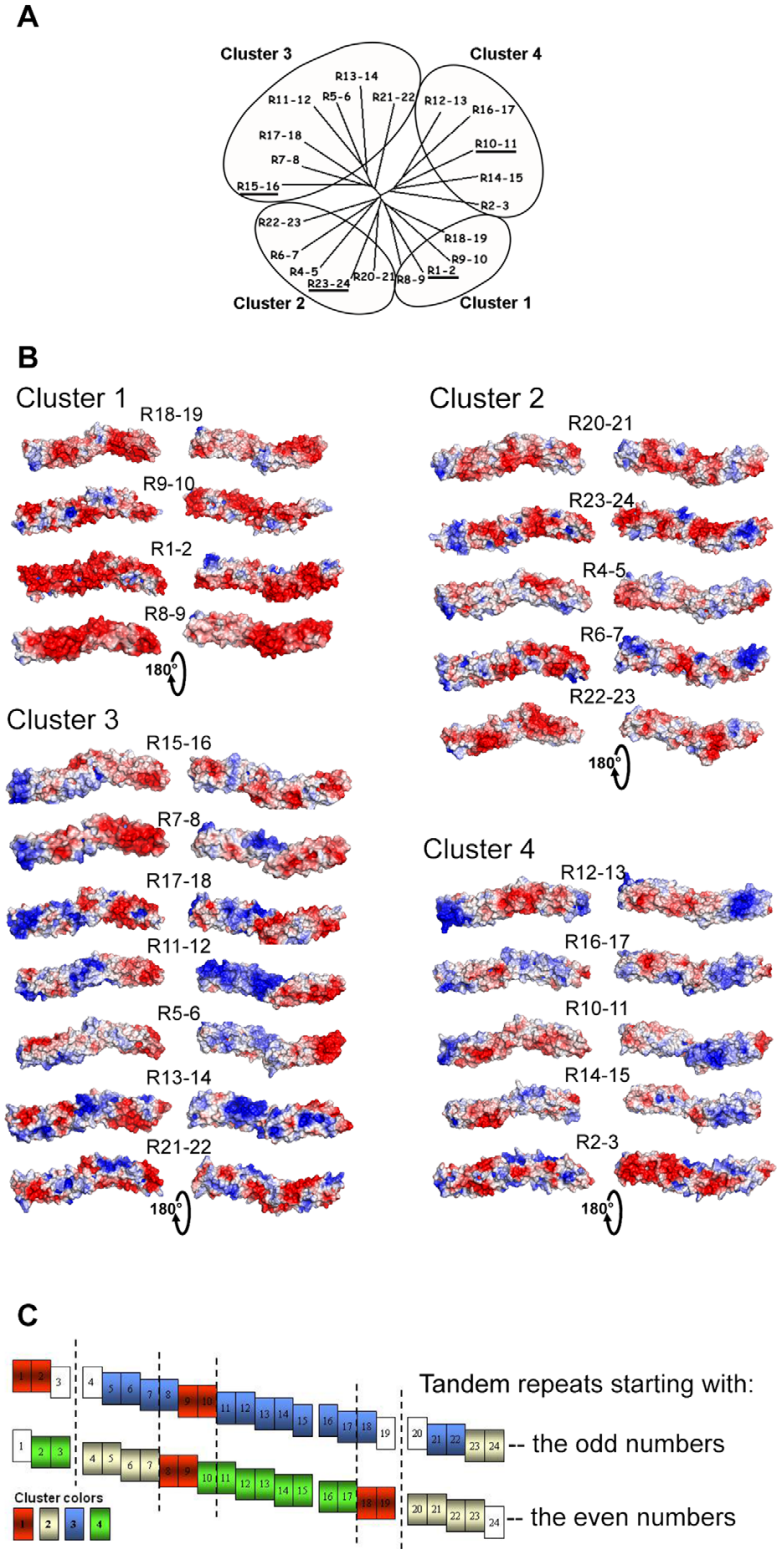
Molecular electrostatic potential surfaces and PIPSA clustering of the dystrophin tandem-repeat models. (A) The dendogram from the PIPSA analysis of the 21 dystrophin tandem repeats showed four clusters. The 4 tandem repeats used in the molecular dynamics simulation are underlined. (B) Representation of the electrostatic potential projected on the solvent accessible surface of the dystrophin repeats, separated into these 4 clusters. Each model was colored using the APBS electrostatic potential calculated for an ionic strength of 50 mM, and the surface colors were clamped at -3 (red) and +3 (blue) kTe^-1^. The molecules are presented with the same orientation as in Fig. 2A. (C) Graphical representation of the repartition of the electrostatic clusters along the rod domain. To take into account the overlapping repeats, the tandems are shown here in two lines, one for tandems starting with an odd number (R1-2, R5-6, etc.), and the other for the evens (R2-3 etc). Each tandem repeat is colored to indicate in which cluster it belongs: red for cluster 1, grey for 2, blue for 3 and green for 4. The uncolored repeats belong to tandem not studied because of the presence of a hinge. Dotted vertical lines are drawn to emphasize the presence of six specific regions.

The electrostatic surfaces of the tandem repeats were clearly dissimilar, and they showed large positive and negative potential patches (Fig. 3). Such patches often appeared to extend over more than one repeat surface (R5-6 for example), while sometimes the two repeats in a tandem exhibited opposite electrostatic properties (R7-8 for example). To further quantify the surface electrostatic potential similarity of the tandem repeats, we analyzed our models with PIPSA [48]. The resulting dendrogram is divided into four clusters (Fig. 3A). Globally, the ratio of negative/positive potential surfaces decreased from cluster 1 to cluster 4 (Fig. 3B). In cluster 1, the models exhibited large strongly negative patches extending over all tandem repeats. This underlined three strongly negative regions in the dystrophin rod domain, constituted by R1-2, R8-10 and R18-19. The tandem repeat surfaces in cluster 2 had small charged patches with numerous negative and few positive moieties. Cluster 3 was constituted by tandem repeats with large and strongly positive patches in the N-terminal repeat of the tandem and negative patches in the C-terminal end. The electrostatic surfaces in the cluster 4 were comparable to those in cluster 3 but with the large positive moieties rather in the C-terminal repeat of the tandem. The analysis clearly indicated surface-property alternations, particularly in the R10-18 part of the rod domain, i.e., R11-12, R13-14, R15-16, R17-18 are in cluster 3 and R10-11, R12-13, R14-15 are in cluster 4 (Fig. 3C). This region was previously considered as a highly basic region in view of the calculated pI of the single repeats [60], but it appears that the region is in fact made up of alternating repeats with opposite electrostatic properties.

### Molecular dynamics relaxation

To further assess the quality of the proposed models, we studied tandem repeats by molecular dynamics. We applied this approach to four selected models bearing different types of linker and physical properties. Two of the chosen repeats displayed helical linkers (R1-2 and R23-24), and two displayed non-helical linkers (R10-11 and R15-16). R1-2 belonged to the R1-3 domain, which binds to lipids, while R23-24 belonged to the R20-24 domain, which does not [27]. The thermal stability of R1-2 was ten degrees lower than that of R23-24 [29]. In R10-11, the linker consisted of a short loop, while there was a long unstructured linker of 20 residues in the R15-16 model. The four tandem repeats were also chosen to cover the whole range of electrostatic properties, one from each of the four clusters in the Fig. 3 dendrogram.

**Figure 4:**
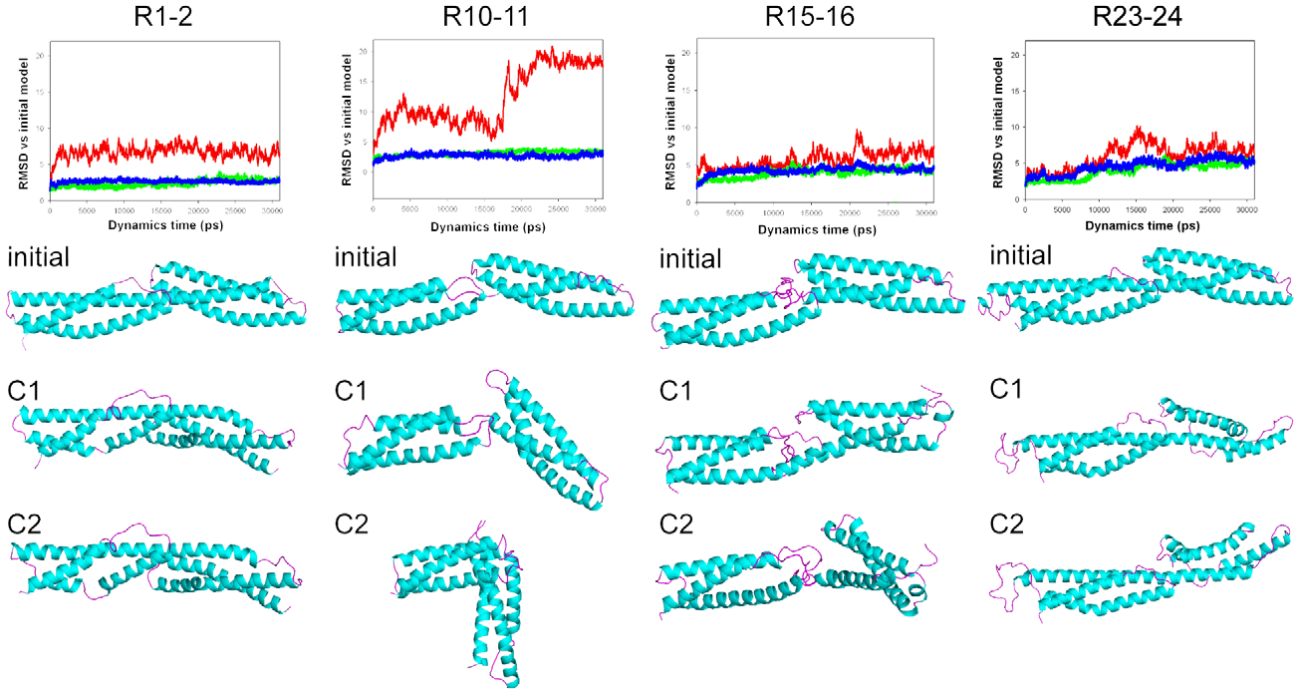
Structural changes in the four tandem repeats observed during the molecular dynamics relaxation. Cα RMSD measured between the initial models and snapshots of the four molecules taken every ps. The red line represents the RMSD of the tandem repeats, the green indicates the RMSD of the N-terminal repeat in the tandem and the blue shows the RMSD of the C-terminal repeat. The simulations were submitted to a clustering procedure in order to identify two clusters per simulation. Cα backbone traces of the initial model and the snapshots closest to the center of each cluster (C1 and C2) are shown with the same orientation as in Fig. 1B.

As shown by the RMSD of snapshots measured along the whole trajectory, the three simulations for R1-2, R15-16 and R23-24 converged after 10 ns, while the R10-11 simulation clearly sampled two different conformations (Fig. 4). As expected, the RMSF (Fig. S4) showed that the more flexible regions corresponded to the loops between the helices. All helices remained stable with the exception of R24 HB. It is also worth noting that the heptad pattern (black arrows, Fig. S4) was well maintained, with a lower RMSF for the hydrophobic residues in positions (a) and (d).

The R1-2 tandem was relatively stable throughout the simulation (Fig. 4), with a well-maintained internal structure for both repeats and for the helical linker. The two clusters obtained from the trajectory were very similar. However, the structure diverged rapidly from the initial model. Both R1 HB and R2 HB bent and the two helices rotated around the helical linker to finally interact with each other. At the same time, there was a slight unfolding of R1 HB around residues H55 to G57. The rotation had only a limited effect on the percentage of accessible hydrophobic surface vs. the total molecular surface (Fig. S5 A, B). R1-2 is the most negatively charged tandem repeat of the dystrophin rod domain (Fig. 3), and the conformational rearrangement only slightly modified the electrostatic surface of the molecule (Fig. S5B). Interestingly, the slight unfolding and the bending of R1 HB clearly individualized the putative amphipathic lipid-packing sensor (ALPS) motif of the repeat, i.e., residues Q56 – G73 [61].

Starting from an elongated shape, R10-11 began to bend at the non-helical linker within the first nanosecond. The conformation was then stable for several nanoseconds until the molecule was reorganized into an ever more kinked structure (Fig. 4). Nevertheless, both repeats remained very stable through the trajectory and only their relative positions changed. The same results were observed in the control trajectory. This profound rearrangement was likely driven by the presence of numerous exposed hydrophobic residues at the linker, as pointed out by the significant decrease of the hydrophobic contribution to the molecular surface (Fig. S5A). To minimize the hydrophobic cost, the beginning of R10 HB first interacted with the center of the small R11 HA. The conformation was further stabilized through the formation of multiple contacts between R10 HB, R10 HC and R11 HB, and a tight fit between the two repeats. This structural rearrangement strongly modified the electrostatic properties of the molecule, leading to the appearance of a substantial positively charged pocket with negatively charged surfaces on both sides (Fig. S5B).

The conformation of R15-16 was stable (Fig. 4), although the total RMSD increased slightly along the trajectory due to the flexibility of the long non-helical linker. Interestingly, the cluster analysis identified two preferred conformations that interchanged during the trajectory. The main difference between the two clusters was due to variation in the distance between the non-helical linker and the R16 HB-HC coiled-coil. The interaction between R15 HB and R16 HA was maintained throughout the simulation to minimize the exposure of hydrophobic residues. Nevertheless, the hydrophobic and electrostatic surface properties were mostly the same for the two clusters and differed only slightly from the initial model (Fig. S5A, B).

After a first rearrangement, the R23-24 trajectory converged, although the global conformation remained quite flexible for the rest of the simulation (Fig. 4). There was a great flexibility of the internal coil between R23 HB and HC, but the global conformation of this repeat did not change. However, there was a substantial rearrangement of R24 HB, with an unfolding of its C-terminal extremity. In contrast to R1-2, the relative orientation of the two repeats was maintained, and no interaction between the HB helices was observed. Accordingly, the hydrophobic and the electrostatic surface properties were only slightly modified (Fig. S5A, B).

## Discussion

The existing assumption in the field is that dystrophin is a key mechanical linker in the muscle fiber through its association with both the cytoskeletal protein F-actin and the plasma membrane-intrinsic protein β-dystroglycan [4], [62]-[63]. The central rod domain itself has been considered to constitute a passive linker, the role of which is to absorb the mechanical tension created by muscle contraction [6]. However, in contrast to this simple assumption, an increasing number of interacting partners of the central rod domain are being discovered, which suggests a more complex biological role.

### The models and the nature of the inter-repeat linkers

We have shown that reasonable atomic models can be obtained using I-TASSER, as validated by various assessment procedures. The secondary structures fit well with the spectrin sequences alignment analysis and with the prediction from PSIPRED. In addition, the coiled-coil spectrin-like fold is in agreement with the helicity yields of previously published circular dichroism measurements [24]–[29]. The low helicity values sometimes observed experimentally were likely due to the absence of neighboring domains. Furthermore, the surface hydrophobicity indicates that the hydrophobic “a” and “d” residue side chains stabilizing the coiled-coil are largely buried inside the models. As a consequence, we observed that the repeat structures remained mostly stable during the molecular dynamics relaxations.

According to the hypothesis of similar folds for dystrophin, spectrin and α-actinin repeats, all the models fit well with the canonical triple-helical coiled-coil structure obtained by X-ray crystallography and NMR of spectrin repeats [11]–[21]. However, in contrast to the multi-repeat spectrin structures in which all the linkers were helical, we observed that four tandem-repeat models displayed non-helical linkers (R5-6, R10-11, R13-14 and R15-16). In R15-16, the presence of a 20-residue additional sequence prevented the linker from being helical. The absence of a helical fold for the other three linkers may be due to disruptions of the heptad pattern and/or to the presence of proline residues. However, in two other tandem repeats, R1-2 and R4-5, the heptad pattern rupture did not impair the linker's helical fold. Therefore, we concluded that the presence of proline residues in R5-6, R10-11 and R13-14 was likely to be the main cause of the non-helical linkers. Interestingly, no proline residues are located in the inter-repeat linkers of α- and β-spectrin and α-actinin. However, these proline residues are conserved in the dystrophin from other vertebrates, such as mice and dogs, and are also present in the linkers of human utrophin repeats 5-6, 10-11, 13-14, 14-15 and 16-17. Thus, the presence of proline residues in the inter-repeats linker constitutes a key feature of dystrophin and utrophin molecules.

### Surface properties of the tandem repeats

The varied interaction properties of the dystrophin rod domain must be supported by varied surface properties among the repeats. We show here that the repeat surfaces are mostly hydrophilic, in accordance with the heptad pattern's projection of the polar residue side chains outside the coiled-coil. However, the surface hydrophobicity is far from zero, and the repeat surfaces also displayed hydrophobic patches of potential interest.

**Figure 5:**
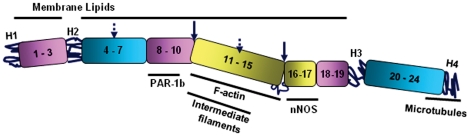
Novel view of the dystrophin central rod domain sub-divided into seven specific structural domains. H1 to H4 are the four hinges 1 to 4. The new sub-domains are shown here in boxes, with the repeats that they enclose indicated. The colors of the boxes are derived from the electrostatic analysis of Fig. 3B, C. Violet boxes are the most electronegative regions and are mostly constituted by repeats from cluster 1. The blue boxes are intermediate electrostatic regions composed of repeats from clusters 2 and 3. The yellow boxes correspond to a region previously considered as highly basic but which is in fact made up of alternating repeats from clusters 3 and 4 with opposite electrostatic properties. The two solid arrows indicate the newly identified small junctions, while the two dashed arrows indicate the locations of two other putative small junctions between R5 and R6 and R13 and R14. The black straight lines indicate partners and are placed along the dystrophin regions with which they interact. They include nNOS (n-nitric oxide synthase), F-actin (filamentous actin), PAR-1b (polarity-regulating kinase-1b), membrane lipids, intermediate filaments and microtubules.

The use of APBS and PIPSA allowed us to calculate and compare the electrostatic surface potentials of tandem or single repeats of the entire rod domain. Considering all the models, despite substantial heterogeneity of the electrostatic surfaces, the analyses highlighted six distinct regions based on their electrostatic potentials. R1-3, R8-10 and R18-19 are strongly negatively charged regions; R4-7 and R20-24 are less charged with small negative and positive patches; and the R11-17 region is composed of large, alternating strongly negative and positive moieties (Fig. 3B and Fig. 5).

The remaining question is as follows: does this variety of surface properties explain the localization of specific binding sites for the known partners of the dystrophin rod domain? Indeed, the two strongly negatively charged regions R1-3 and R18-19 strangely present strong anionic lipid-binding properties, while R10-17, with its large negatively and positively charged patches, shows a strong affinity to both anionic and zwitterionic lipids [28]. In contrast, the two less charged regions can either bind to lipids, as in the case of R4-7, or not, as for R20-24 [27].

Although the description of lipid binding is quite complex, our models provide some clues as to how the lipids bind to specific repeats. Molecular dynamics experiments with membranes should further define where and how the complementarities lie. On the other hand, surface-property descriptors are clearly insufficient to explain the interaction specificity between the dystrophin repeats and their protein partners. This can be explained by the crucial role played by both surface and residue complementarities in these processes. To answer this question, we plan to dock our models on atomic structures of well-known dystrophin partners such as nNOS [64] and filamentous actin [65].

### Molecular dynamics relaxation of tandem repeats

Although our models are of high quality, there may be a bias because of the small number of spectrin-repeat templates available. It is therefore of interest to study the molecular dynamics of the dystrophin repeats. Interestingly, three of the four studied models maintained structures very similar to the initial models throughout the entire simulation. The exception was R10-11, where the presence of both a flexible non-helical linker and very strong hydrophobic forces induced a large kink and important changes in the global structure of the tandem, while each of the repeats remained close to the initial model. In contrast, the long linker of R15-16 allows only a restricted bending of the two repeats with respect to each other, and the R1-2 and R23-24 models preserved their helical linkers. These results show the potential limitations of homology modeling and underline the importance of refining structures by MD.

Our observations should also be compared to similar simulation approaches on spectrin repeats. Dynamic flexibility has been previously reported for repeats 8-9 of human erythrocyte β-spectrin and repeats 16–17 of chicken-brain α spectrin, both with helical linkers [66]. As in our work, the internal structure of each repeat was not modified during the simulations. Atomistic molecular dynamics showed significant bending flexibility governed by the interactions among the AB-loop of the first repeat, the BC-loop of the second repeat and the linker region. At the end of the simulation, the two repeats were in the same face of the linker. This result is in agreement with the observed changes in the respective orientation of the repeats in R1-2, which move onto the same face of the linker early in the simulation. In contrast, R22-23 remained in the same orientation as in the starting models. Therefore, it appears that in dystrophin repeats, the bending directionality is not correlated to the structure of the linker.

Interestingly, these dynamical properties can be directly linked to the presence of specific binding domains. In the case of R1-2, which is known to bind to anionic and curved liposomes, the rapid rotation of repeat 2 around the helical linker placed the potential amphipathic lipid-packing sensor (ALPS) motif [61], situated at the end of HB of R1, on the outside of the molecule. This mechanism suggests a dynamical control of the interaction with curved membrane surfaces. In the case of R10-11, the large observed kink can be compared with the large differences in the adjacent-repeat orientation observed for βI-spectrin [16]–[17] and β2-spectrin [15]. In both studies, repeat 15 exhibits a large tilt angle with respect to repeat 14. Significantly, this tandem repeat constitutes the ankyrin-binding domain, and mutations that interrupt the bonding between two residues essential for maintaining the large tilt angle have been shown to decrease the ankyrin affinity of the tandem repeat [17]. The binding surface of the tandem repeat 14-15 and its electrostatic complementarities [19], [67] are disrupted when the angle is no longer present, and this change in turn alters the ankyrin binding. These observations lead to a very interesting hypothesis [17], [19] in which modifications of the angle may be dynamically controlled either by the binding of ligands such as lipids [68] or by a mechanical stretch, thus regulating the ankyrin binding. Similarly, the N-terminal end of the αI and αII-spectrins forms a large tilt angle with the neighboring repeat 1, consistent with a flexible junction [69]. Again, this flexible junction is thought to play a role in modulating the association affinity of spectrin α with spectrin β to constitute the spectrin dimer. Our results suggest a similar mechanism for R10-11 that could dynamically control the binding of the neighboring region R11-17 to F-actin.

Altogether, our simulations provided evidence of at least two highly flexible linkers that constitute small “junctions” allowing the individualization of the R11-15 region from its neighbors. Strikingly, this region coincides with the initial description of the central actin-binding domain (ABD2) of dystrophin [60], which was later extended to R11-17 [70]. In the absence of an actual molecular mapping of the actin-interacting domain, it is tempting to consider the possibility that the R11-15 domain is the true ABD2. Furthermore, the remaining R16-17 region has been recently shown to play a specific role through interactions with nNOS. Therefore, the dynamics of the two non-helical linkers R10-11 and R15-16 may regulate the interaction of the two close regions R11-15 and R16-17 with their partners, actin filaments, nNOS and membrane lipids. The presence of non-helical linkers in the tandem repeats R5-6 and R13-14 could constitute two other small “junctions”, but this remains to be determined.

In contrast, the presence of a helical linker in the R20-24 region must rigidify this entire region. Indeed, the region is known to be the most thermally stable portion of the molecule and also shows specific surface properties. This stability and the presence of the following small hinge are likely crucial to properly individualizing the neighboring cysteine-rich domain, which interacts with the large DGC macromolecular complex through the integral β-dystroglycan protein [4].

### Conclusion

In conclusion, our computational analysis clearly establishes that dystrophin repeats are highly diverse, with electrostatic and hydrophobic surfaces that are far from identical. Our study also identifies new flexible junctions in the rod domain in addition to the already known hinges. Altogether, the dystrophin rod domain is made up of seven different regions distinguished by their surface properties and the presence of key flexible linkers on both sides (Fig. 5). Through the diversity of these properties, the repeats constitute a large scaffold domain for interactions with multiple proteins and with different lipid partners.

This improved description of the dystrophin central rod domain strongly supports the severity grading of Becker muscular dystrophy (BMD) [5], [71]–[73]. Indeed, beside the very severe DMD due to the total deficit of dystrophin, BMD varies from very mild to severe and with or without cardiac involvement in addition to the muscular damage. BMD is mainly caused by in-frame mutations which in the vast majority of cases are deletions of one or several exons. Apart for deletions of the 3′ and 5′ of the gene, the mutated dystrophin molecules are internally truncated by large parts of the central rod domain. However, the molecular basis of the variability of the BMD severity is difficult to establish because the precise properties of the central rod domain are yet largely unknown. Therefore, our work gives now an integrative view of the central rod domain properties which will help to interpret the BMD variability in view of the missing regions in truncated dystrophin molecules. Similarly, our work may help in the design of truncated dystrophin molecules to be expressed, either by gene replacement or by exon skipping, in Duchenne muscular dystrophy patients who lack dystrophin [63], [74].

We emphasized above that crystallization of dystrophin repeats has never been successful. In view of the biophysical properties of the different tandem repeats that we reveal here, key experiments will include crystallization assays on single or tandem repeats and SAXS studies [75] on multi-repeat proteins such as those previously studied by our group [27]–[28]. Our models also constitute a rational molecular platform for initiating docking studies with atomic structures of known partners such as nNOS, F-actin and lipids, and to guide site-directed mutagenesis to more precisely and experimentally define the surfaces involved in these interactions.

## Supporting Information

Figure S1 **Sequence alignments.** (A) Alignment of the 24 dystrophin repeats and the 8 spectrin repeats used by I-TASSER as templates. Repeats were aligned by ClustalW using default parameters. The alignment was visualized in Jalview and colored using the ClustalX color scheme. Each residue is marked by a specific color only when there is similarity across the repeats. In the bottom line, heptad motifs are indicated, showing the hydrophobic residues in the (a) and (d) positions. The presence of hinges or extra-sequences is mentioned at the end of the corresponding line. (B) The end of a repeat is aligned with the beginning of the following repeat to help visualize the linker within the tandem repeats. The heptad pattern is indicated as in (A). At the right, we indicate whether the heptad pattern is respected (+) or not (−) in the linker. Insertions are indicated by marking the hinges or the extra-sequences.
(TIF)

Figure S2 **Three dimensional homology models of the 21 dystrophin tandem repeats obtained by I-TASSER.** The models are represented as Cα backbone traces. Helical segments are colored in blue and loops are in pink. The N-terminal is on the left, the C-terminal is on the right, and the helix A of the N-terminal repeat of each tandem is at the front of the image. As a reminder, the nomenclature of the helices is given for the R1-2 model. A, B and C are the helices of the first repeat, and A’, B’ and C’ are the helices of the second repeat of the tandem.
(TIF)

Figure S3 **Superposition of the spectrin-repeat crystallographic structures and of the dystrophin tandem-repeat models.** Top: Spectrin repeats shown were those used as templates by I-TASSER: chicken-brain a-spectrin repeats R15, R16 and R17 (1U4Q) [14], erythroid β-spectrin repeats R8 and R9 (1S35) [13] and β2-spectrin repeats R14, R15 and R16 (3EDV) [15]. Bottom: superposition of the dystrophin tandem repeats modeled with I-TASSER. The figure was made using PyMOL.
(TIF)

Figure S4 **Quality assessment of the molecular dynamics relaxation of four tandem repeats.** The residue-by-residue backbone fluctuation profile (RMSF) of the eight repeat units R1, R2, R10, R11, R15, R16, R23 and R24 is shown with the primary sequence of each isolated repeat aligned according to the heptad pattern. The (a) and (d) residues are marked with black triangles.
(TIF)

Figure S5 **Influence of the molecular dynamics relaxation on the hydrophobicity and electrostatics of the molecule surfaces.** As in Figure 1B, for the image on the left of each model, the molecules are presented with the helix A in front, the N-terminal end on the left and the C-terminal on the right. The right-hand image of each model is rotated 180° along the molecule's long axis, as indicated on the bottom. The initial model and the snapshots closest to the center of each cluster (C1 and C2) are shown in both cases. (A) Molecular hydrophobicity potential surfaces calculated with PLATINUM are shown using PyMOL. The hydrophobicity scale is green-white-yellow, with green representing the most hydrophilic regions and yellow the most hydrophobic. (B) Representation of the electrostatic potential projected on the solvent accessible surface of the dystrophin tandem repeats. Each model was colored using the APBS electrostatic potential calculated for an ionic strength of 50 mM, and the surface colors were clamped at -3 (red) and +3 (blue) kTe-1.
(TIF)

Table S1 **I-TASSER statistics for the tandem-repeat models of the dystrophin central rod domain.**
(TIF)

Table S2 **Simulated system dimensions for the molecular dynamics study of the four tandem-repeats, R1-2, R10-11, R15-16 and R23-24.**
(TIF)

Annex S1 **Quality assessment of homology modeling of all tandem repeats of the human dystrophin rod domain.** For each model, the sequence and an image of the model as it appeared in Fig. 1 are shown first and second, respectively. Shown next are the results from PROCHECK, with the Z score and the graph of the energy for each residue in the sequence. The Verify3D results follow, along with the Ramachandran plot from PROCHECK. This is completed by an image of the model, with the residues in the disallowed regions of the Ramachandran plot colored in red.
(PDF)

Models S1 Atomic coordinates (in PDB format) of 3D homology models of the 21 dystrophin tandem repeats obtained with I-TASSER.
(RAR)

MD-Clusters S1 Atomic coordinates (in PDB format) of the snapshots closest to the center of each cluster (C1 and C2) calculated for the molecular dynamics trajectories of the tandem repeats R1-2, R10-11, R15-16 and R23-24.
(RAR)
