# Supplementary figures and images for: Computational Study of the Human Dystrophin Repeats: Interaction Properties and Molecular Dynamics
Source: PLoS One. 2011 Aug 25;6(8):e23819. doi: 10.1371/journal.pone.0023819 (PMC3162007; doi:10.1371/journal.pone.0023819)

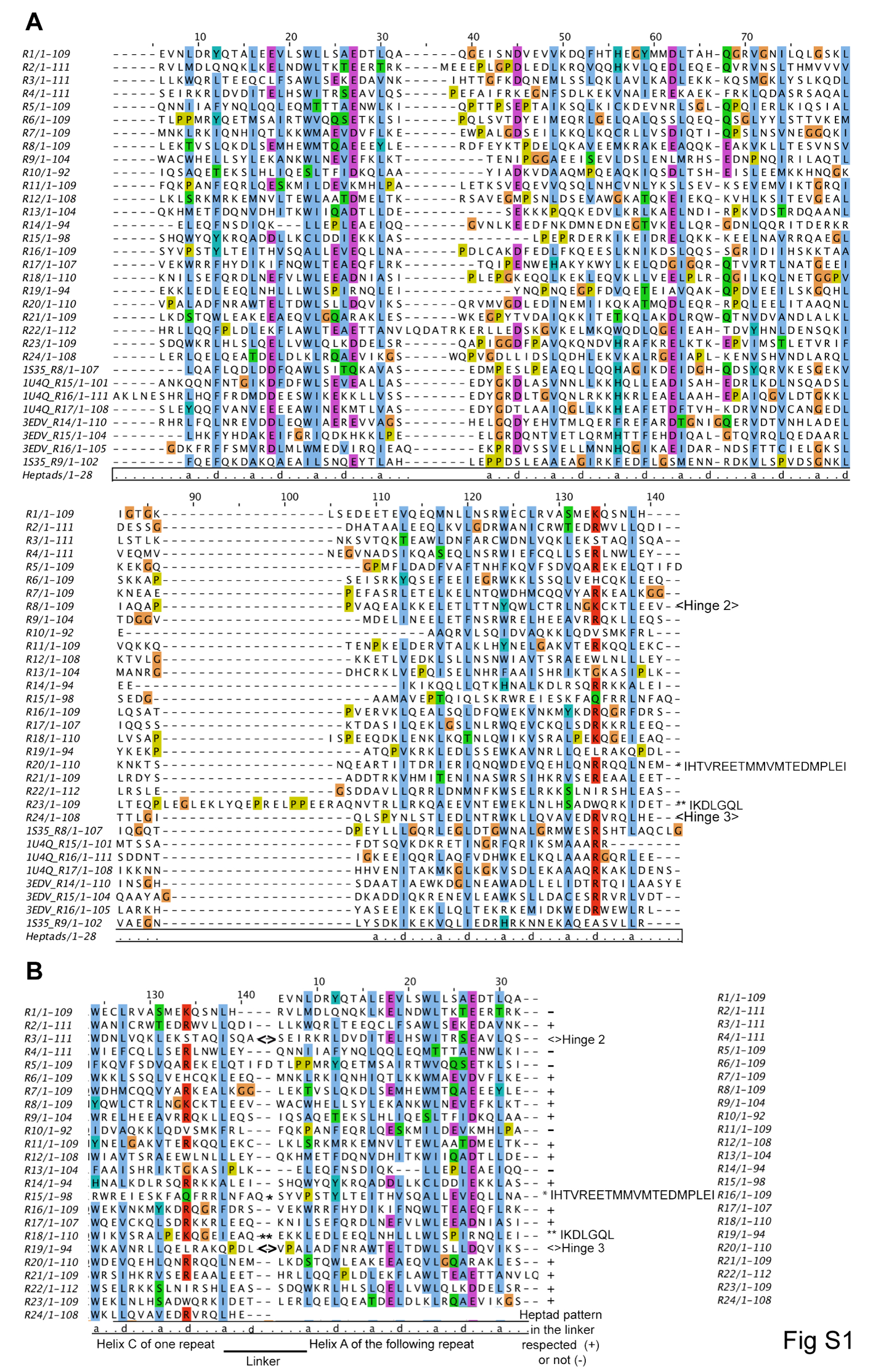

Supplement: Figure S1 — Sequence alignments. (A) Alignment of the 24 dystrophin repeats and the 8 spectrin repeats used by I-TASSER as templates. Repeats were aligned by ClustalW using default parameters. The alignment was visualized in Jalview and colored using the ClustalX color scheme. Each residue is marked by a specific color only when there is similarity across the repeats. In the bottom line, heptad motifs are indicated, showing the hydrophobic residues in the (a) and (d) positions. The presence of hinges or extra-sequences is mentioned at the end of the corresponding line. (B) The end of a repeat is aligned with the beginning of the following repeat to help visualize the linker within the tandem repeats. The heptad pattern is indicated as in (A). At the right, we indicate whether the heptad pattern is respected (+) or not (−) in the linker. Insertions are indicated by marking the hinges or the extra-sequences. (TIF) [file pone.0023819.s001.tif]

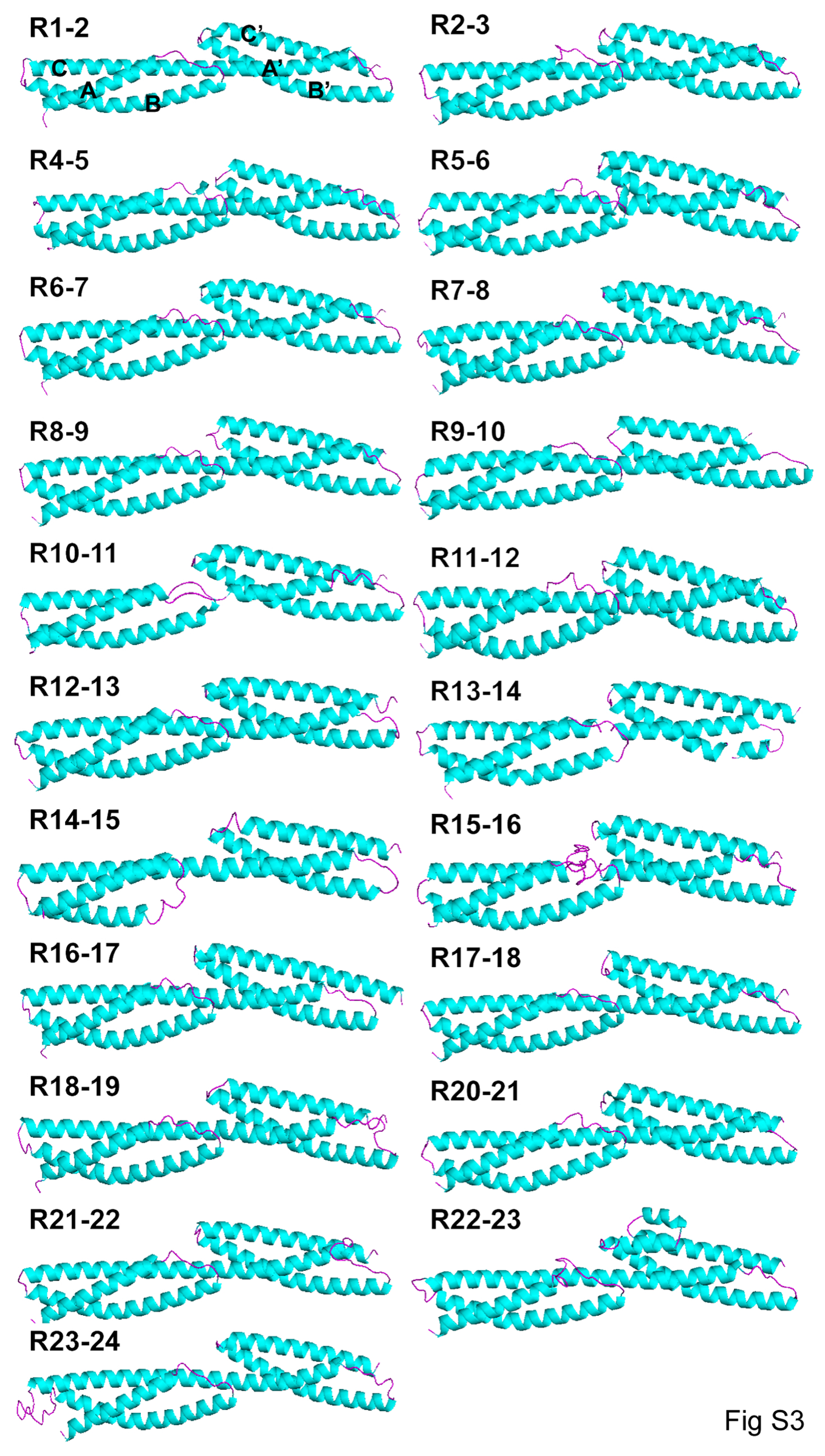

Supplement: Figure S2 — Three dimensional homology models of the 21 dystrophin tandem repeats obtained by I-TASSER. The models are represented as Cα backbone traces. Helical segments are colored in blue and loops are in pink. The N-terminal is on the left, the C-terminal is on the right, and the helix A of the N-terminal repeat of each tandem is at the front of the image. As a reminder, the nomenclature of the helices is given for the R1-2 model. A, B and C are the helices of the first repeat, and A’, B’ and C’ are the helices of the second repeat of the tandem. (TIF) [file pone.0023819.s002.tif]

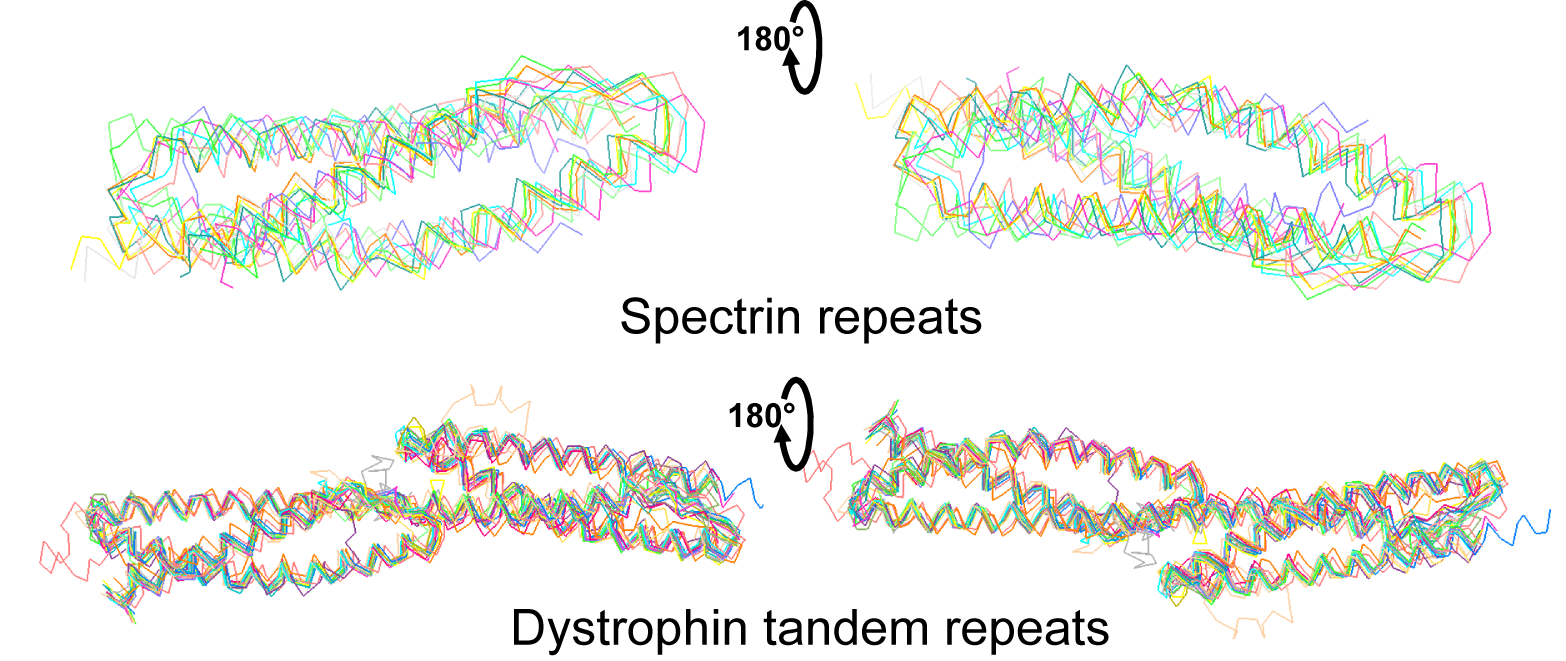

Supplement: Figure S3 — Superposition of the spectrin-repeat crystallographic structures and of the dystrophin tandem-repeat models. Top: Spectrin repeats shown were those used as templates by I-TASSER: chicken-brain a-spectrin repeats R15, R16 and R17 (1U4Q) [14], erythroid β-spectrin repeats R8 and R9 (1S35) [13] and β2-spectrin repeats R14, R15 and R16 (3EDV) [15]. Bottom: superposition of the dystrophin tandem repeats modeled with I-TASSER. The figure was made using PyMOL. (TIF) [file pone.0023819.s003.tif]

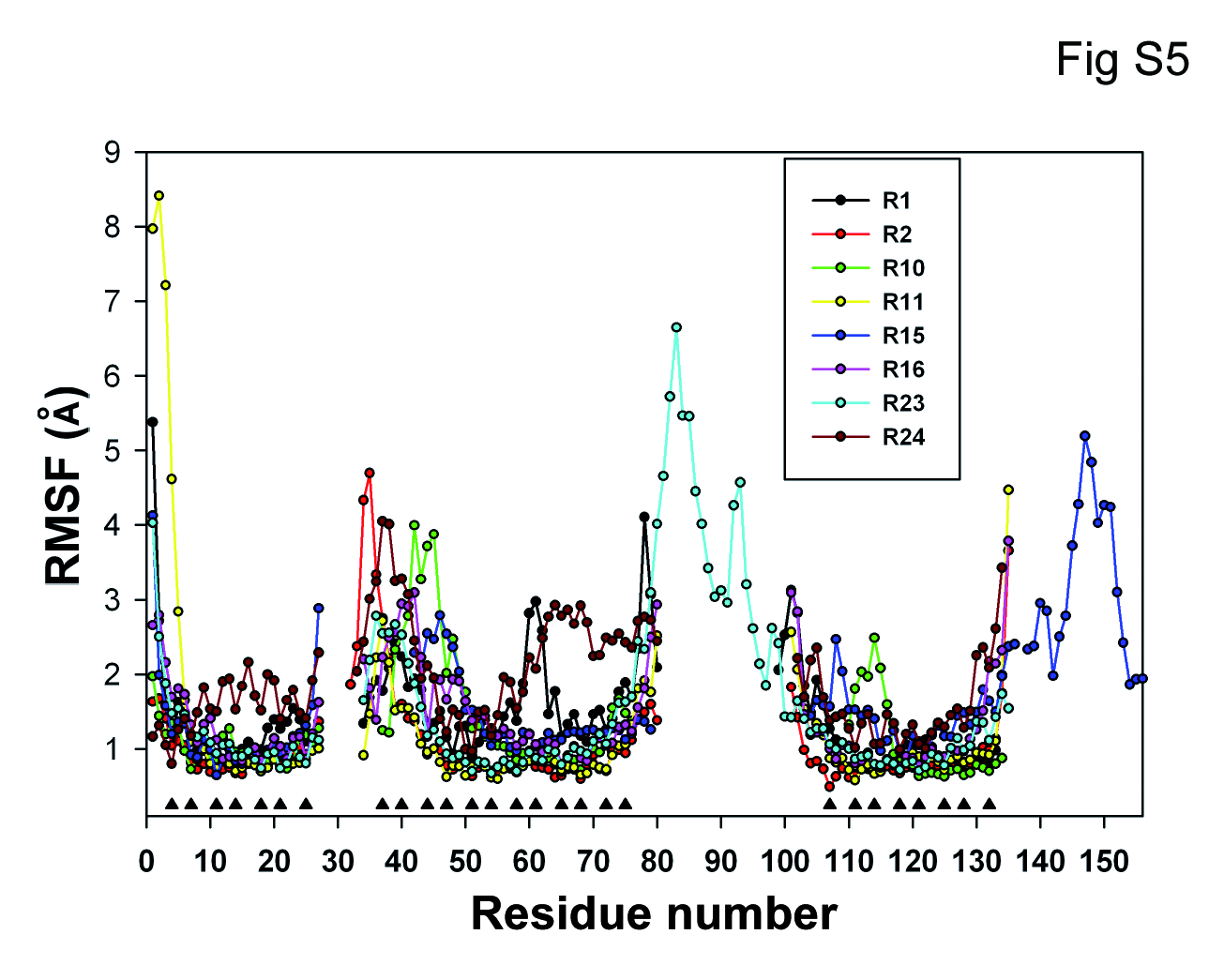

Supplement: Figure S4 — Quality assessment of the molecular dynamics relaxation of four tandem repeats. The residue-by-residue backbone fluctuation profile (RMSF) of the eight repeat units R1, R2, R10, R11, R15, R16, R23 and R24 is shown with the primary sequence of each isolated repeat aligned according to the heptad pattern. The (a) and (d) residues are marked with black triangles. (TIF) [file pone.0023819.s004.tif]

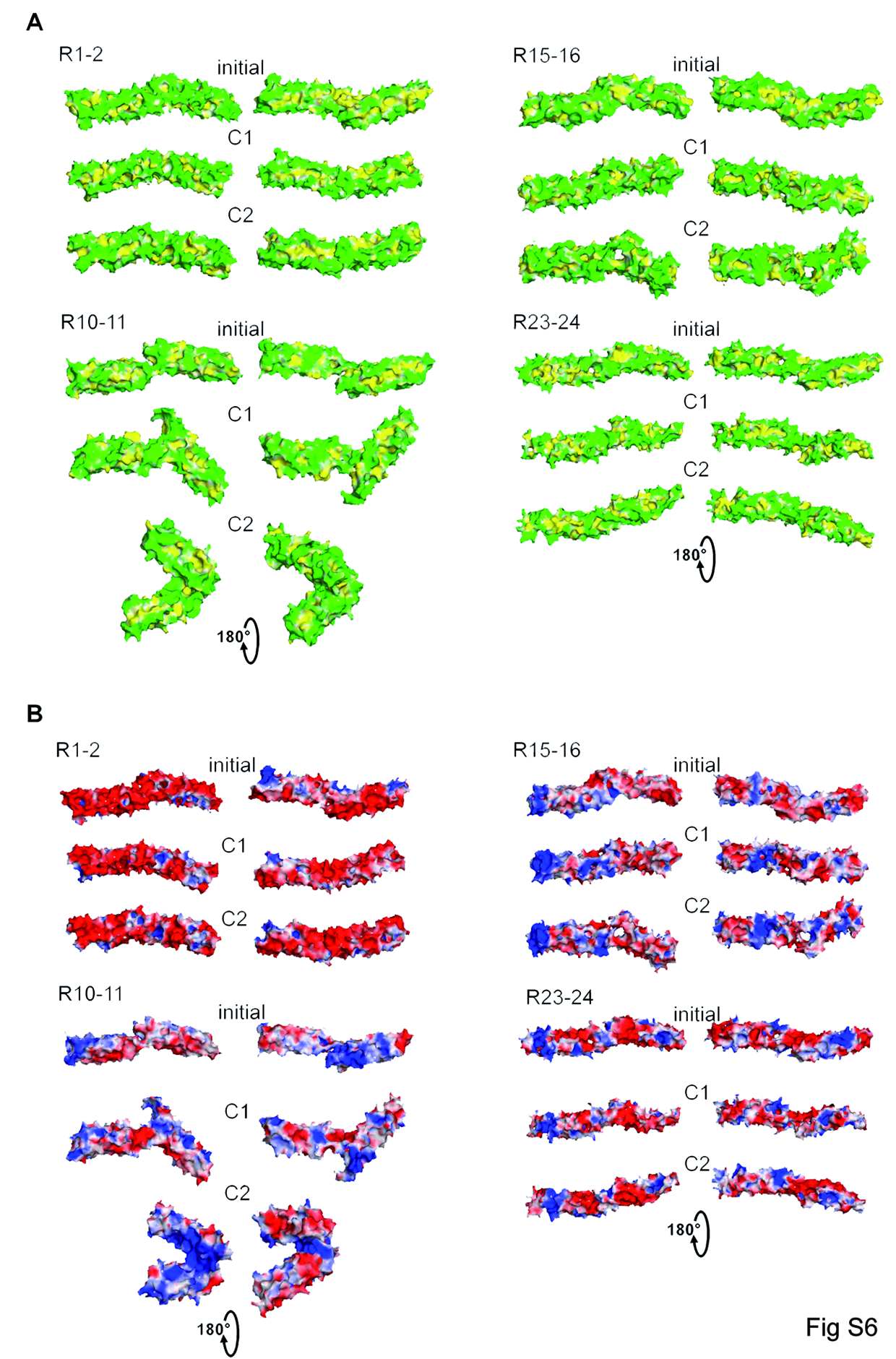

Supplement: Figure S5 — Influence of the molecular dynamics relaxation on the hydrophobicity and electrostatics of the molecule surfaces. As in Figure 1B, for the image on the left of each model, the molecules are presented with the helix A in front, the N-terminal end on the left and the C-terminal on the right. The right-hand image of each model is rotated 180° along the molecule's long axis, as indicated on the bottom. The initial model and the snapshots closest to the center of each cluster (C1 and C2) are shown in both cases. (A) Molecular hydrophobicity potential surfaces calculated with PLATINUM are shown using PyMOL. The hydrophobicity scale is green-white-yellow, with green representing the most hydrophilic regions and yellow the most hydrophobic. (B) Representation of the electrostatic potential projected on the solvent accessible surface of the dystrophin tandem repeats. Each model was colored using the APBS electrostatic potential calculated for an ionic strength of 50 mM, and the surface colors were clamped at -3 (red) and +3 (blue) kTe-1. (TIF) [file pone.0023819.s005.tif]

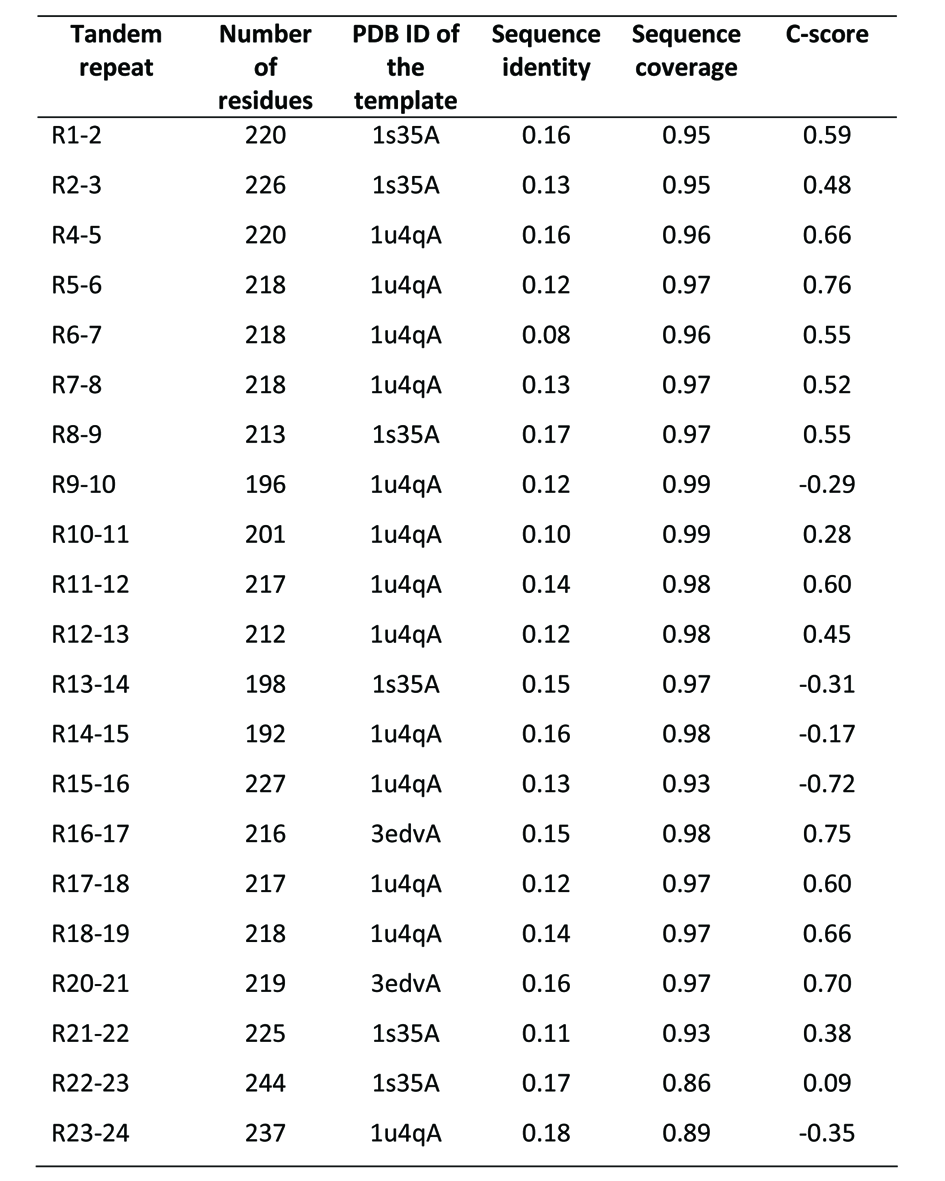

Supplement: Table S1 — I-TASSER statistics for the tandem-repeat models of the dystrophin central rod domain. (TIF) [file pone.0023819.s006.tif]

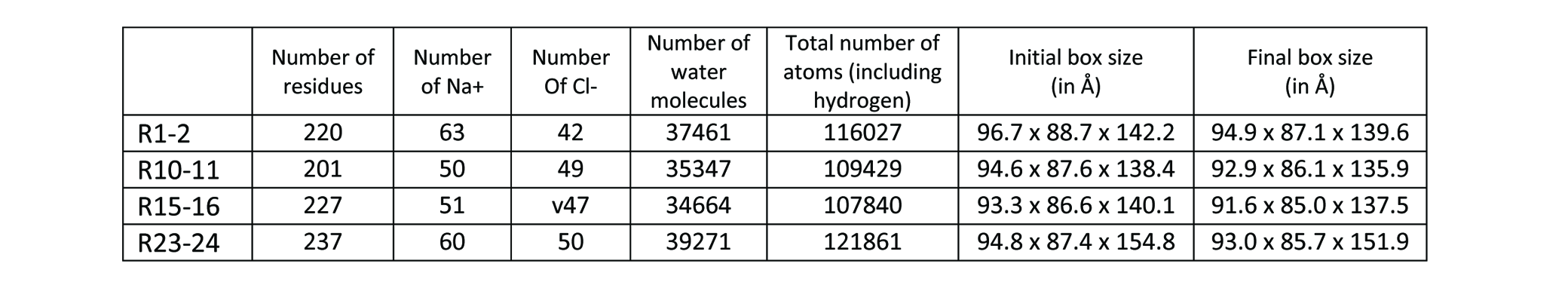

Supplement: Table S2 — Simulated system dimensions for the molecular dynamics study of the four tandem-repeats, R1-2, R10-11, R15-16 and R23-24. (TIF) [file pone.0023819.s007.tif]
