# Supplementary material for: Computational Study of the Human Dystrophin Repeats: Interaction Properties and Molecular Dynamics
Source: PLoS One. 2011 Aug 25;6(8):e23819. doi: 10.1371/journal.pone.0023819 (PMC3162007; doi:10.1371/journal.pone.0023819)
Supplement: Annex S1 — Quality assessment of homology modeling of all tandem repeats of the human dystrophin rod domain. For each model, the sequence and an image of the model as it appeared in Fig. 1 are shown first and second, respectively. Shown next are the results from PROCHECK, with the Z score and the graph of the energy for each residue in the sequence. The Verify3D results follow, along with the Ramachandran plot from PROCHECK. This is completed by an image of the model, with the residues in the disallowed regions of the Ramachandran plot colored in red. (PDF) [file pone.0023819.s008.pdf]

R1-2 model

EVNLDRYQTALEEVLSWLLSAEDTLQAQGEISNDVEVVKDQFHTHEGYMMDLTAHQGRV
GNILQLGSKLIGTGKLSEDEETEVQEQMNLLNSRWECLRVASMEKQSNLHRVLMDLQNQ
KLKELNDWLTKTEERTRKMEEEPLGPDLEDLKRQVQQHKVLQEDLEQEQVRVNSLTHMV
VVVDESSGDHATAALEEQLKVLGDRWANICRWTEDRWVLLQDI

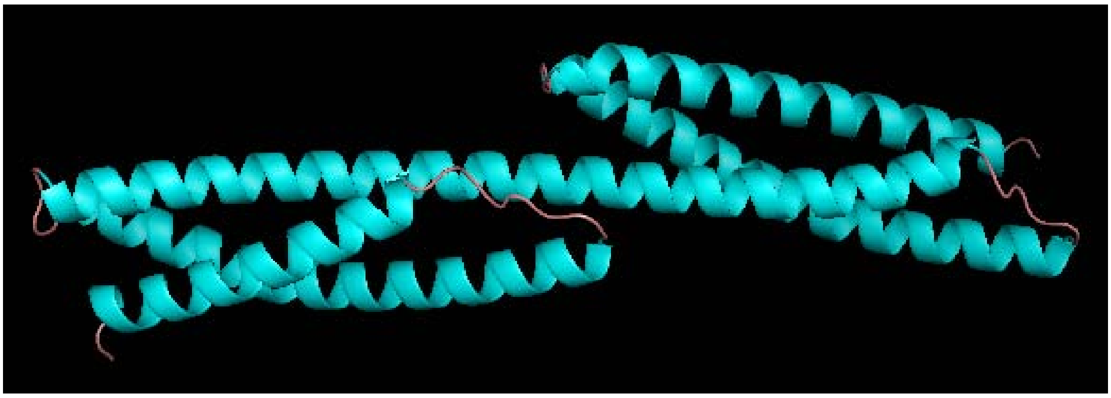

R1 R2

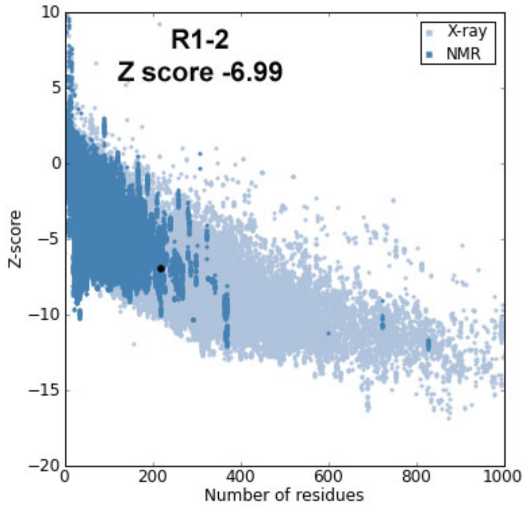

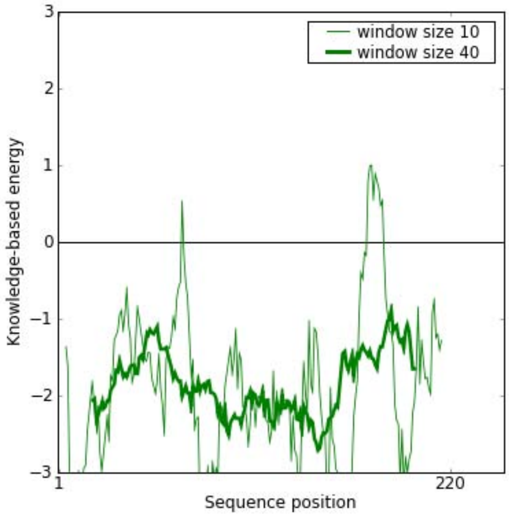

Verify3D

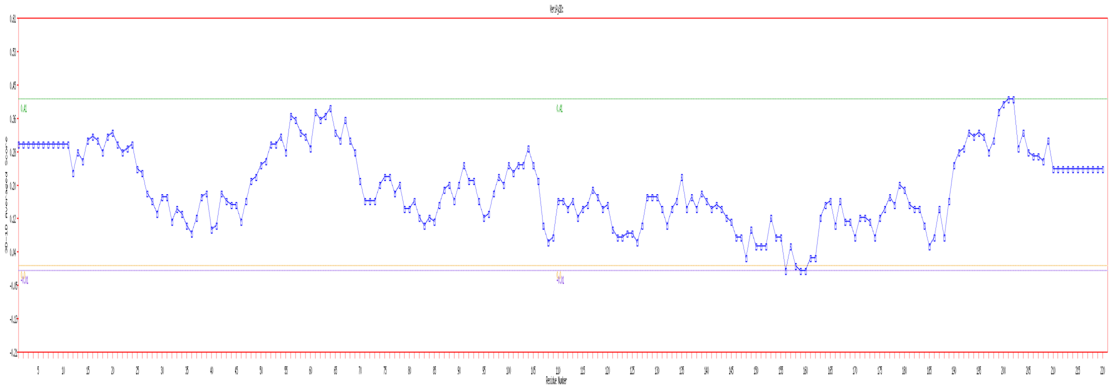

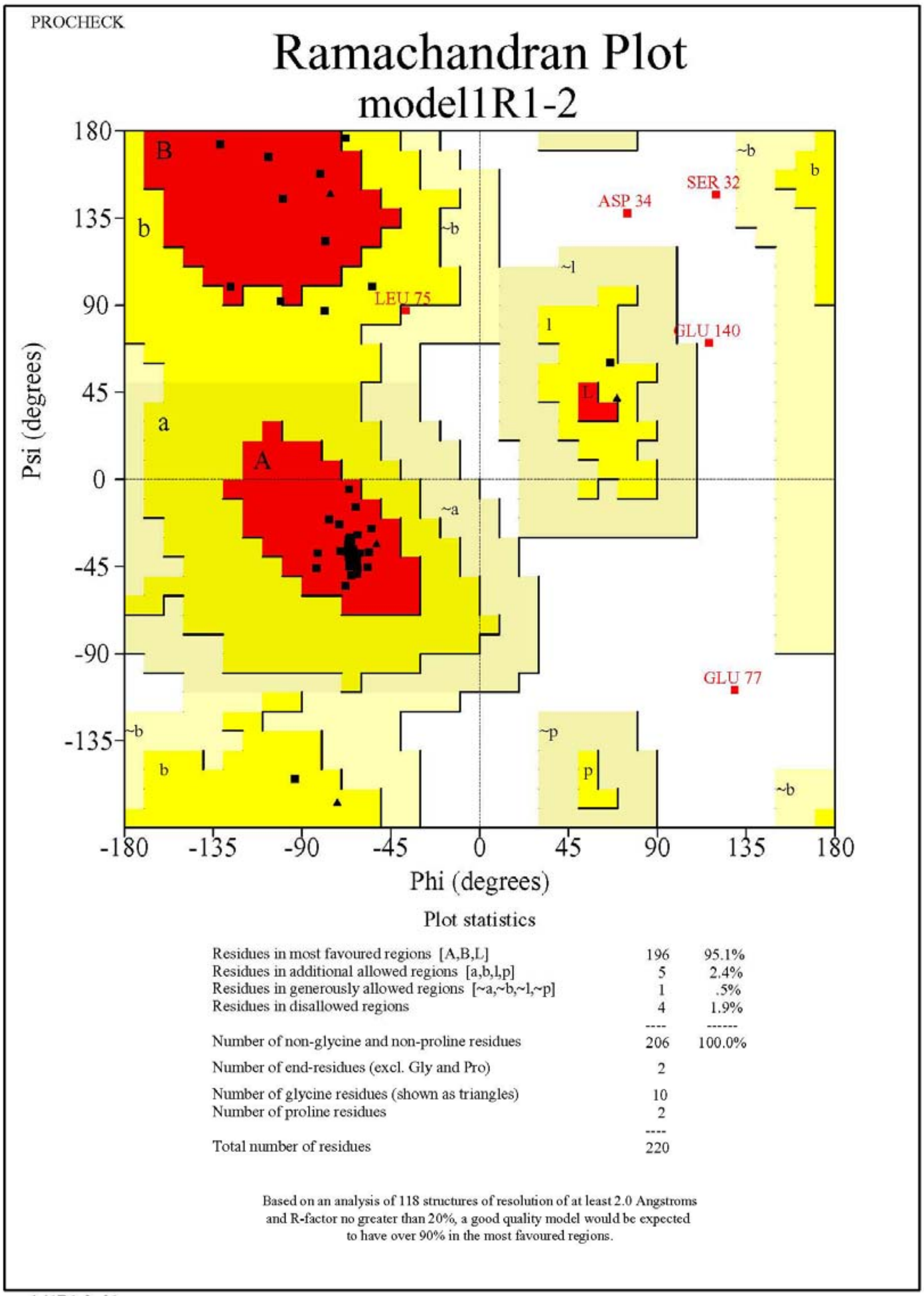

model1R1-2_01.ps

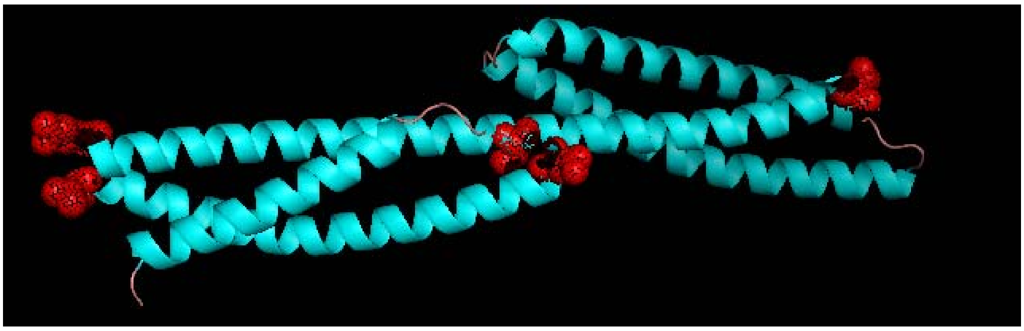

Residues in disallowed regions the Ramachanran plot are coloured in red and as dots.

R2-3 Model

RVLMDLQNQKLKELNDWLTKTEERTRKMEEEPLGPDLEDLKRQVQQHKVLQEDLEQEQV
RVNSLTHMVVVVDESSGDHATAALEEQLKVLGDRWANICRWTEDRWVLLQDILLKWQRL
TEEQCLFSAWLSEKEDAVNKIHTTGFKDQNEMLSSLQKLAVLKADLEKKKQSMGKLYSL
KQDLLSTLKNKSVTQKTEAWLDNFARCWDNLVQKLEKSTAQISQA

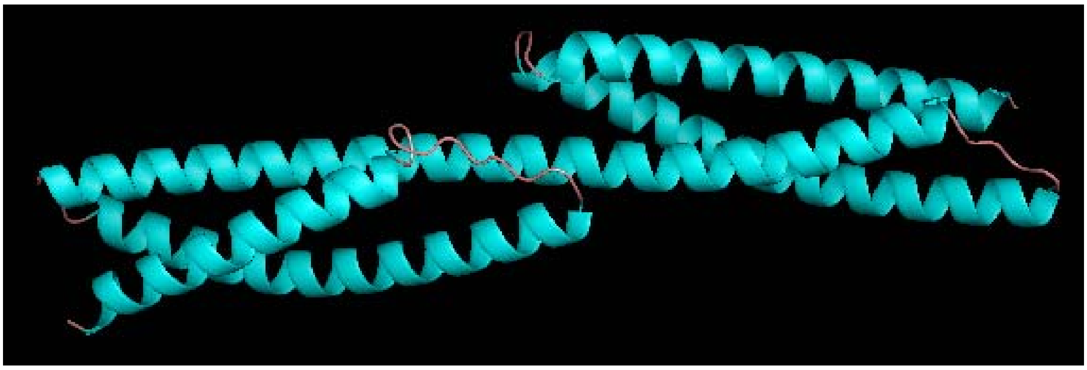

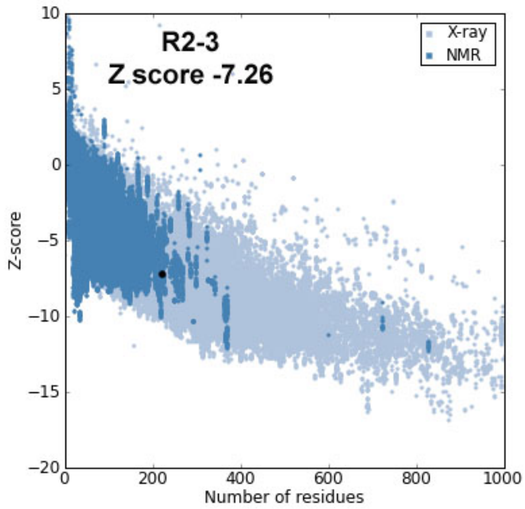

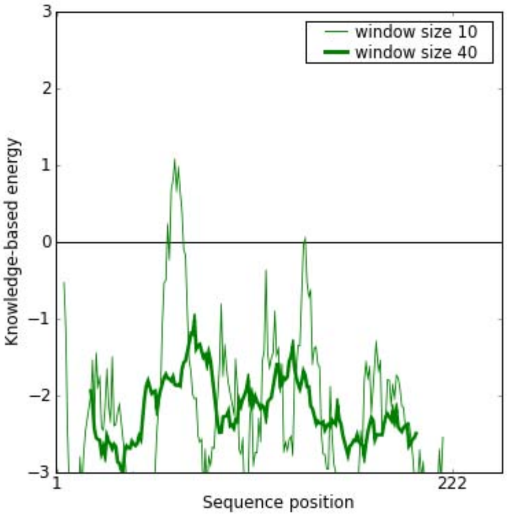

Verify3D structure evaluation

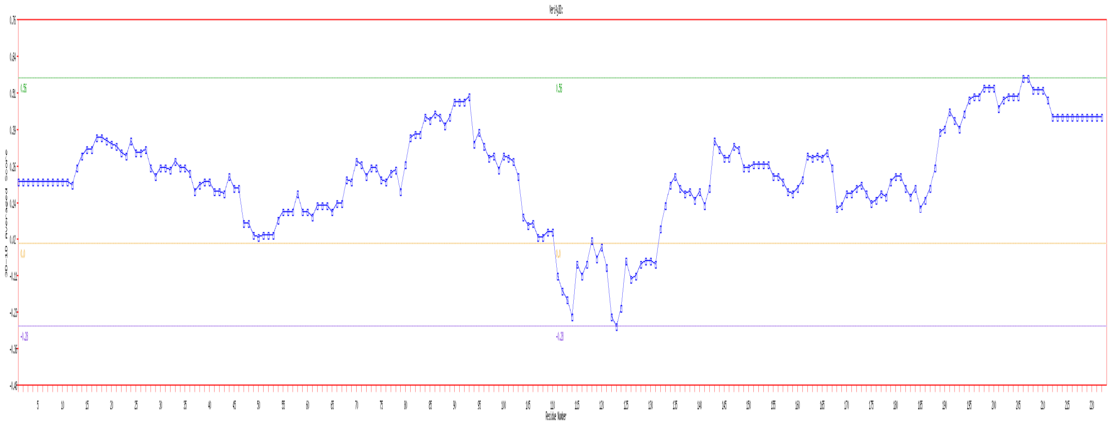

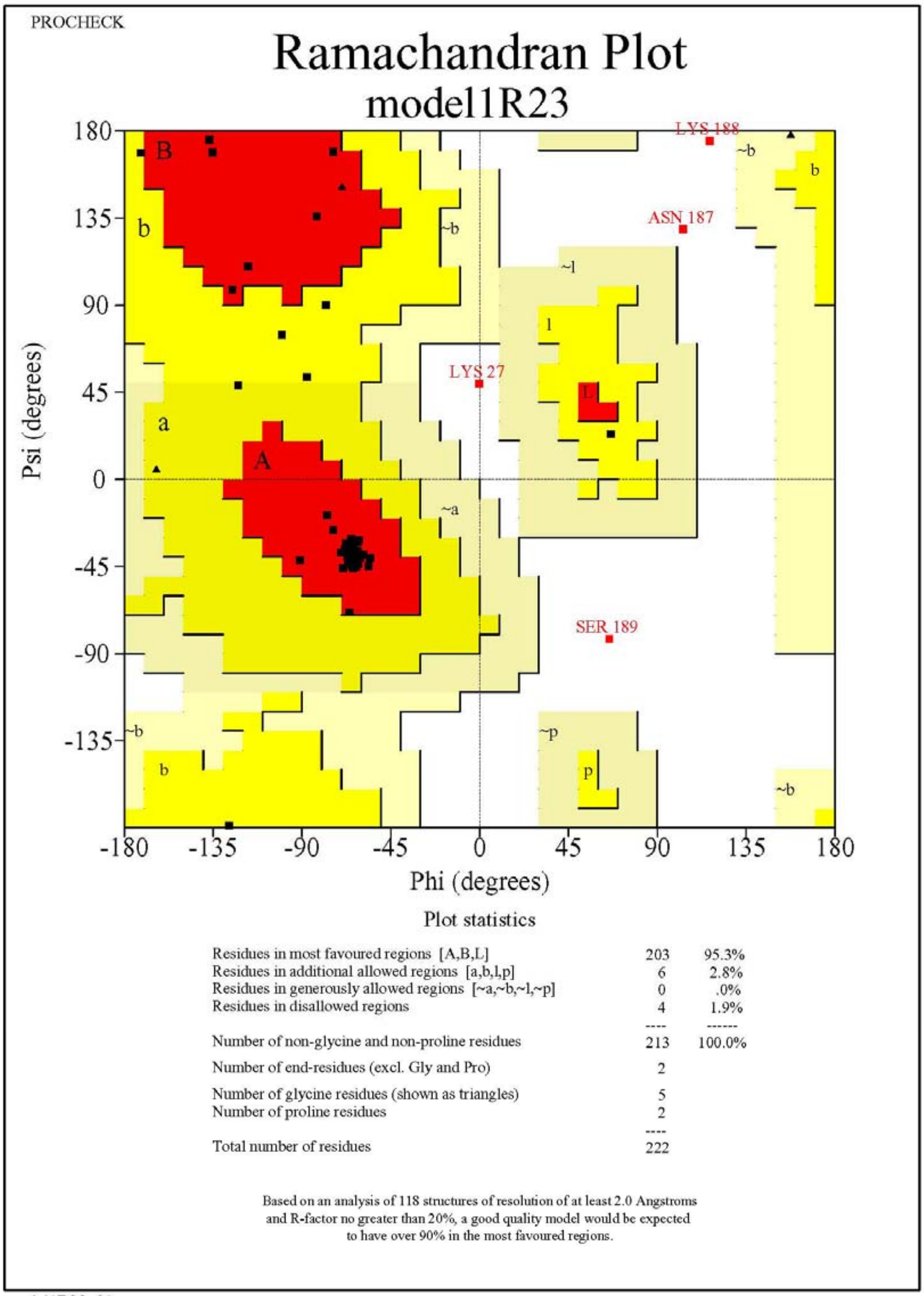

model1R23_01.ps

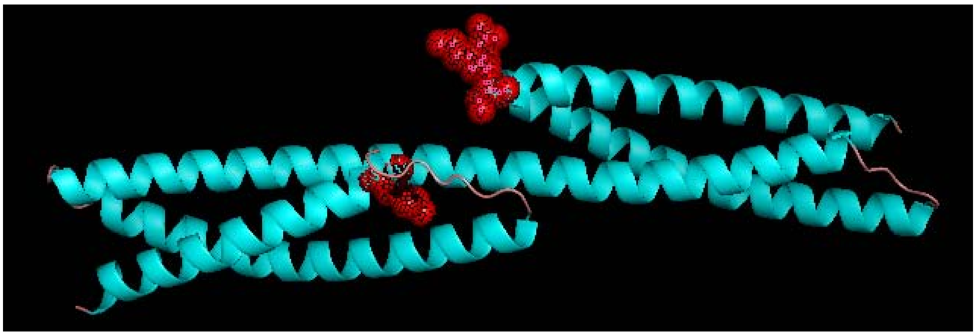

Residues in disallowed regions in the Ramachandran plot are coloured in red

Fig S2

R4-5 model

SEIRKRLDVDITELHSWITRSEAVLQSPEFAIFRKEGNFSDLKEKVNAIEREKAEKFRKLQDASR
SAQALVEQMVNEGVNADSIKQASEQLNSRWIEFCQLLSERLNWLEYQNNIIAFYNQLQQLEQ
MTTTAENWLKIQPTTPSEPTAIKSQLKICKDEVNRLSGLQPQIERLKIQSIALKEKGQGPMFLD
ADFVAFTNHFKQVFSDVQAREKELQTIFD

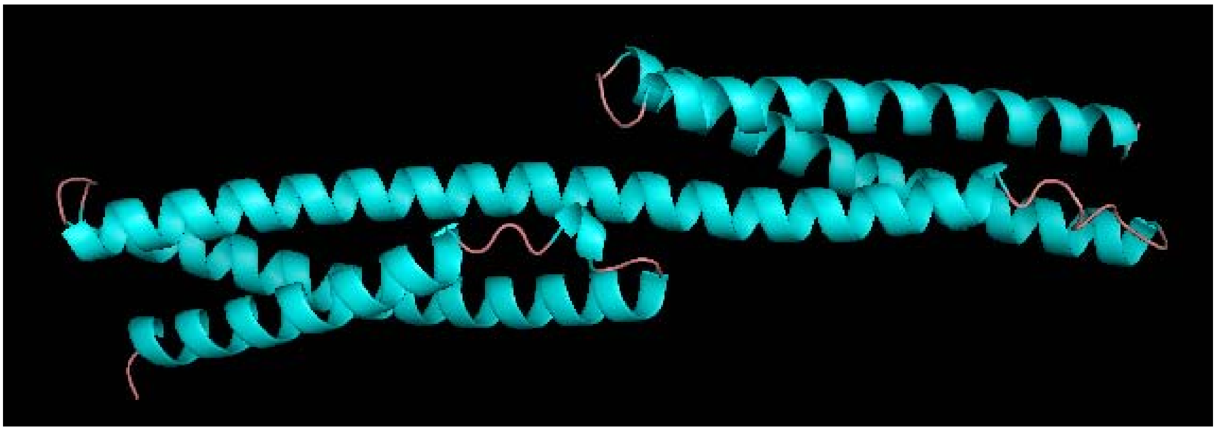

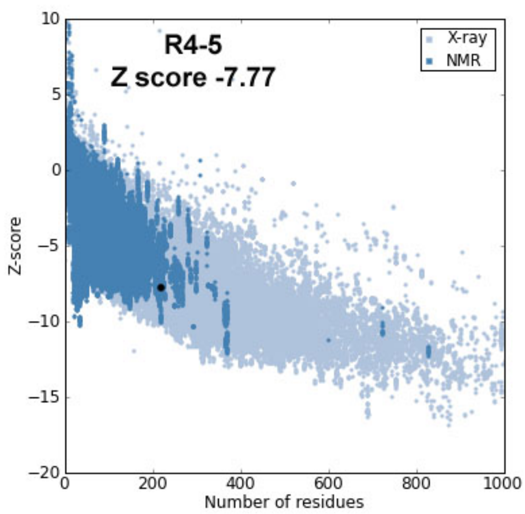

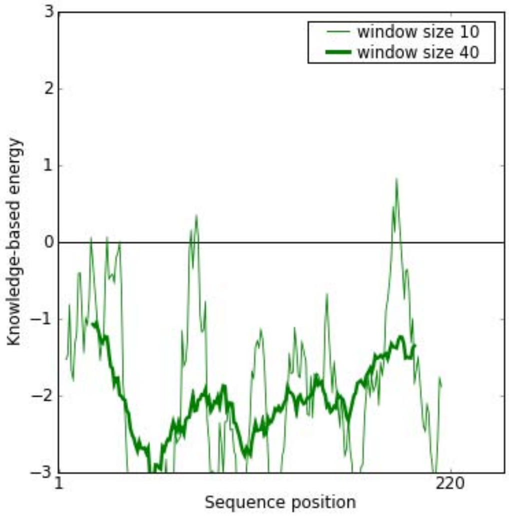

Verify3D structure evaluation

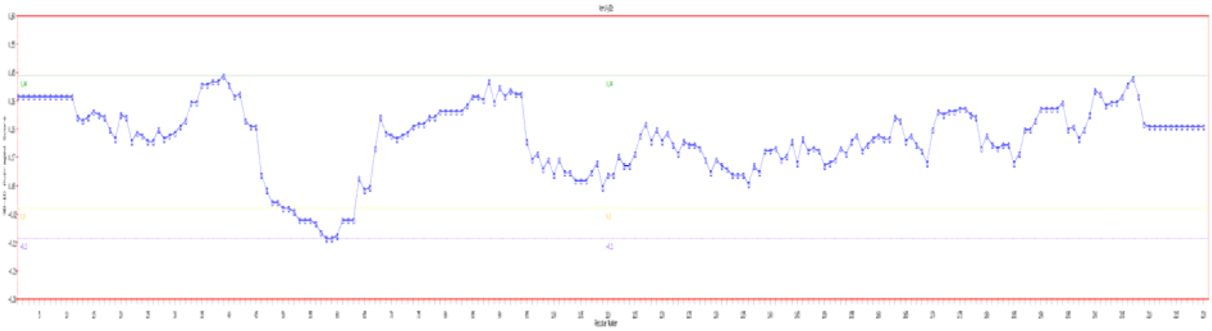

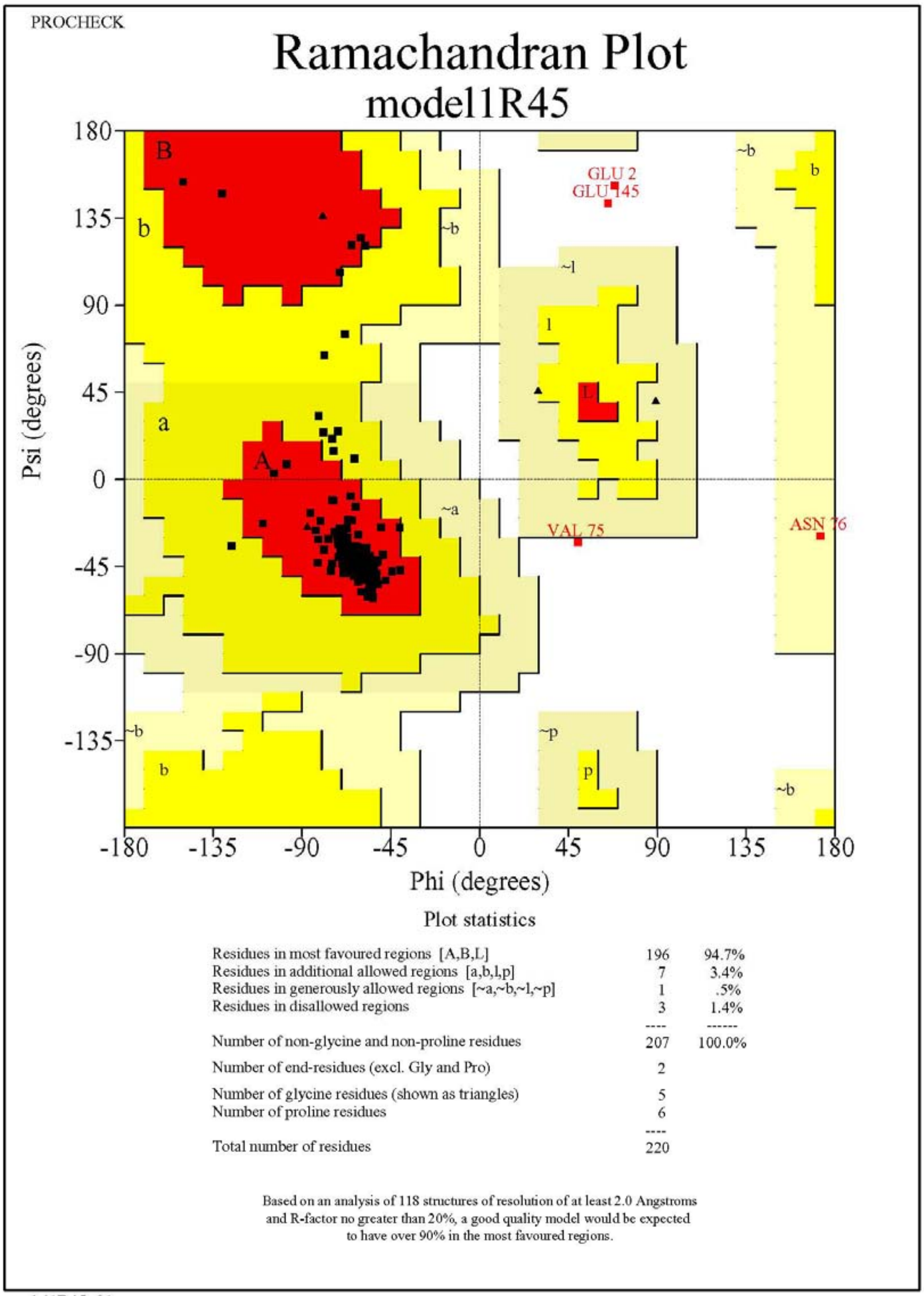

model1R45_01.ps

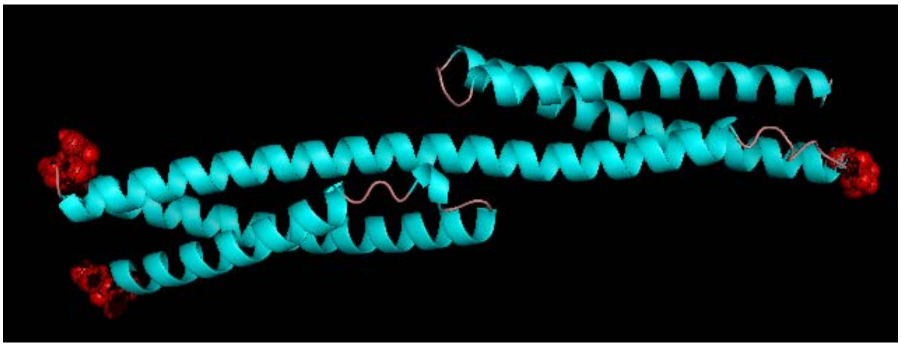

Residues of disallowed regions of the Ramachandran plot are in red

R5-6 model

QNNIIAFYNQLQQLEQMTTTAENWLKIQPTTPSEPTAIKSQLKICKDEVNRLSGLQPQIERLKIQ
SIALKEKGQGPMFLDADFVAFTNHFKQVFSDVQAREKELQTIFDTLPPMRYQETMSAIRTWV
QQSETKLSIPQLSVTDYEIMEQRLGELQALQSSLQEQQSGLYYLSTTVKEMSKKAPSEISRKYQ
SEFEEIEGRWKKLSSQLVEHCQKLEEQ

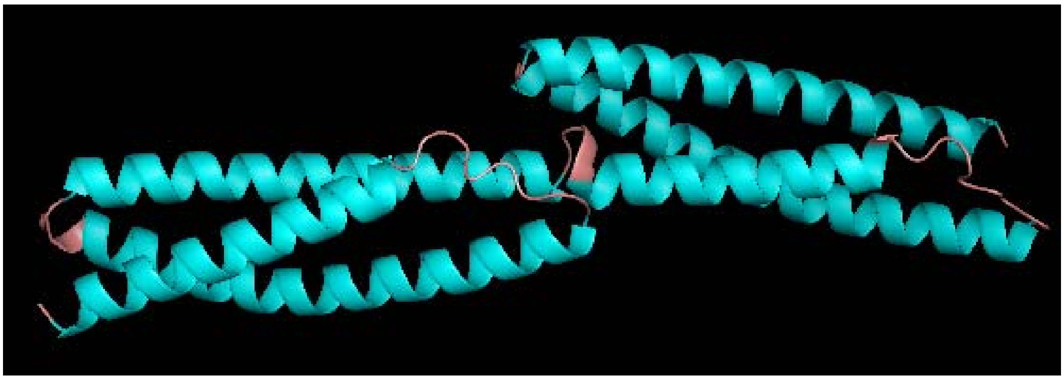

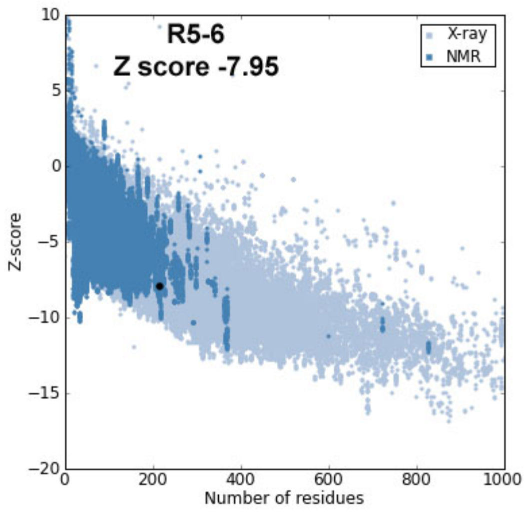

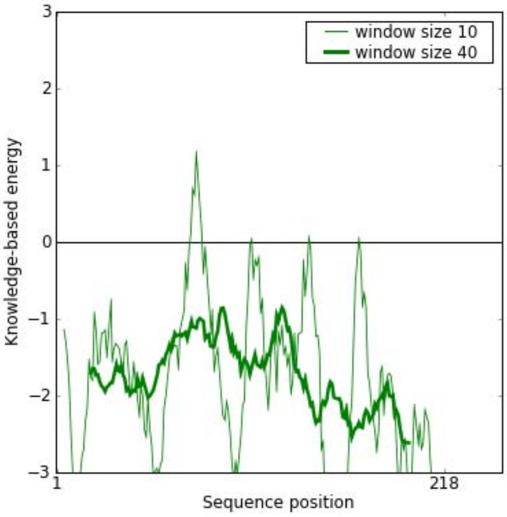

Verify3D structural evaluation

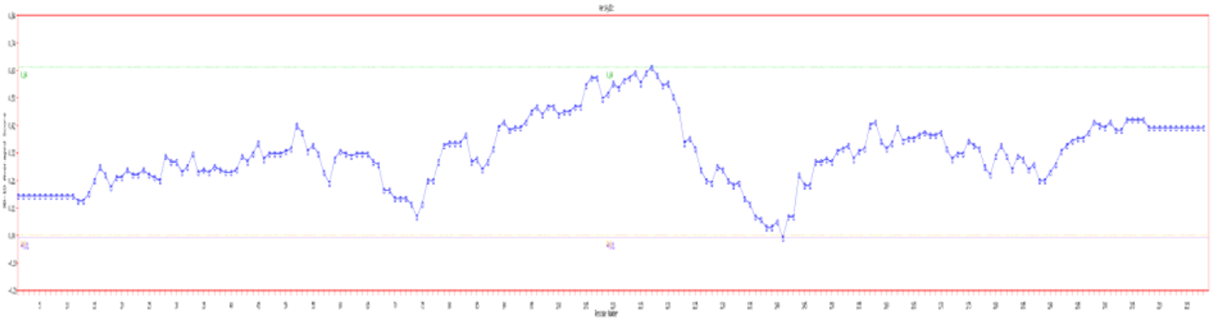

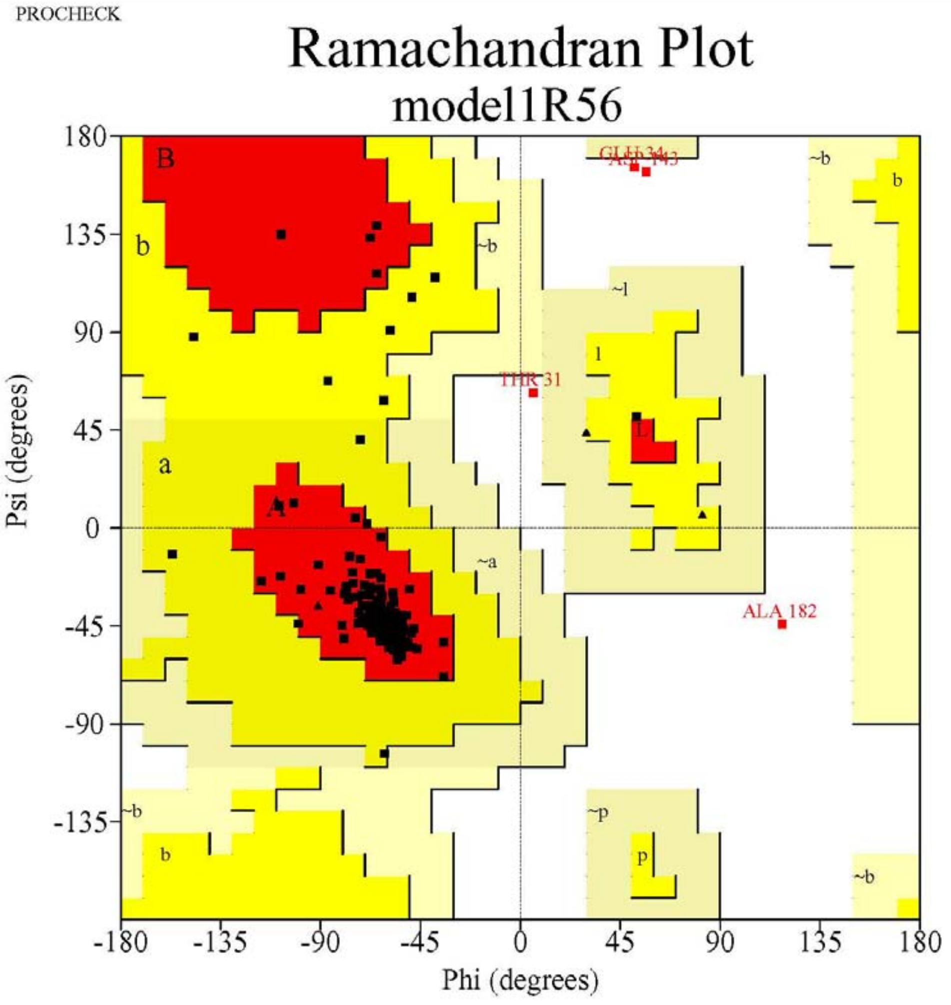

Plot statistics

Residues in most favoured regions [A,B,L]	188	93.5%
Residues in additional allowed regions [a,b,l,p]	9	4.5%
Residues in generously allowed regions [~a,~b,~l,~p]	0	.0%
Residues in disallowed regions	4	2.0%
	----	------
Number of non-glycine and non-proline residues	201	100.0%
Number of end-residues (excl. Gly and Pro)	2	
Number of glycine residues (shown as triangles)	6	
Number of proline residues	9	

Total number of residues	218	

Based on an analysis of 118 structures of resolution of at least 2.0 Angstroms and R-factor no greater than 20%, a good quality model would be expected to have over 90% in the most favoured regions.

model1R56_01.ps

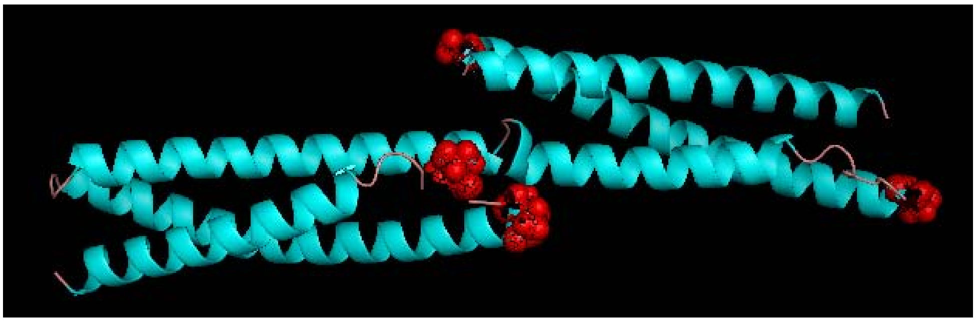

Residues in disallowed regions of the Ramachandran plot are in red

R6-7 model

TLPPMRYQETMSAIRTWVQQSETKLSIPQLSVTDYEIMEQRLGELQALQSSLQEQQSGLYYLS
TTVKEMSKKAP-SEISRKYQSEFEEIEGRWKKLSSQLVEHCQKLEEQ
MNKLRKIQNHIQTLKKWMAEVDVFLKEEWPALGDSEILKKQLKQCRLLVSDIQTIQPSLNSV
NEGGQKIKNEAEPEFASRLETELKELNTQWDHMCQQVYARKEALKGG

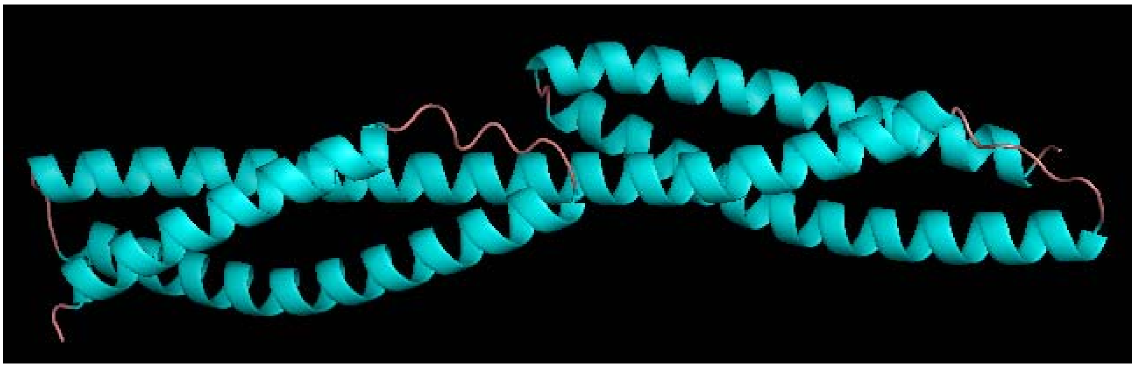

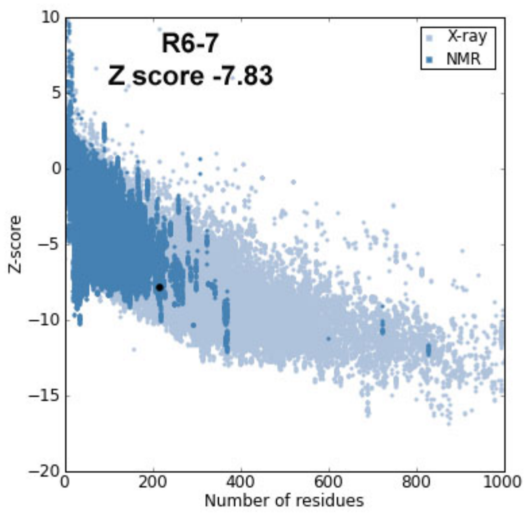

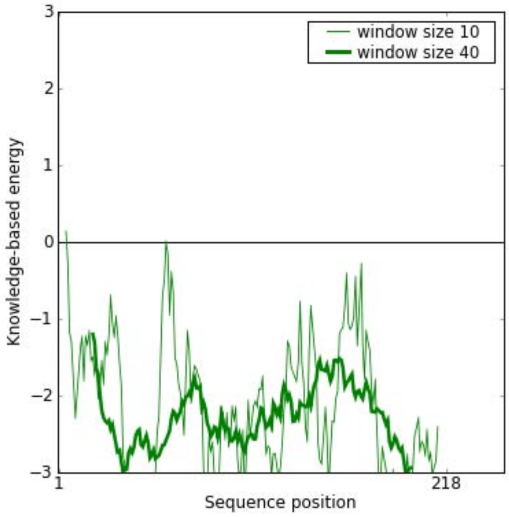

Verify3D structure evaluation

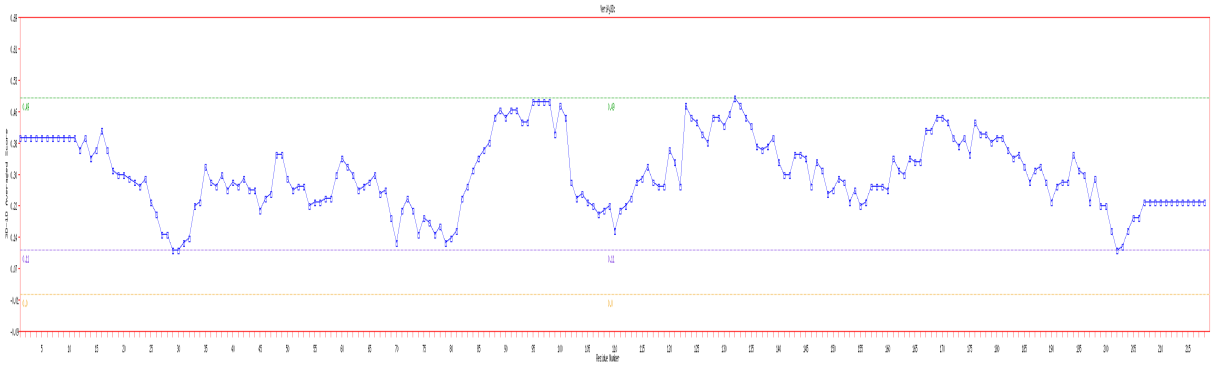

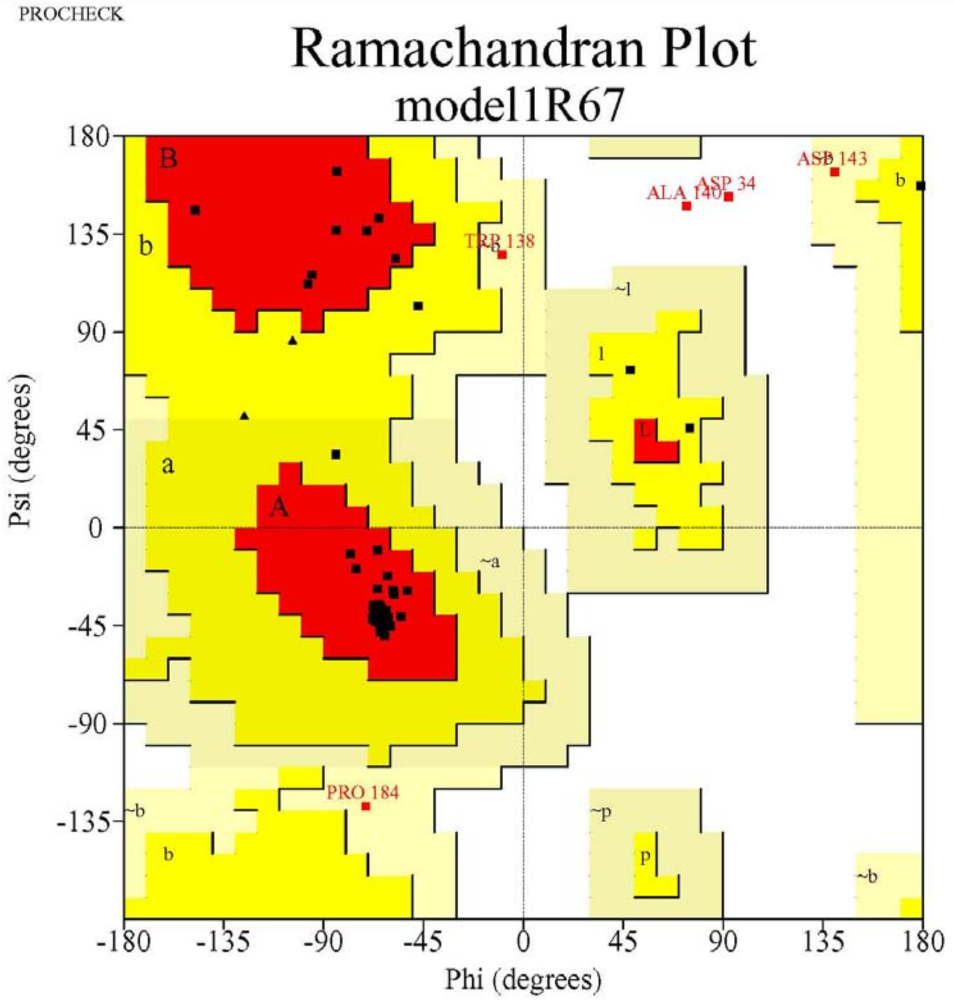

Plot statistics

Residues in most favoured regions [A,B,L]	193	95.5%
Residues in additional allowed regions [a,b,l,p]	5	2.5%
Residues in generously allowed regions [~a,~b,~l,~p]	2	1.0%
Residues in disallowed regions	2	1.0%
	----	------
Number of non-glycine and non-proline residues	202	100.0%
Number of end-residues (excl. Gly and Pro)	1	
Number of glycine residues (shown as triangles)	8	
Number of proline residues	7	

Total number of residues	218	

Based on an analysis of 118 structures of resolution of at least 2.0 Angstroms and R-factor no greater than 20%, a good quality model would be expected to have over 90% in the most favoured regions.

model1R67_01.ps

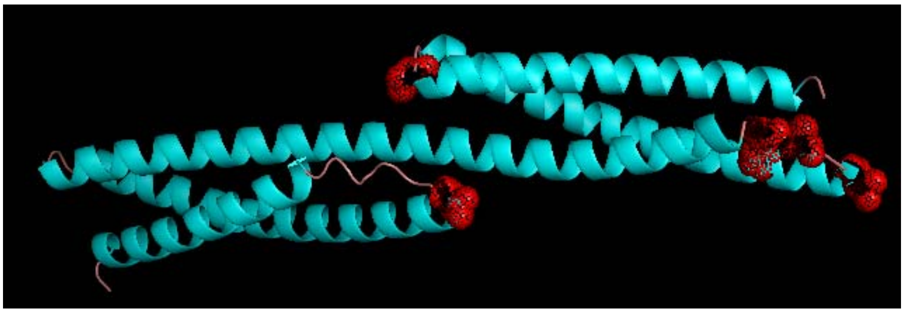

Residues in disallowed regions of the Ramachandran plot are in red

R7-8 model

MNKLRKIQNHIQTLKKWMAEVDVFLKEEWPALGDSEILKKQLKQCRLLVSDIQTIQPSLNSV
NEGGQKIKNEAE-PEFASRLETELKELNTQWDHMCQQVYARKEALKGG
LEKTVSLQKDLSEMHEWMTQAEEEYLERDFEYKTPDELQKAVEEMKRAKEEAQQKEAKVK
LLTESVNSVIAQAPPVAQEALKKELETLTTNYQWLCTRLNGKCKTLEEV

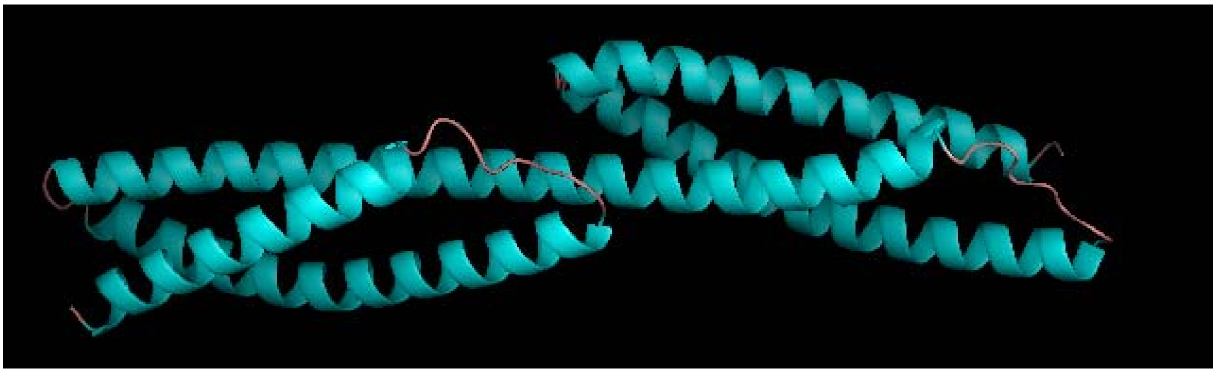

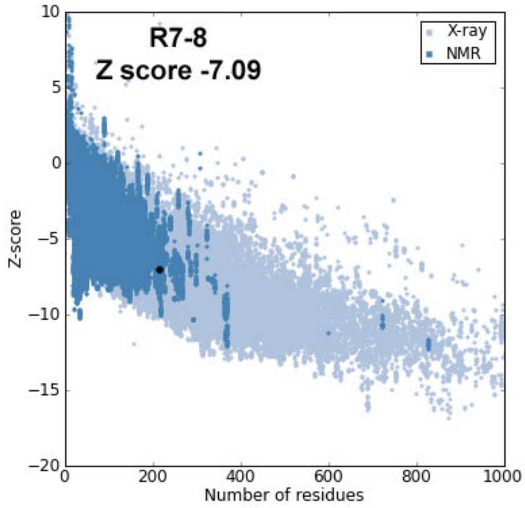

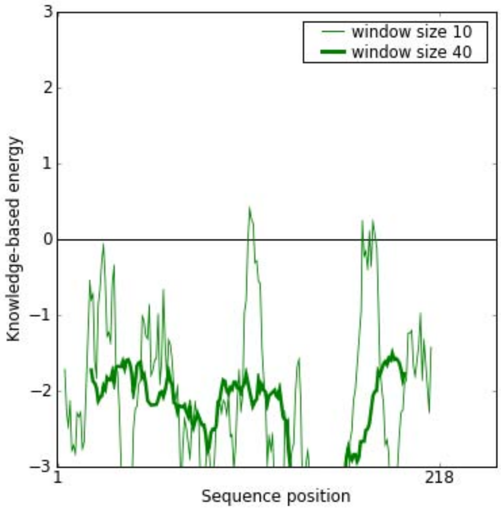

Verify3D structure evaluation

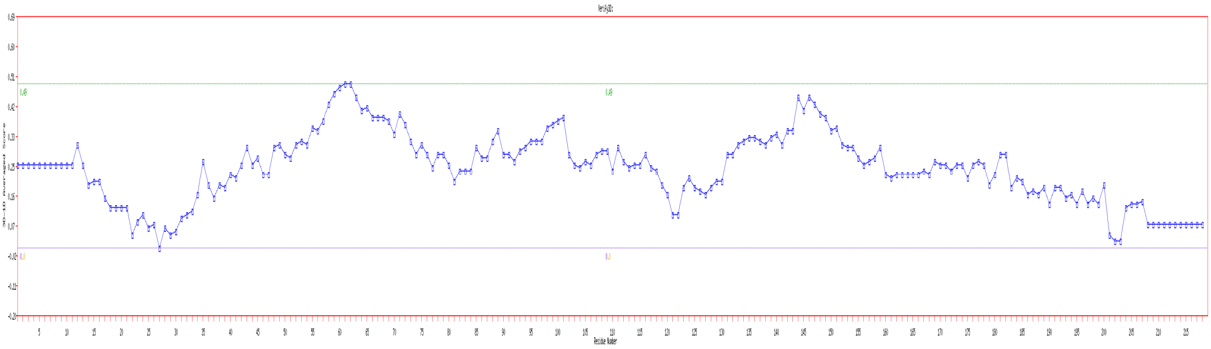

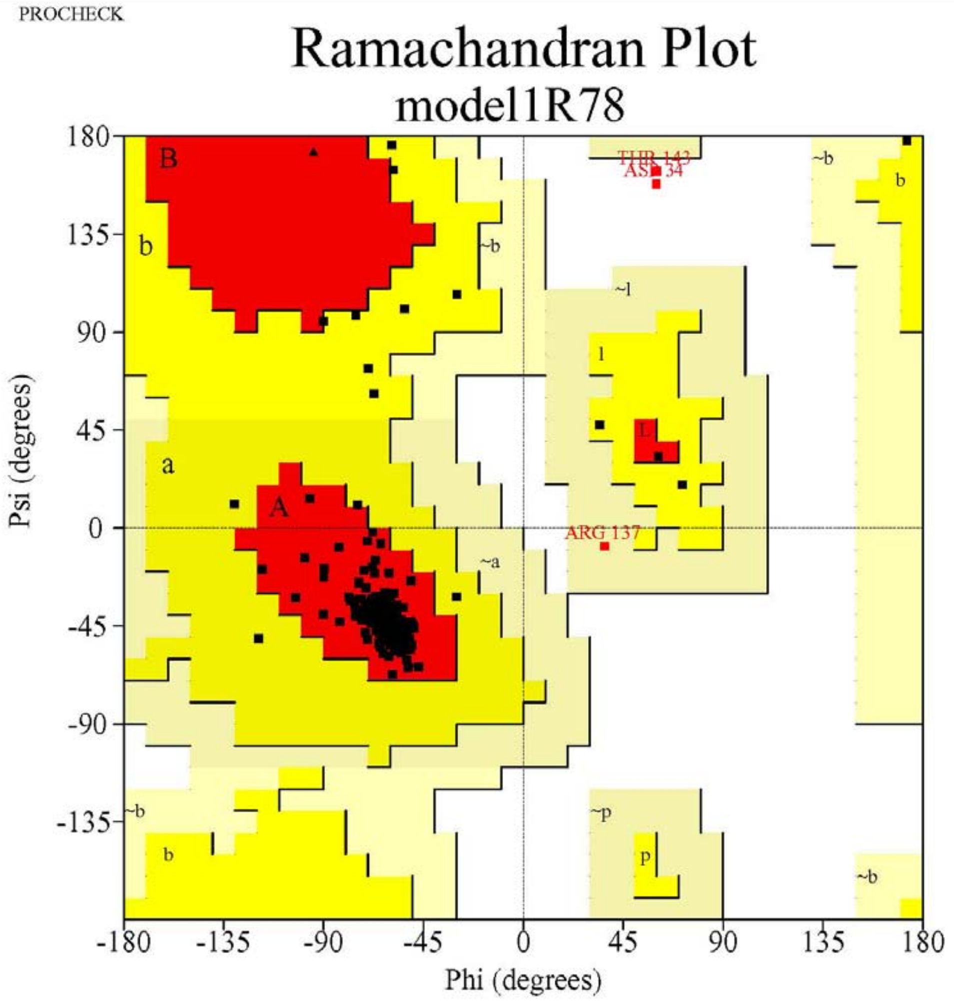

Plot statistics

Residues in most favoured regions [A,B,L]	189	92.6%
Residues in additional allowed regions [a,b,l,p]	12	5.9%
Residues in generously allowed regions [~a,~b,~l,~p]	1	.5%
Residues in disallowed regions	2	1.0%
	----	------
Number of non-glycine and non-proline residues	204	100.0%
Number of end-residues (excl. Gly and Pro)	2	
Number of glycine residues (shown as triangles)	6	
Number of proline residues	6	

Total number of residues	218	

Based on an analysis of 118 structures of resolution of at least 2.0 Angstroms and R-factor no greater than 20%, a good quality model would be expected to have over 90% in the most favoured regions.

model1R78_01.ps

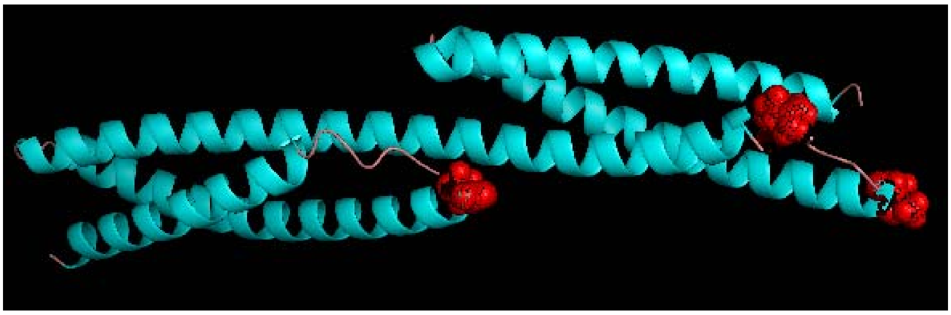

Residues in disallowed regions of the Ramachandran plot are in red

R8-9 model

LEKTVSLQKDLSEMHEWMTQAEEEYLERDFEYKTPDELQKAVEEMKRAKEEAQQKEAKVK
LLTESVNSVIAQAPPVAQEALKKELETLTTNYQWLCTRLNGKCKTLEEVWACWHELLSYL
EKANKWLNEVEFKLKTTENIPGGAEEISEVLDSLENLMRHSEDNPNQIRILAQTLTDGGV
MDELINEELETFNSRWRELHEEAVRRQKLLEQS

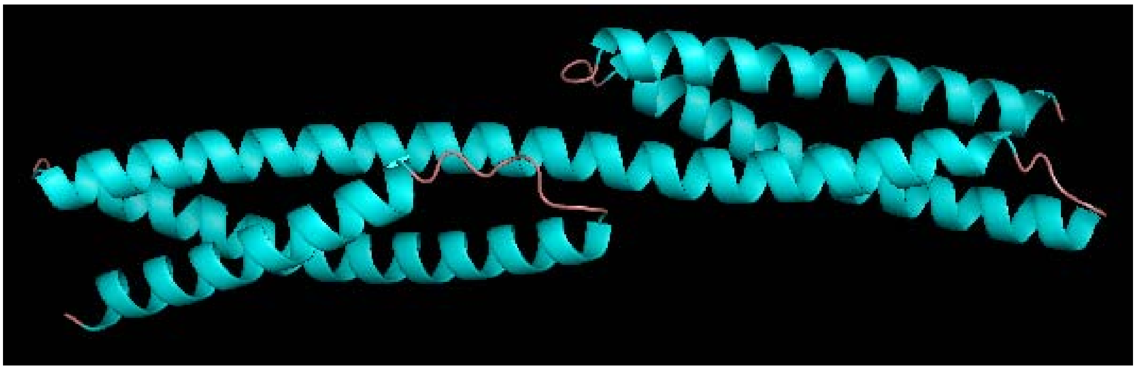

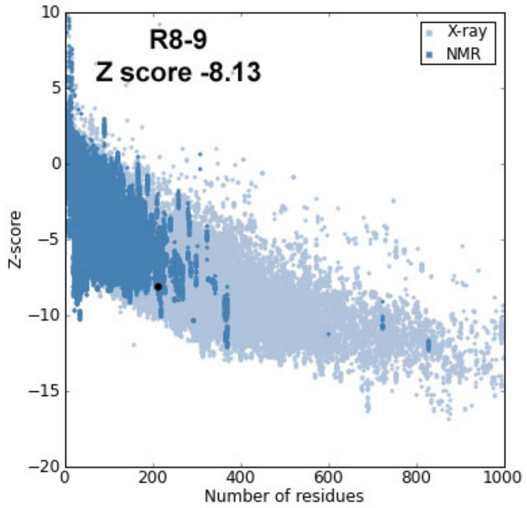

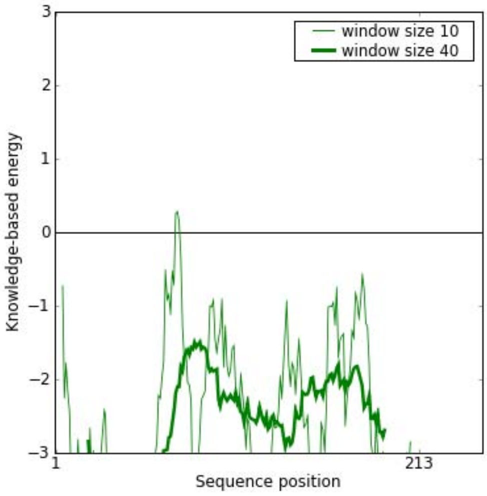

Verify3D structure evaluation

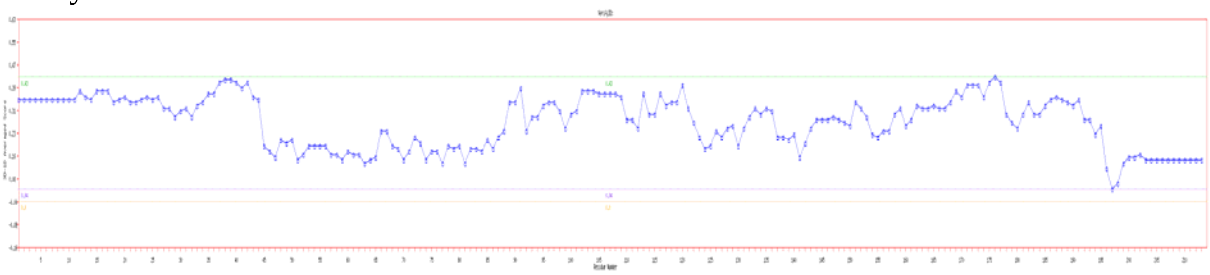

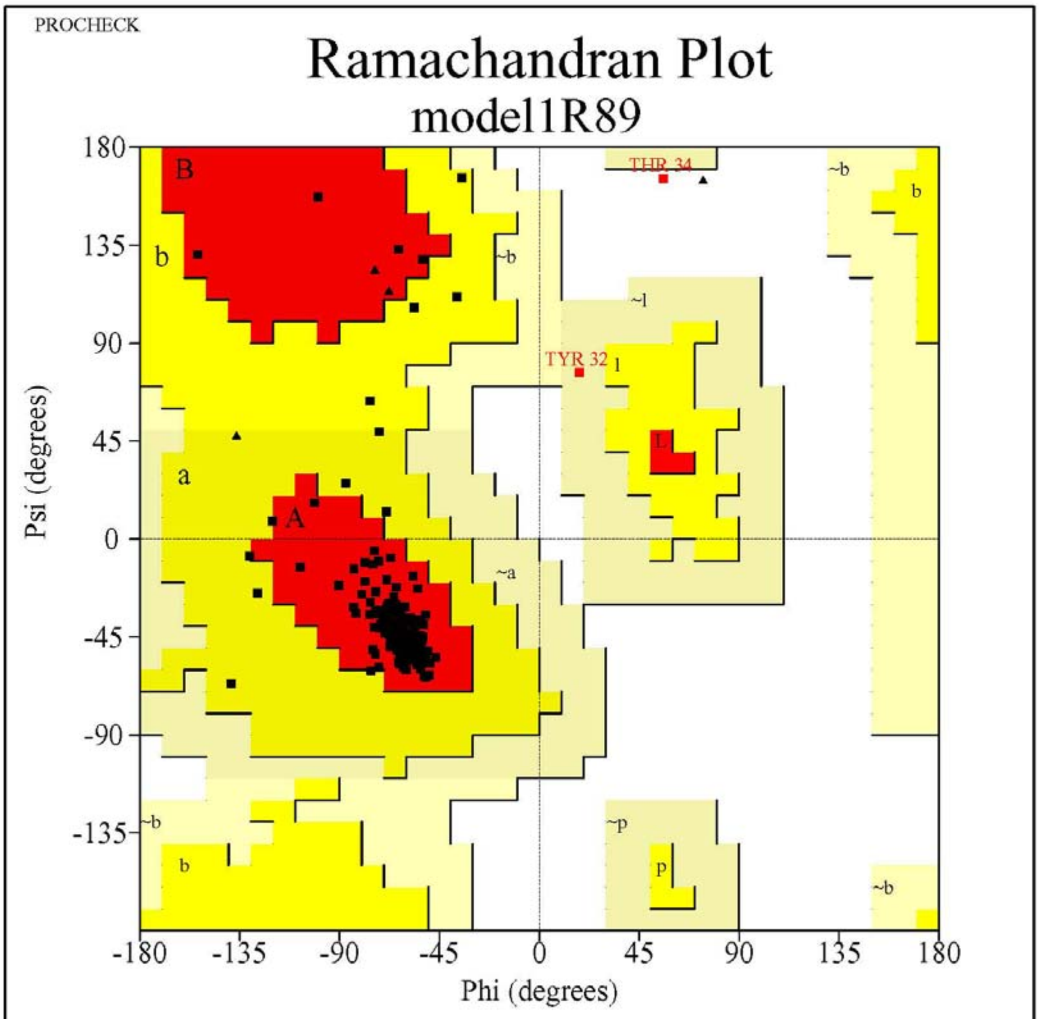

Plot statistics

Residues in most favoured regions [A,B,L]	188	93.5%
Residues in additional allowed regions [a,b,l,p]	11	5.5%
Residues in generously allowed regions [~a,~b,~l,~p]	1	.5%
Residues in disallowed regions	1	.5%
	----	------
Number of non-glycine and non-proline residues	201	100.0%
Number of end-residues (excl. Gly and Pro)	2	
Number of glycine residues (shown as triangles)	5	
Number of proline residues	5	

Total number of residues	213	

Based on an analysis of 118 structures of resolution of at least 2.0 Angstroms and R-factor no greater than 20%, a good quality model would be expected to have over 90% in the most favoured regions.

model1R89_01.ps

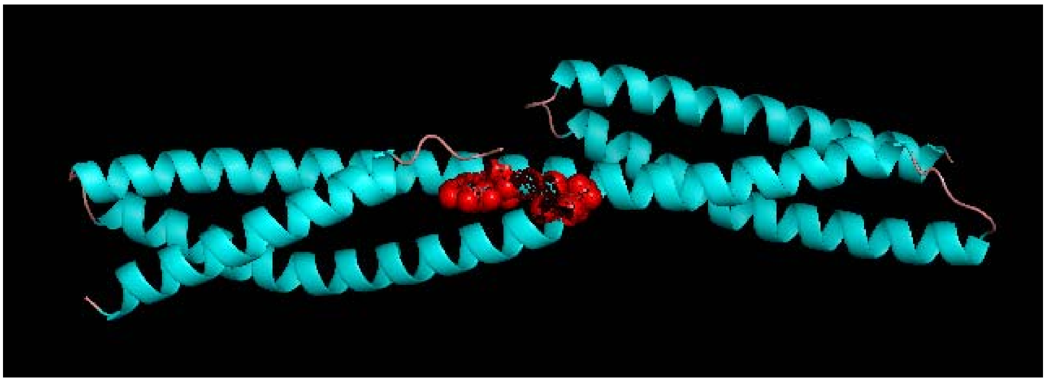

Residues in disallowed regions of the Ramachandran plot are in red

R9-10 model

WACWHELLSYLEKANKWLNEVEFKLKTTENIPGGAEEISEVLDSLENLMRHSEDNPNQIRILA
QTLTDGG-VMDELINEELETFNSRWRELHEEAVRRQKLLEQS
IQSAQETEKSLHLIQESLTFIDKQLAAYIADKVDAAQMPQEAQKIQSDLTSHEISLEEMKKHNQ
GKEAAQRVLSQIDVAQKKLQDVSMKFRL

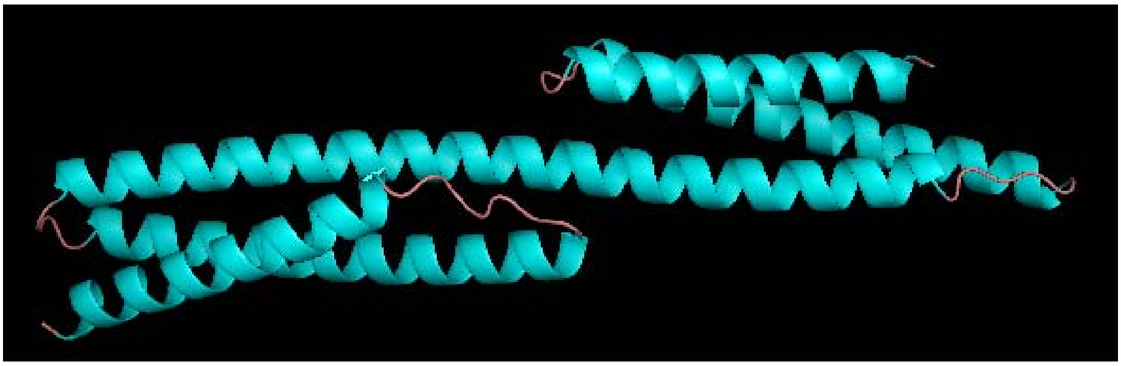

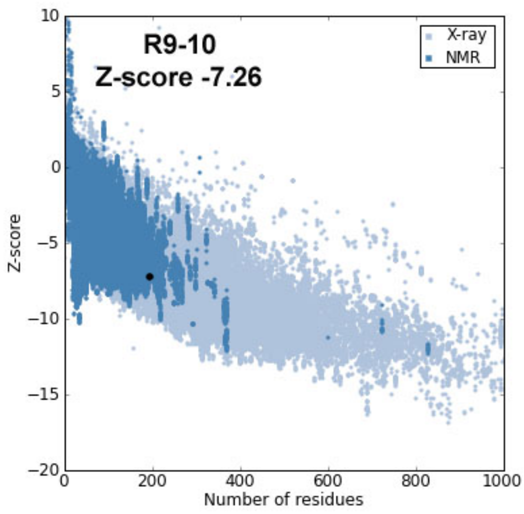

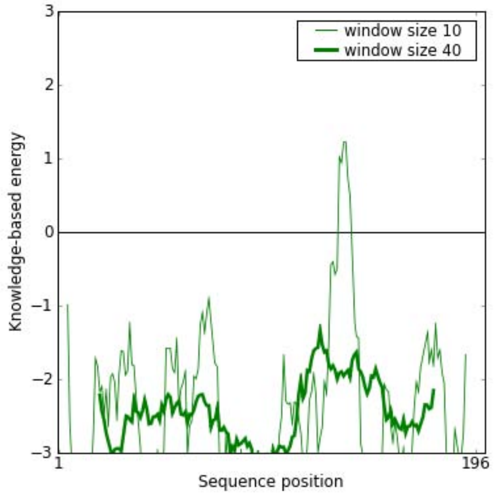

Verify3D structure evaluation

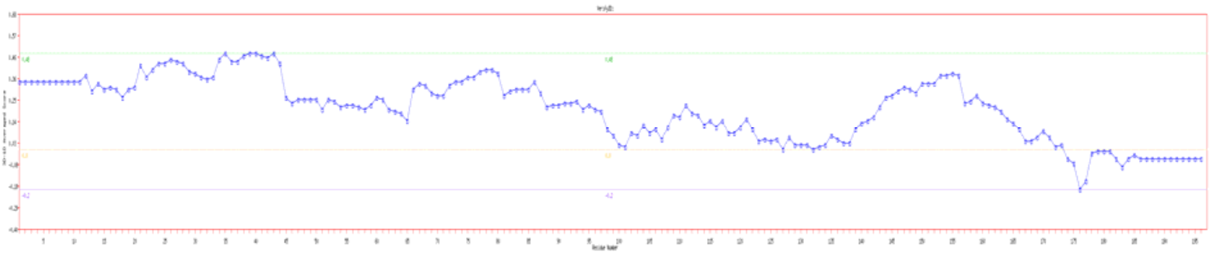

Fig S2

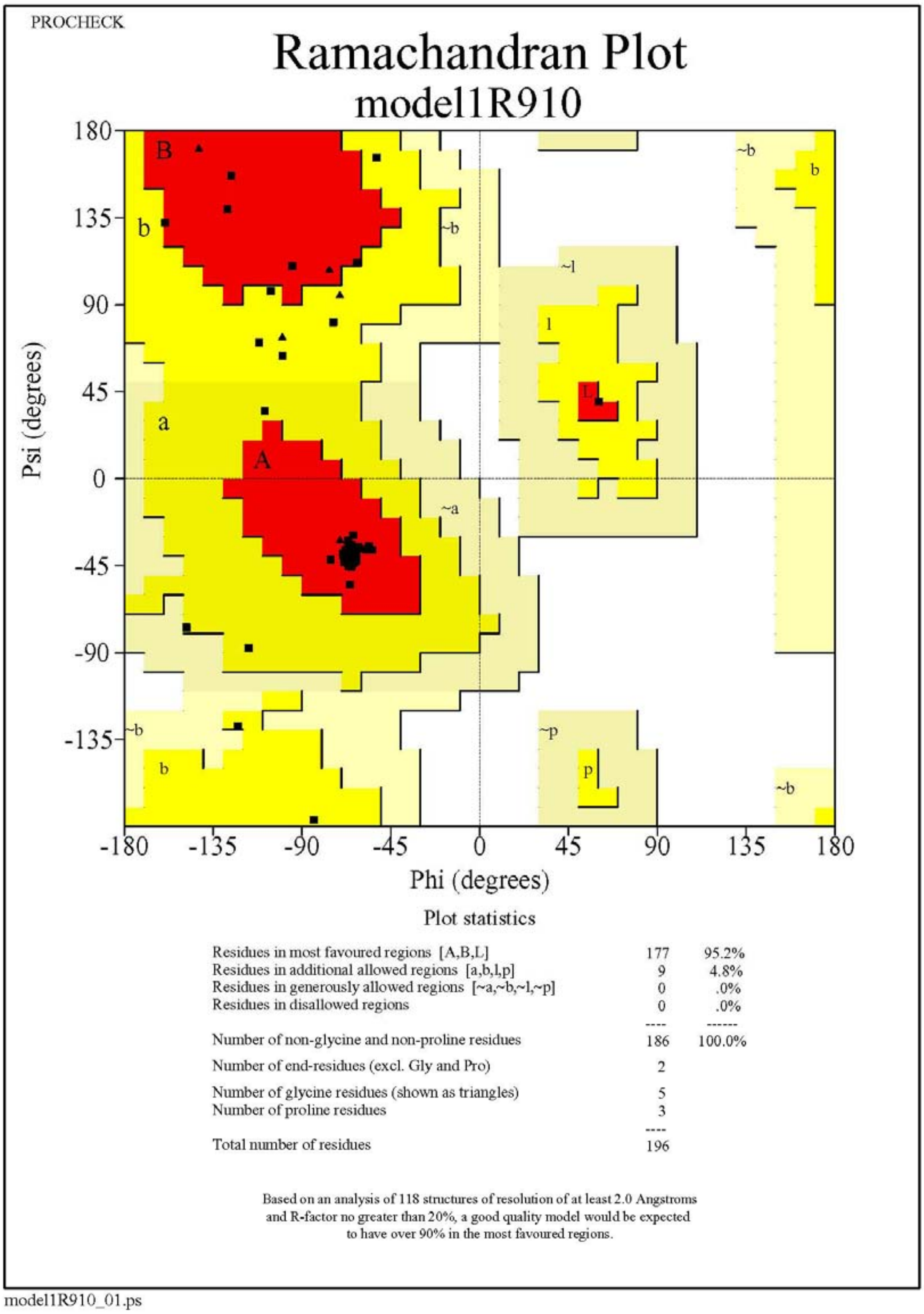

No residue in disallowed region

R10-11 model

IQSAQETEKSLHLIQESLTFIDKQLAAYIADKVDAAQMPQEAQKIQSDLTSHEISLEEMK
KHNQGKEAAQRVLSQIDVAQKKLQDVSMKFRLFQKPANFEQRLQESKMILDEVKMHLPAL
ETKSVEQEVVQSQLNHCVNLYKSLSEVKSEVEMVIKTGRQIVQKKQTENPKELDERVTAL
KLHYNELGAKVTERKQQLEKC

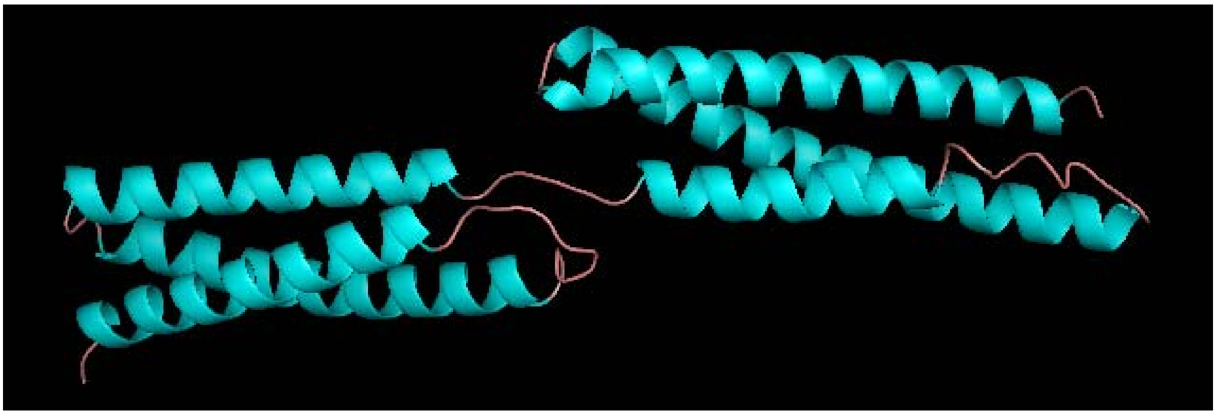

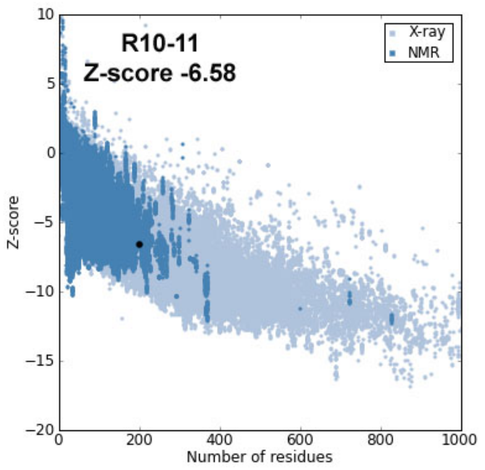

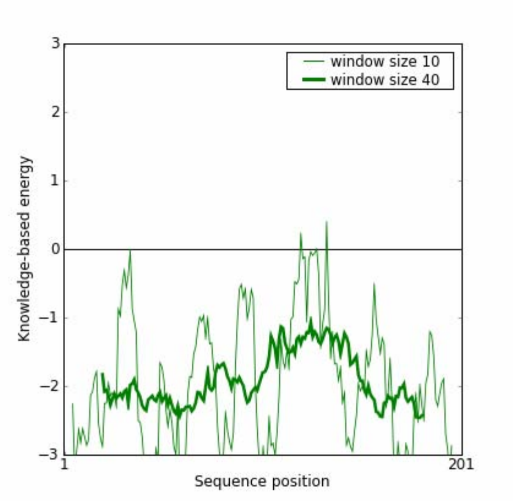

Verify3D structure evaluation

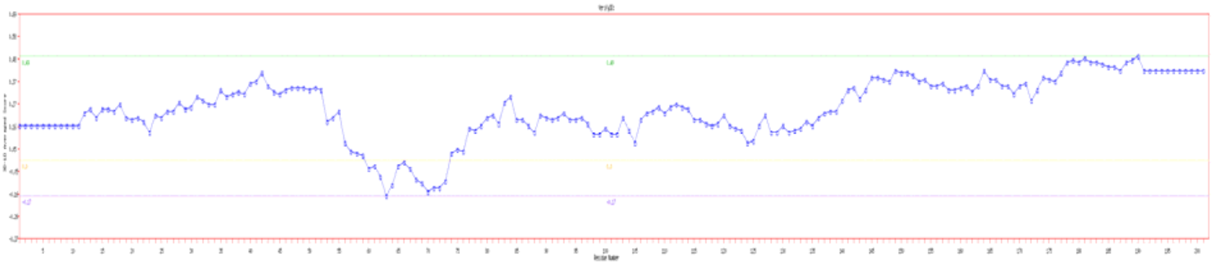

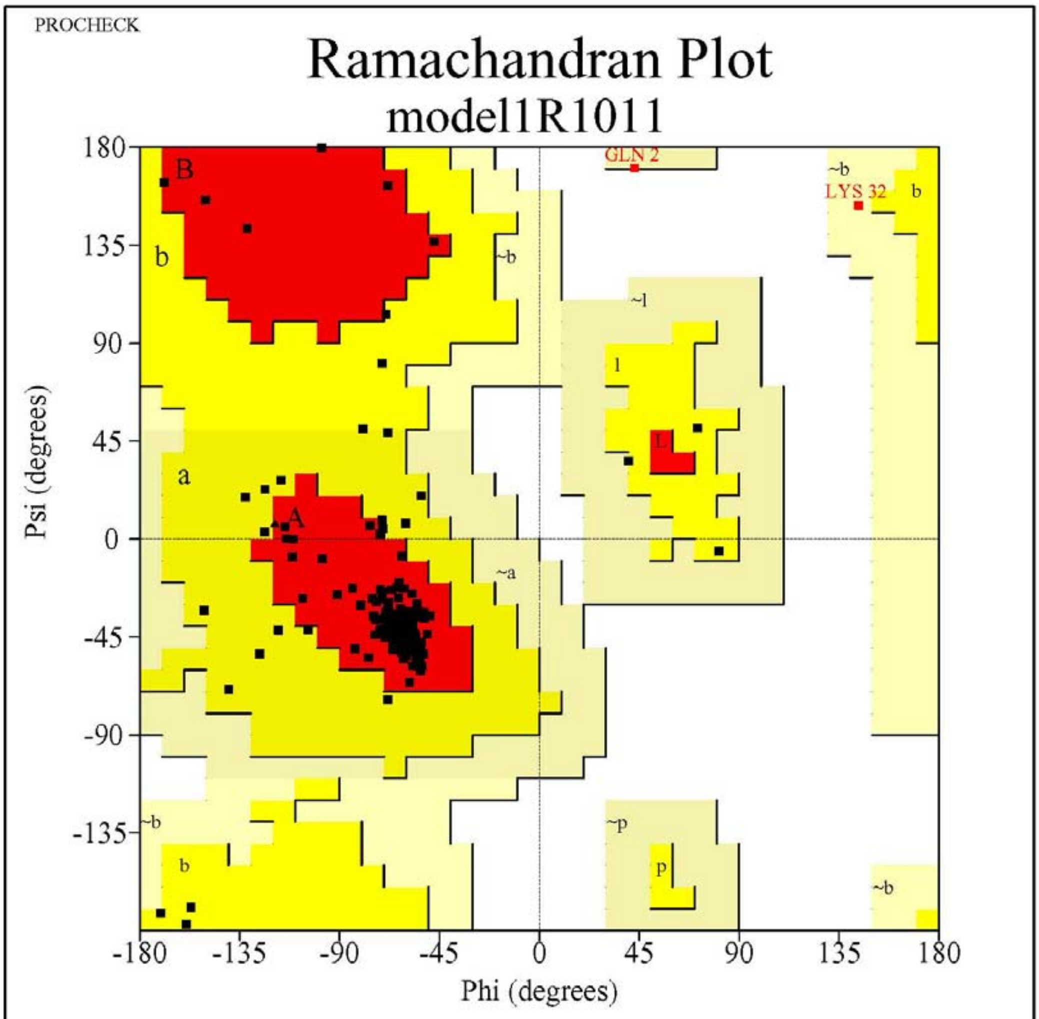

Plot statistics

Residues in most favoured regions [A,B,L]	168	87.5%
Residues in additional allowed regions [a,b,l,p]	22	11.5%
Residues in generously allowed regions [~a,~b,~l,~p]	2	1.0%
Residues in disallowed regions	0	.0%
	----	------
Number of non-glycine and non-proline residues	192	100.0%
Number of end-residues (excl. Gly and Pro)	2	
Number of glycine residues (shown as triangles)	3	
Number of proline residues	4	

Total number of residues	201	

Based on an analysis of 118 structures of resolution of at least 2.0 Angstroms and R-factor no greater than 20%, a good quality model would be expected to have over 90% in the most favoured regions.

model1R1011_01.ps

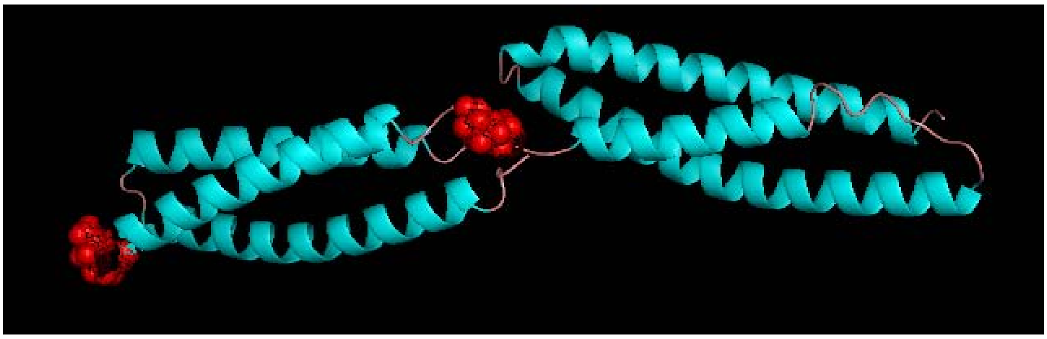

Residues in disallowed regions of the Ramachandran plotare in red

R11-12 model

FQKPANFEQRLQESKMILDEVKMHLPALETKSVEQEVVQSQLNHCVNLYKSLSEVKSEVEMV
IKTGRQIVQKKQTENPKELDERVTALKLHYNELGAKVTERKQQLEKC
LKLSRKMRKEMNVLTEWLAATDMELTKRSAVEGMPSNLDSEVAWGKATQKEIEKQKVHLK
SITEVGEALKTVLGKKETLVEDKLSLLNSNWIAVTSRAEEWLNLLLEY

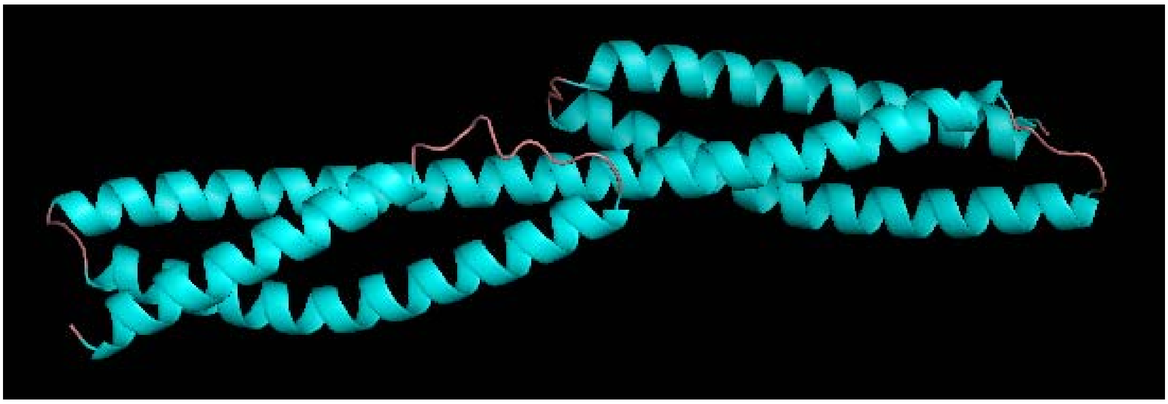

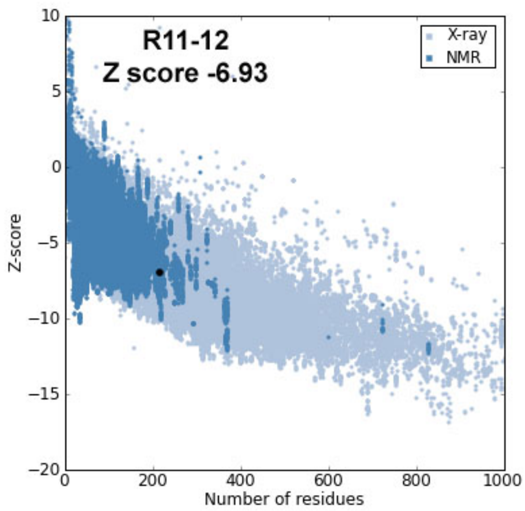

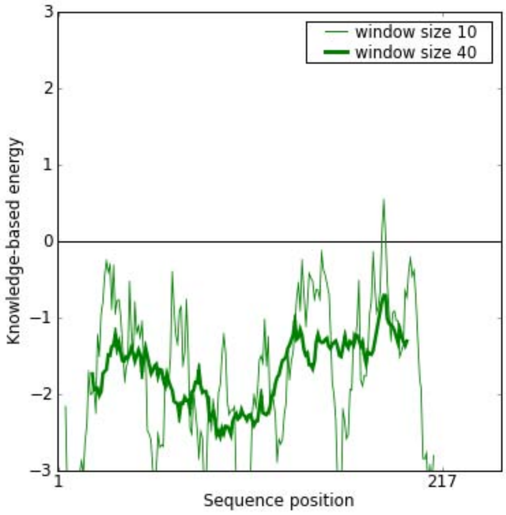

Verify3D structure evaluation

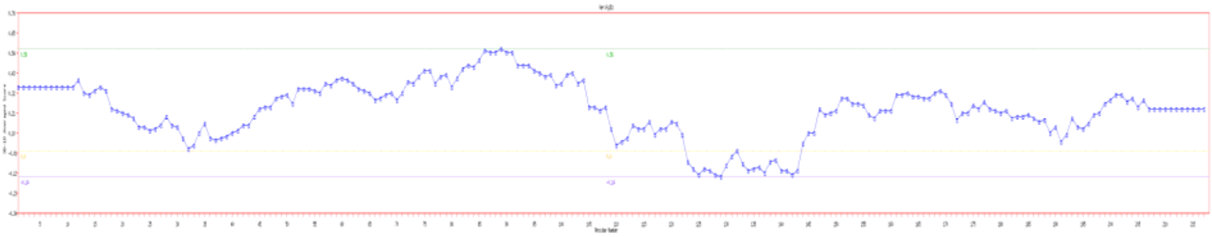

PROCHECK

Ramachandran Plot
model1R1112

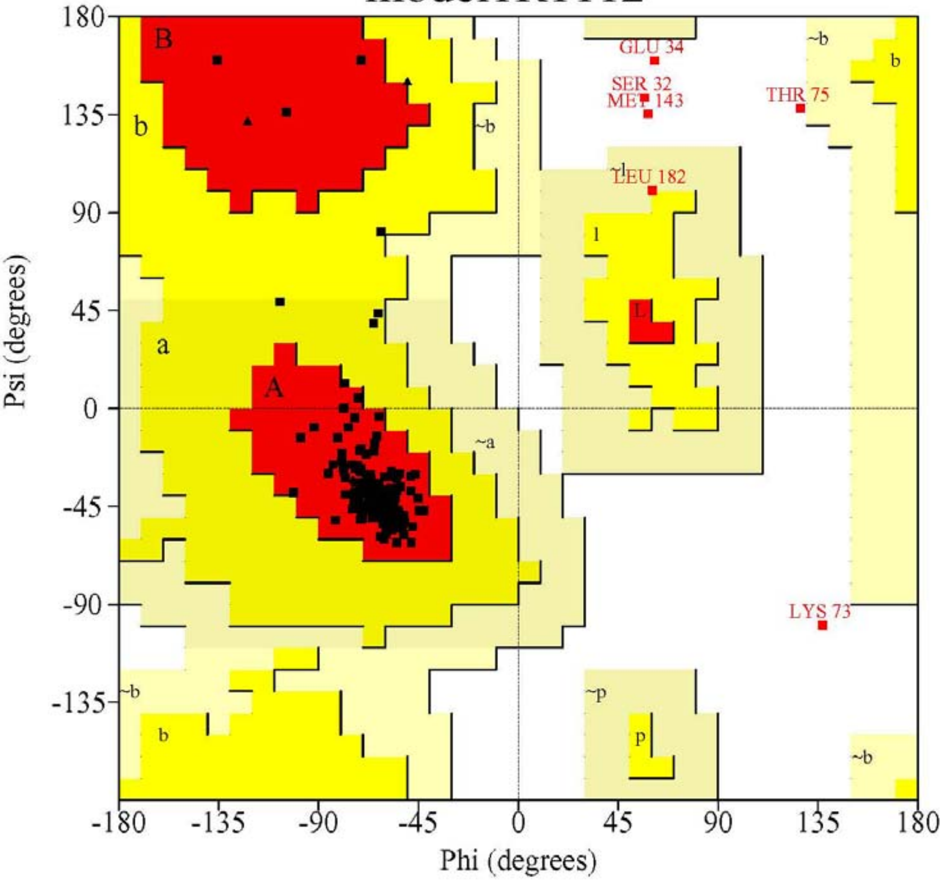

Plot statistics

Residues in most favoured regions [A,B,L]	195	95.1%
Residues in additional allowed regions [a,b,l,p]	4	2.0%
Residues in generously allowed regions [~a,~b,~l,~p]	1	.5%
Residues in disallowed regions	5	2.4%
	----	------
Number of non-glycine and non-proline residues	205	100.0%
Number of end-residues (excl. Gly and Pro)	2	
Number of glycine residues (shown as triangles)	6	
Number of proline residues	4	

Total number of residues	217	

Based on an analysis of 118 structures of resolution of at least 2.0 Angstroms and R-factor no greater than 20%, a good quality model would be expected to have over 90% in the most favoured regions.

model1R1112_01.ps

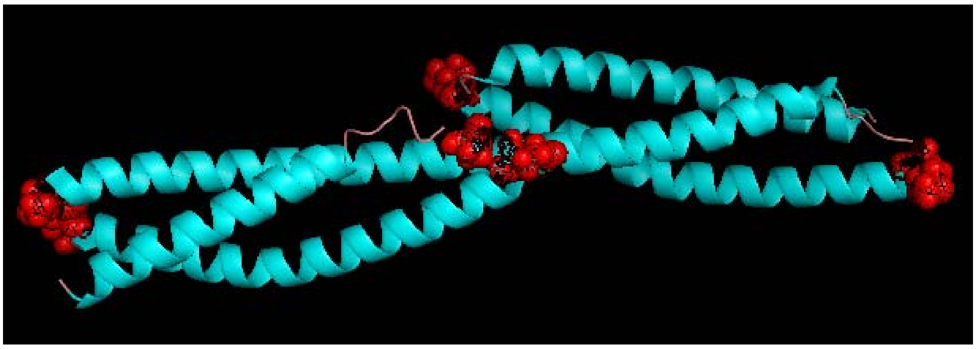

Residues in disallowed regions of the Ramachandran plot are in red

R12-13 model

LKLSRKMRKEMNVLTEWLAATDMELTKRSAVEGMPSNLDSEVAWGKATQKEIEKQKVHLK
SITEVGEALKTVLGKKETLVEDKLSLLNSNWIAVTSRAEEWLNLLLEYQKHMETFDQNVD
HITKWIIQADTLLDESEKKKPQQKEDVLKRLKAELNDIRPKVDSTRDQAANLMANRGDHC
RKLVEPQISELNHRFAAISHRIKTGKASIPLK

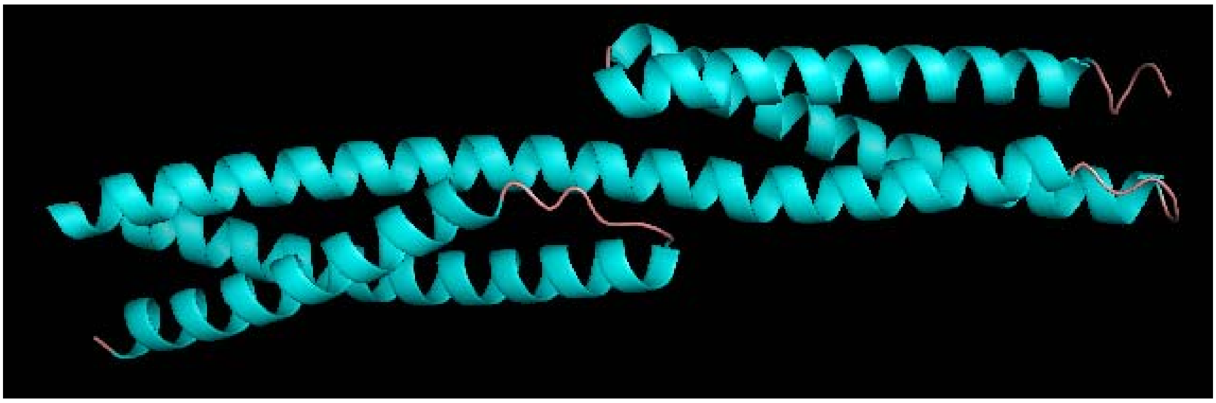

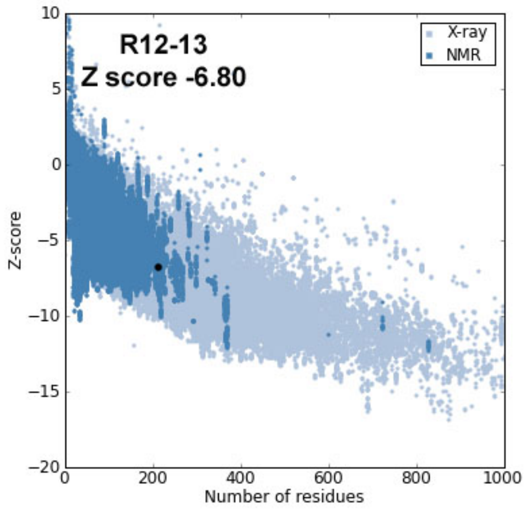

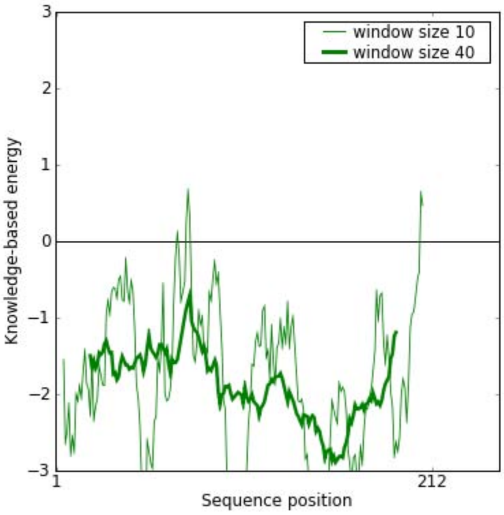

Verify3D structure evaluation

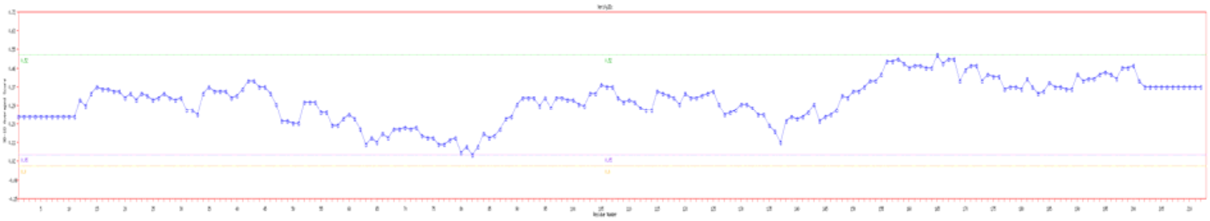

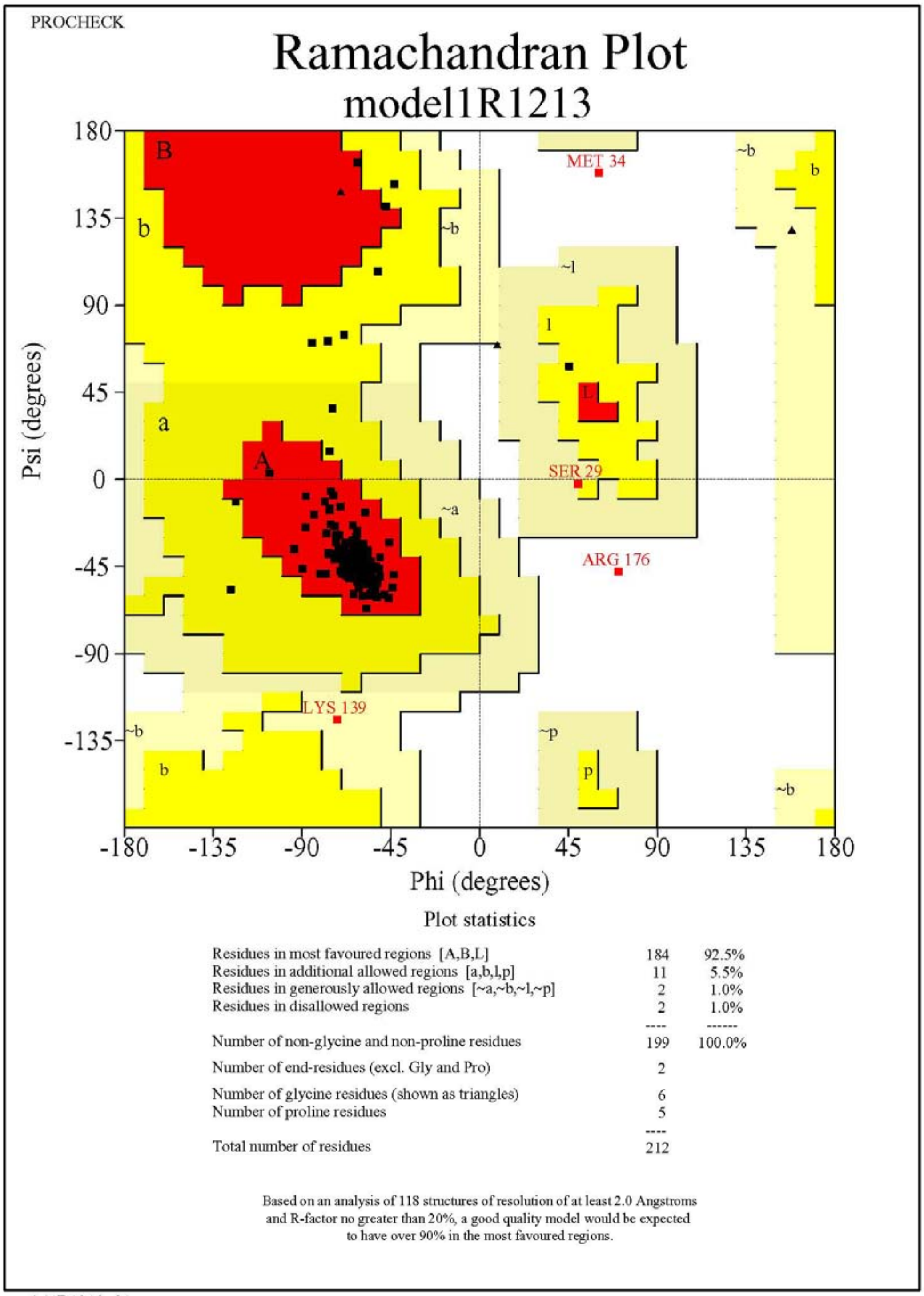

model1R1213_01.ps

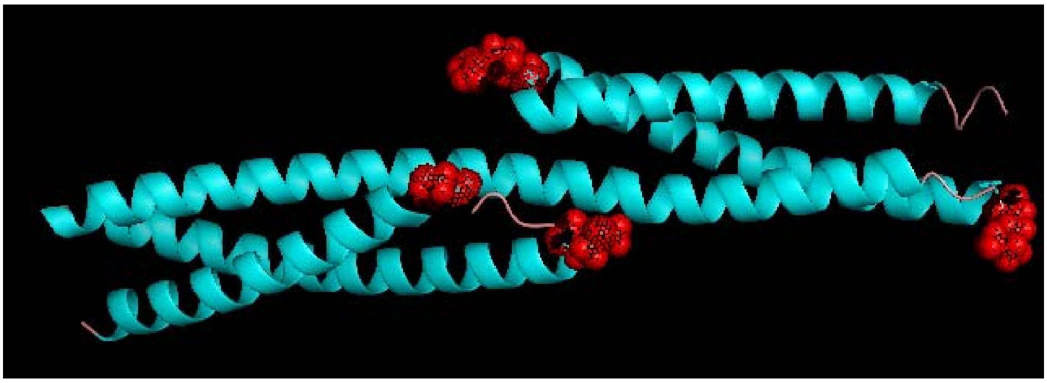

Residues in disallowed regions of the Ramachandran plot are in red

Fig S2

R13-14 model

QKHMETFDQNVDHITKWIIQADTLLDESEKKKPQQKEDVLKRLKAELNDIRPKVDSTRDQ
AANLMANRGDHCRKLVEPQISELNHRFAAISHRIKTGKASIPLK
ELEQFNSDIQKLLEPLEAEIQQGVNLKEEDFNKDMNEDNEGTVKELLQRGDNLQQRITDERK
REEIKIKQQLLQTKHNALKDLRSQRRKKALEI

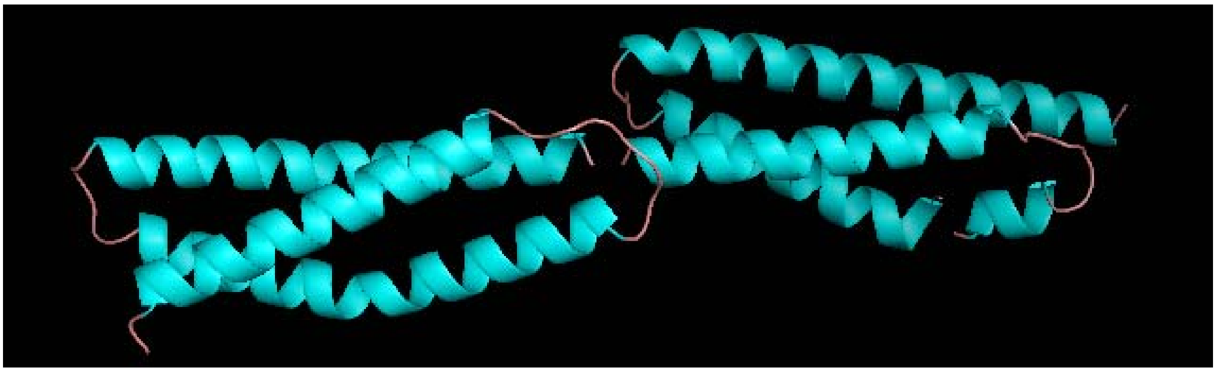

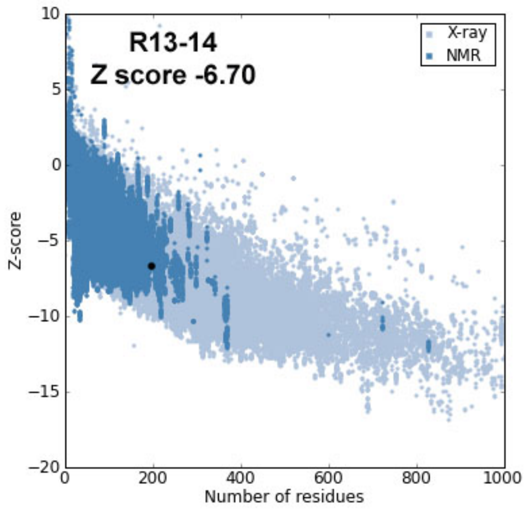

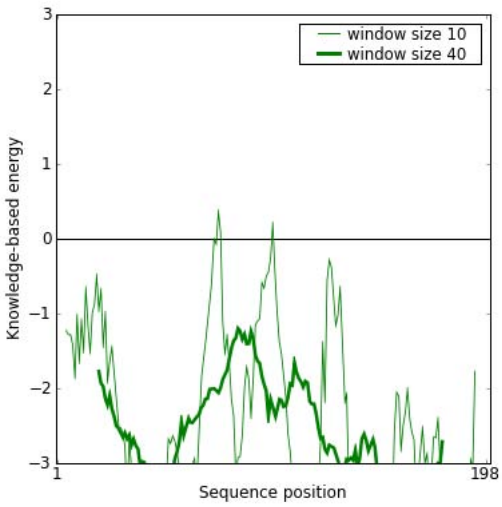

Verify3D structure evaluation

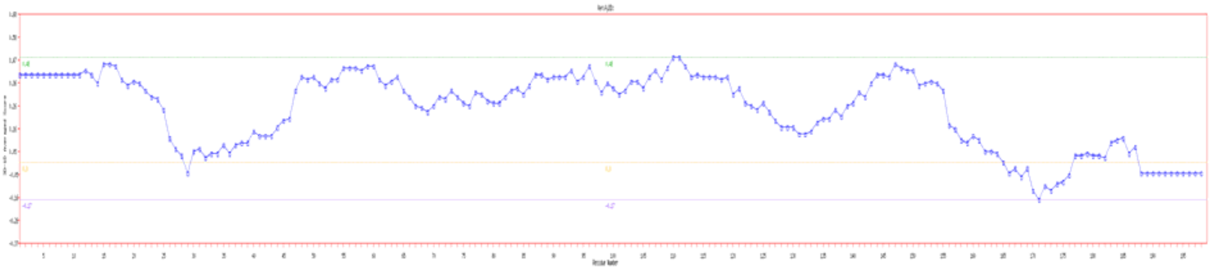

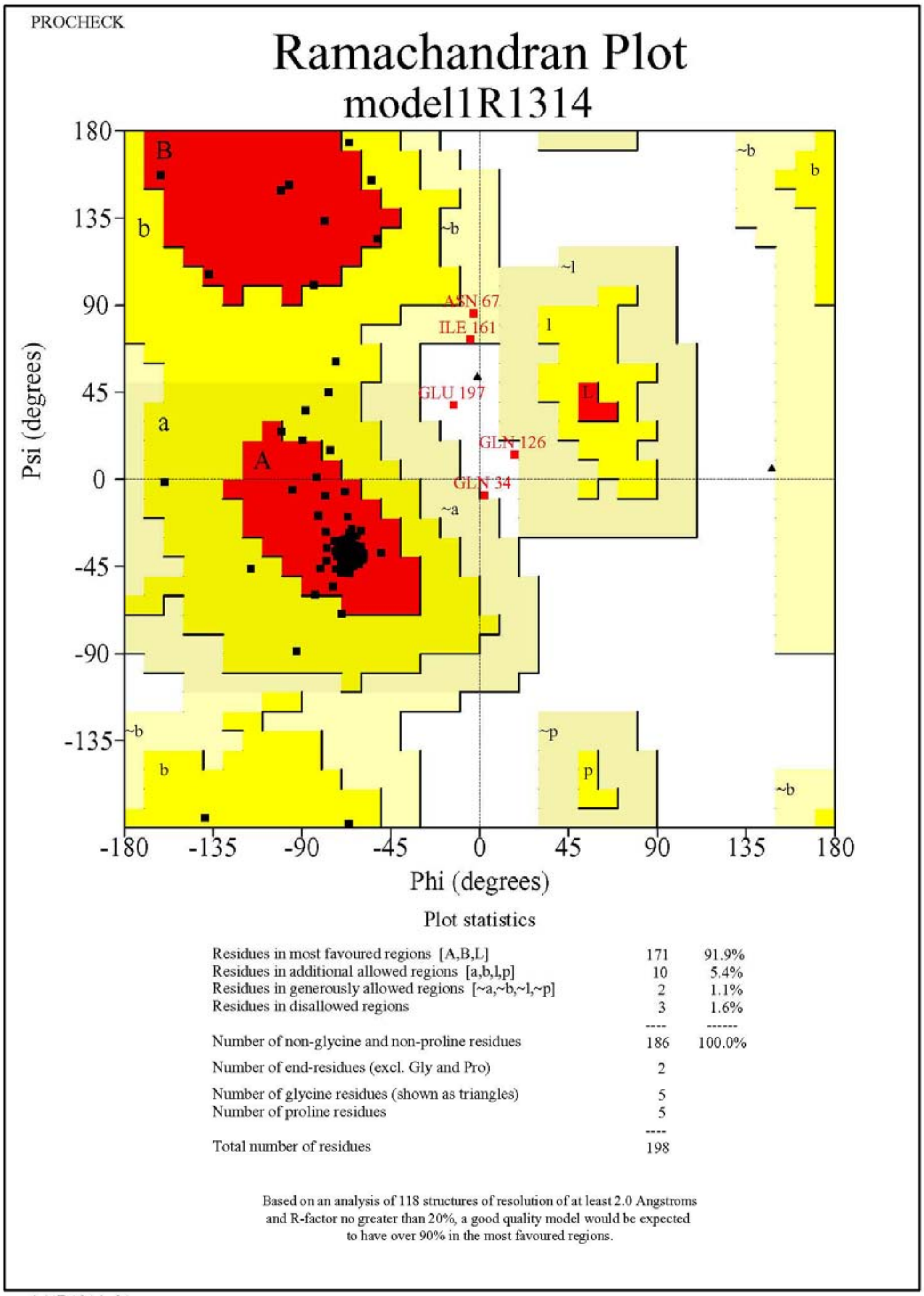

model1R1314_01.ps

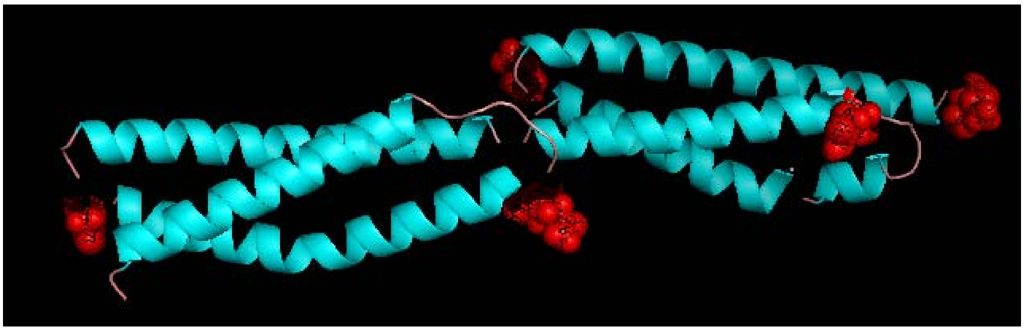

Residues in disallowed regions of the Ramachandran plot are in red

Fig S2

R14-15 model

ELEQFNSDIQKLLEPLEAEIQQGVNLKEEDFNKDMNEDNEGTVKELLQRGDNLQQRITDERK
REEIKIKQQLLQTKHNALKDLRSQRRKKALEISHQWY
QYKRQADDLLKCLDDIEKKLASLPEPRDERKIKEIDRELQKKKEELNAVRRQAEGLSEDGAA
MAVEPTQIQLSKRWREIESKFAQFRRLNFAQ

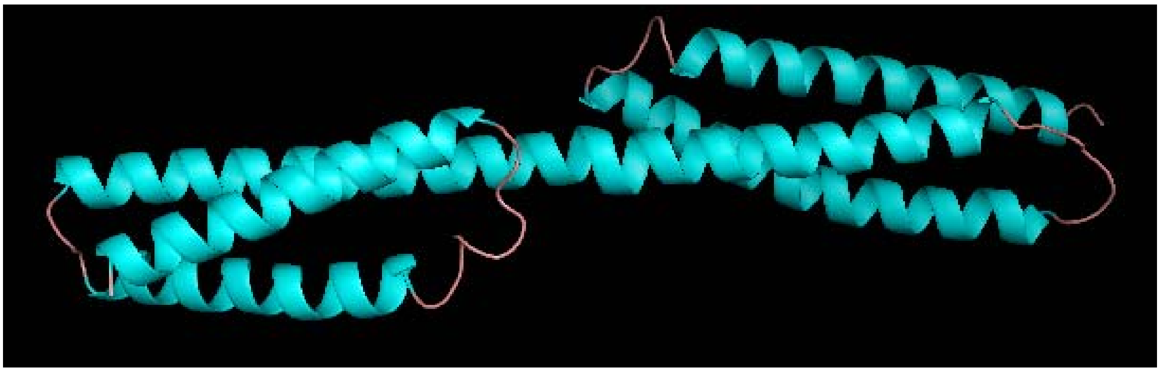

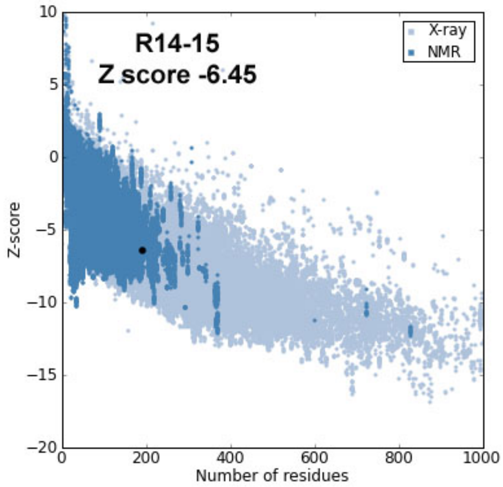

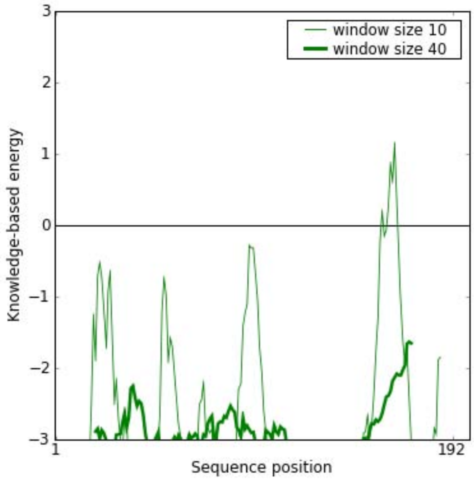

Verify3D structure evaluation

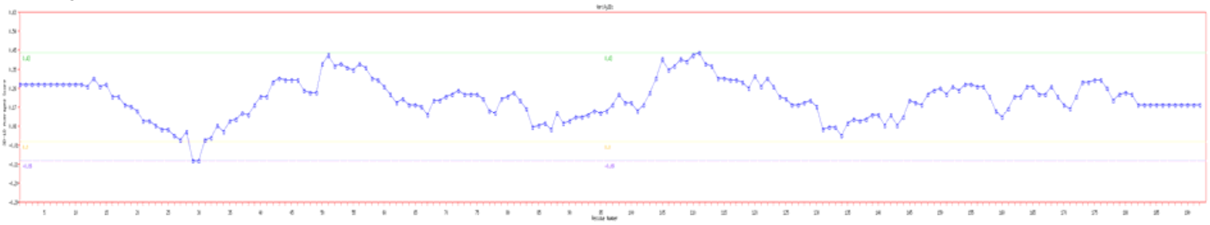

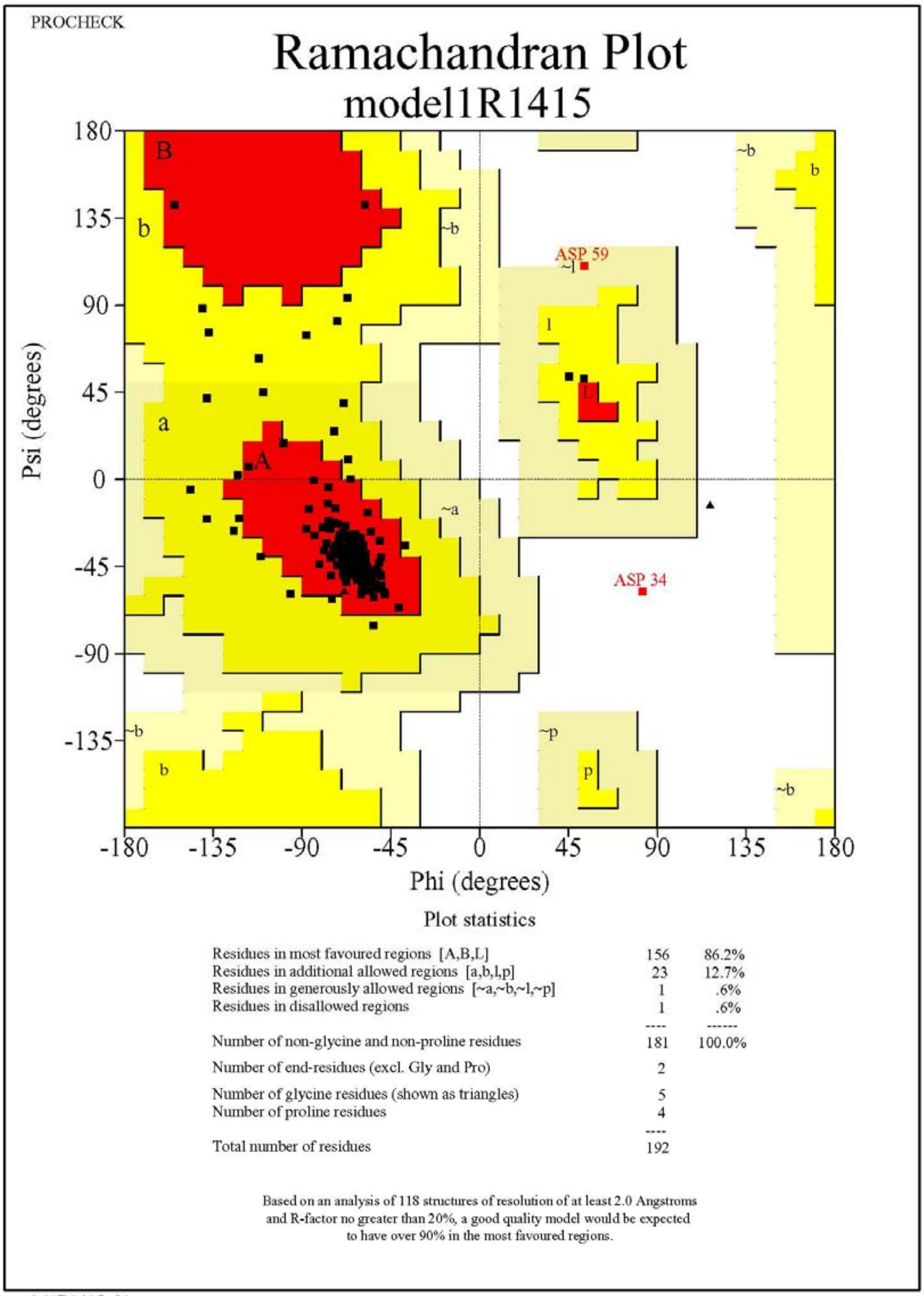

model1R1415_01.ps

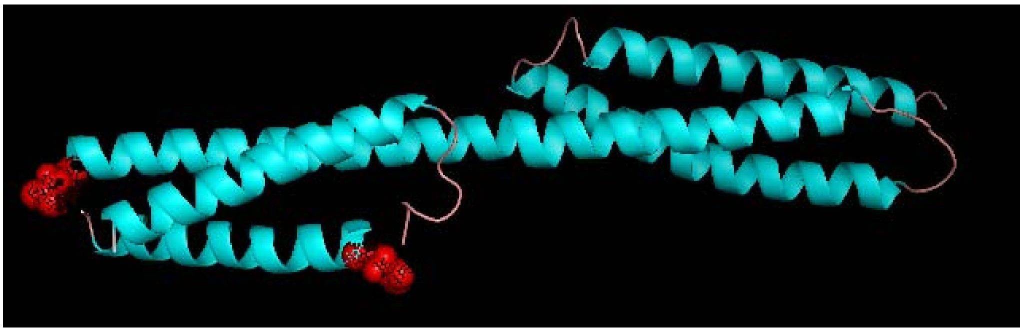

Residues in disallowed regions of the Ramachandran plot are in red

R15-16 model

SHQWYQYKRQADDLLKCLDDIEKKLASLPEPRDERKIKEIDRELQKKKEELNAVRRQAEGLS
EDGAAMAVEPTQIQLSKRWREIESKFAQFRRLNFAQI
HTVREETMMVMTEDMPLEISYVPSTYLTEITHVSQALLEVEQLLNAPDLCAKDFEDLFKQEES
LKNIKDSLQQSSGRIDIIHSKKTAALQSATPVERVK
LQEALSQLDFQWEKVNKMYKDRQGRFDRS

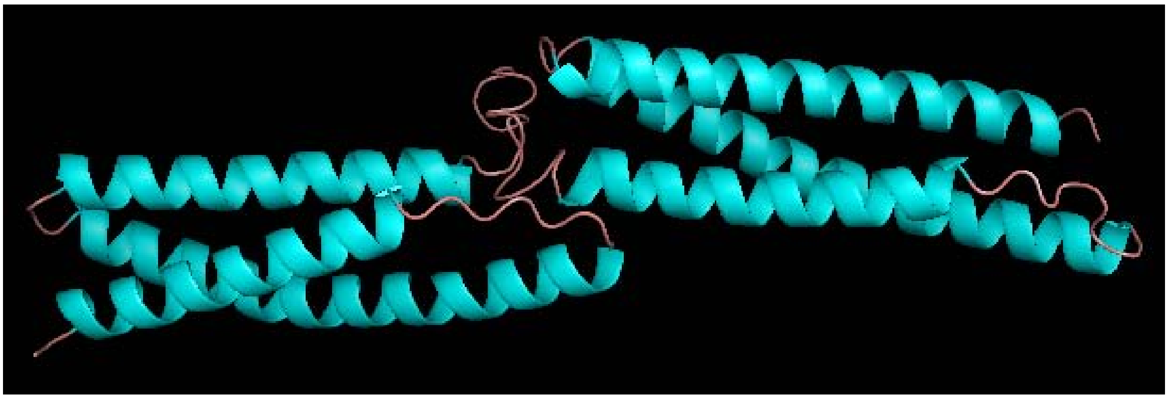

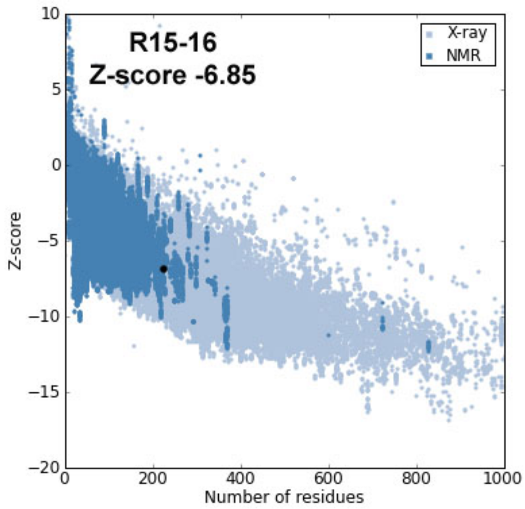

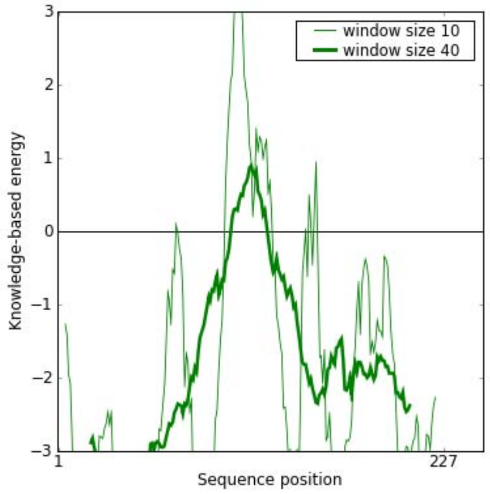

Verify3D structure evaluation

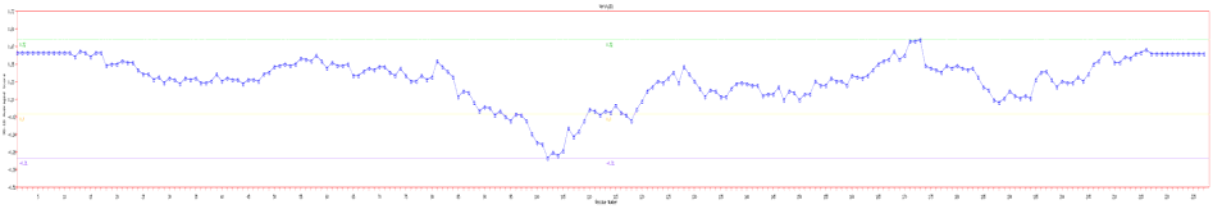

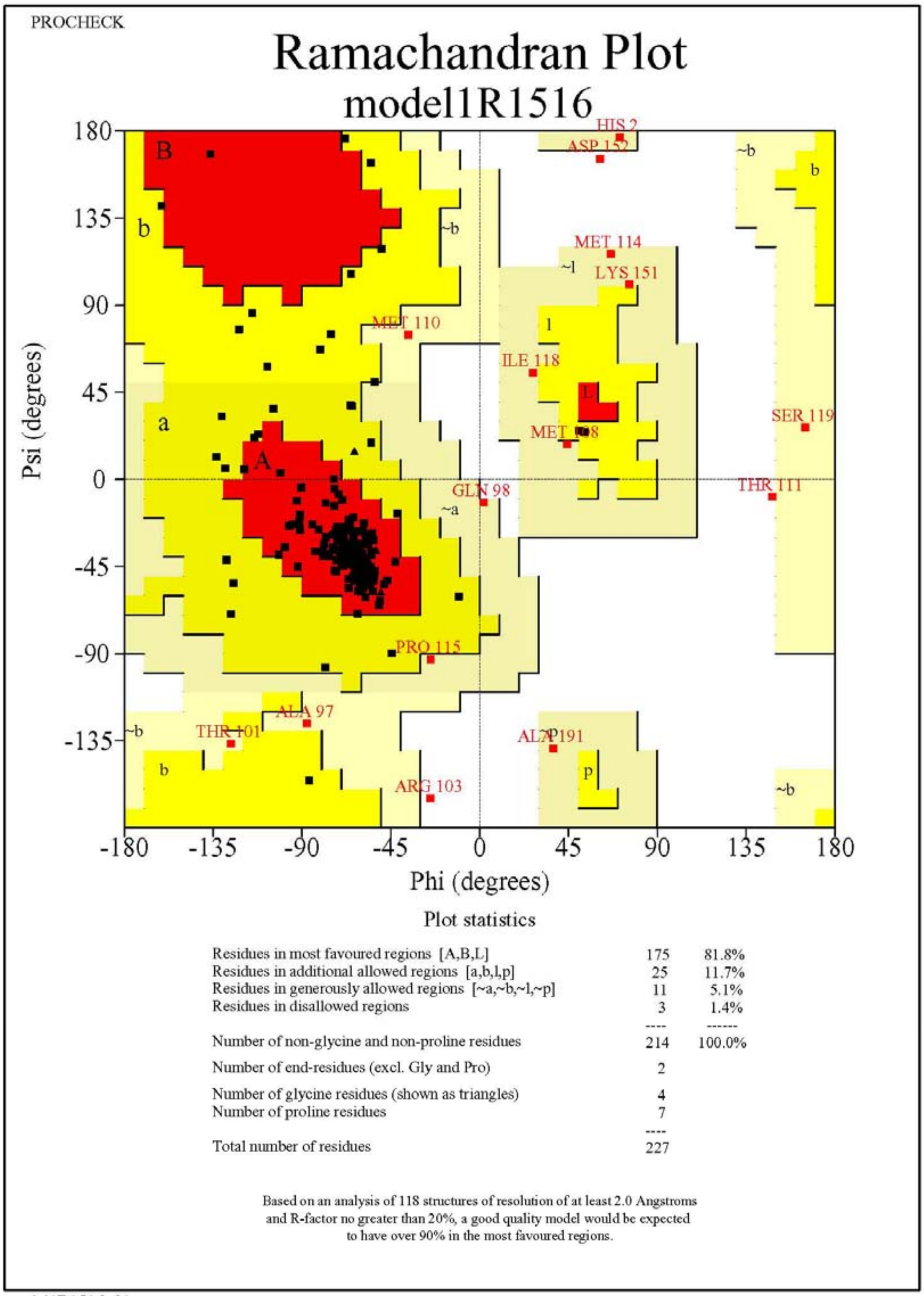

model1R1516_01.ps

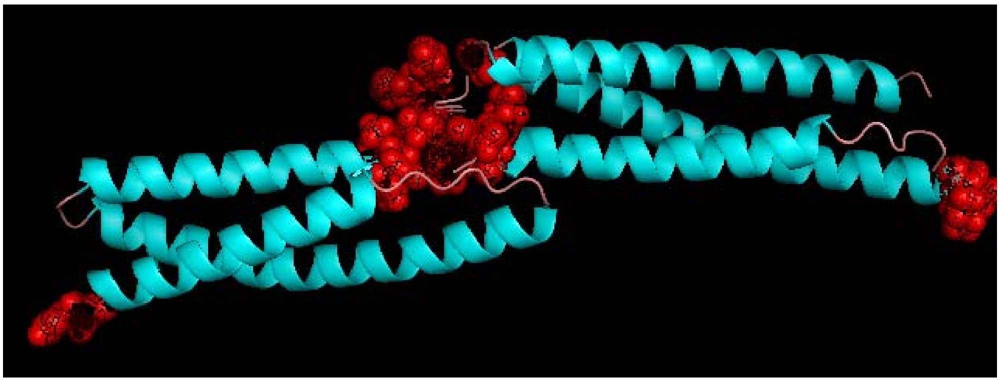

Residues in disallowed regions of the Ramachandran plot are in red

R16-17 model

SYVPSTYLTEITHVSQALLEVEQLLNAPDLCAKDFEDLFKQEESLKNIKDSLQQSSGRID
IIHSKKTAALQSATPVERVKLQEALSQLDFQWEKVNKMYKDRQGRFDRSVEKWRRFHYDI
KIFNQWLTEAEQFLRKTQIPENWEHAKYKWYLKELQDGIGQRQTVVRTLNATGEEIIQQS
SKTDASILQEKLGSLNLRWQEVCKQLSDRKKRLEEQKNILSEFQ

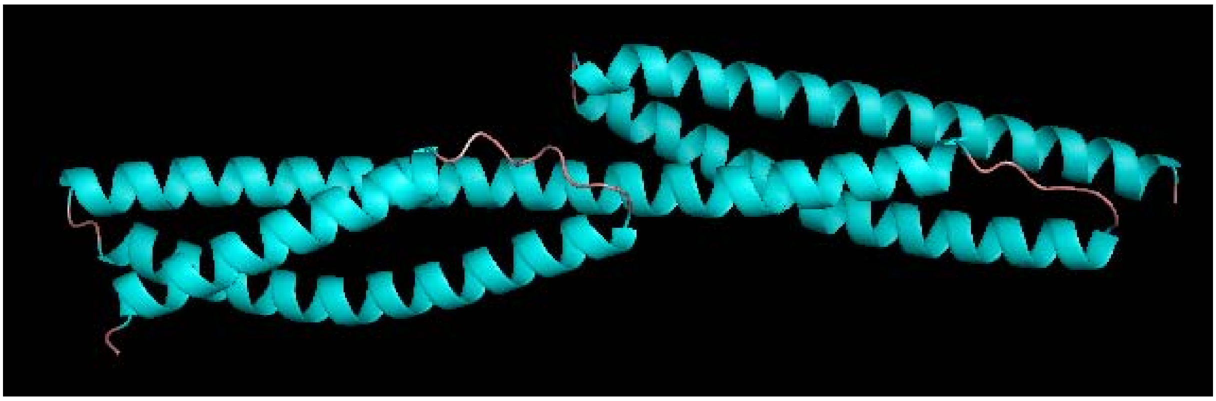

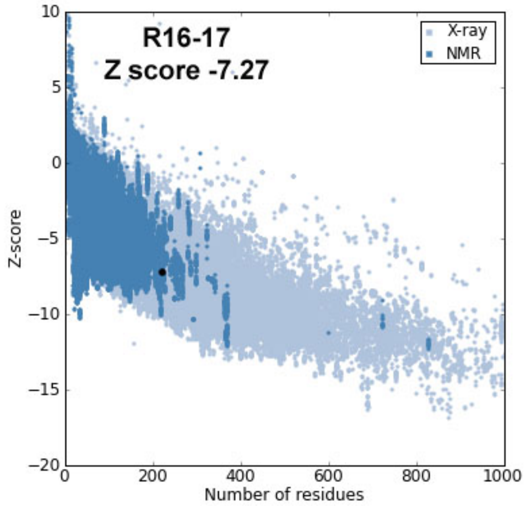

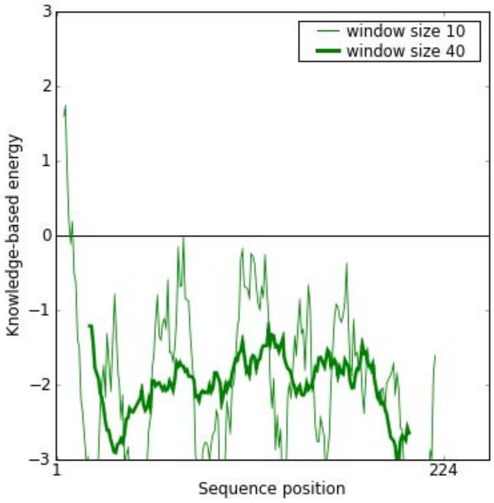

Verify3D structure evaluation

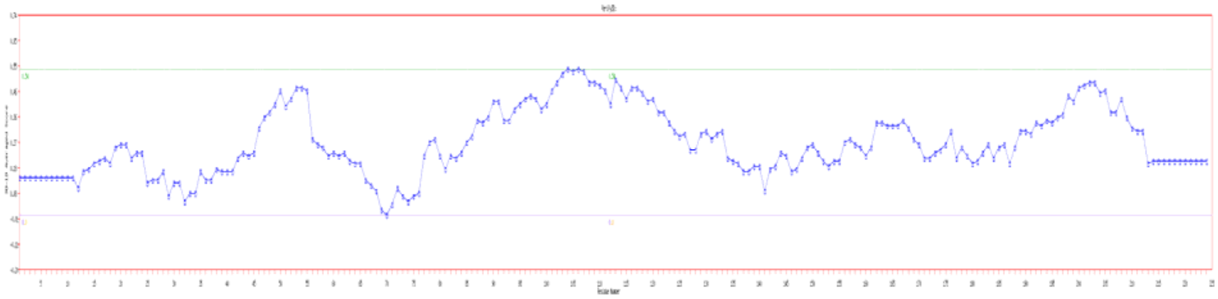

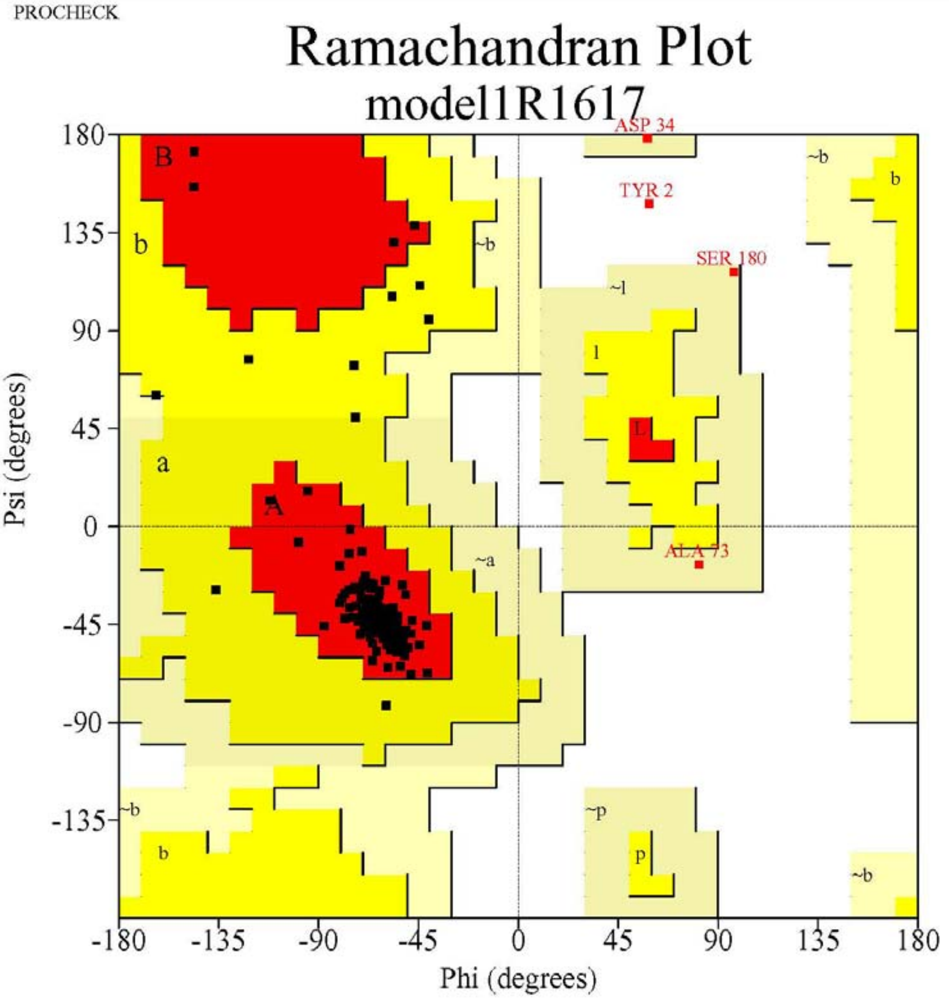

Plot statistics

Residues in most favoured regions [A,B,L]	199	93.9%
Residues in additional allowed regions [a,b,l,p]	9	4.2%
Residues in generously allowed regions [~a,~b,~l,~p]	3	1.4%
Residues in disallowed regions	1	.5%
	----	------
Number of non-glycine and non-proline residues	212	100.0%
Number of end-residues (excl. Gly and Pro)	2	
Number of glycine residues (shown as triangles)	6	
Number of proline residues	4	

Total number of residues	224	

Based on an analysis of 118 structures of resolution of at least 2.0 Angstroms and R-factor no greater than 20%, a good quality model would be expected to have over 90% in the most favoured regions.

model1R1617_01.ps

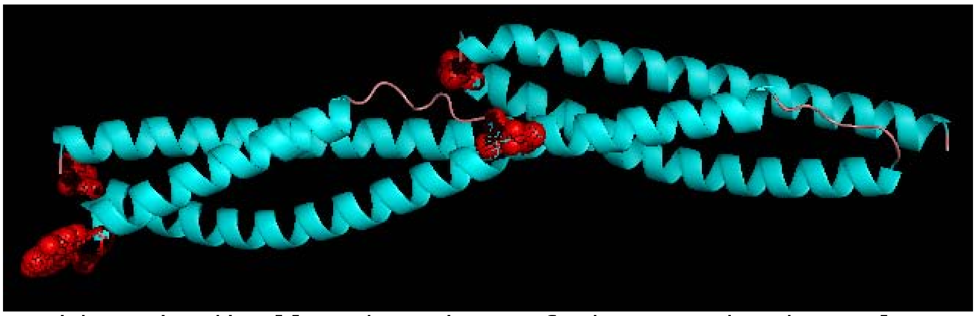

Residues in disallowed regions of the Ramachandran plot are in red

R17-18 model

VEKWRRFHYDIKIFNQWLTEAEQFLRKTQIPENWEHAKYKWYLKELQDGIGQRQTVVRTLN
ATGEEIIQQSSKTDASILQEKLGSLNLRWQEVCKQLSD
RKKRLEEQKNILSEFQRDLNEFVLWLEEADNIASIPLEPGKEQQLKEKLEQVKLLVEELPLRQG
ILKQLNETGGPVLVSAPISPEEQDKLENKLKQTNL
QWIKVSRALPEKQGEIEAQ

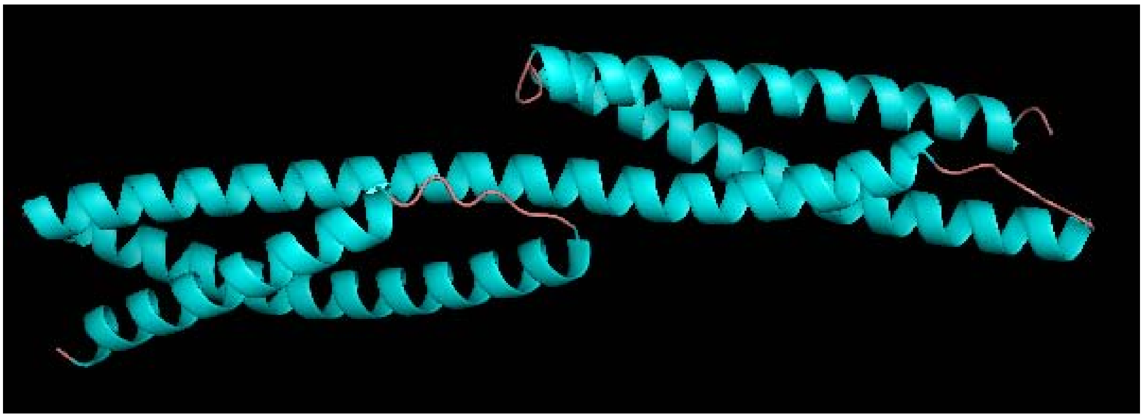

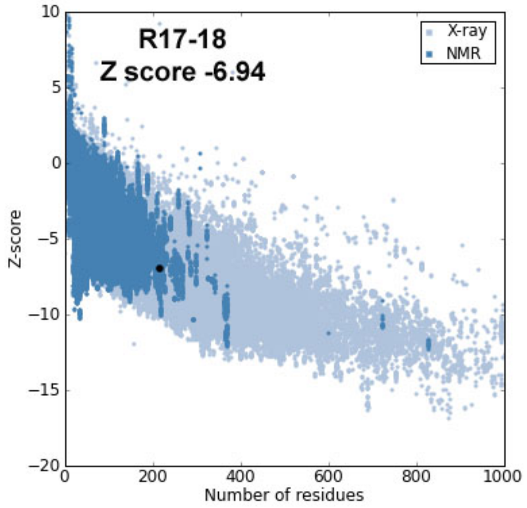

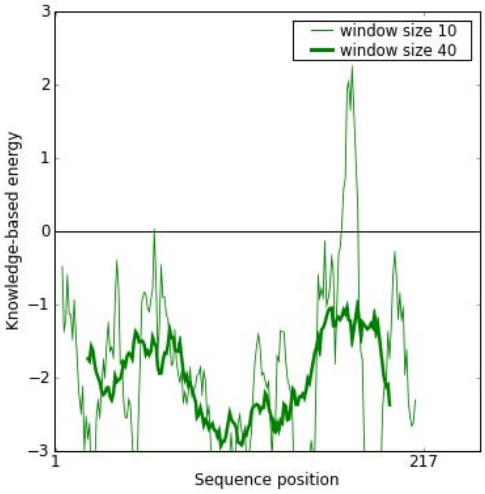

Verify3D structure evaluation

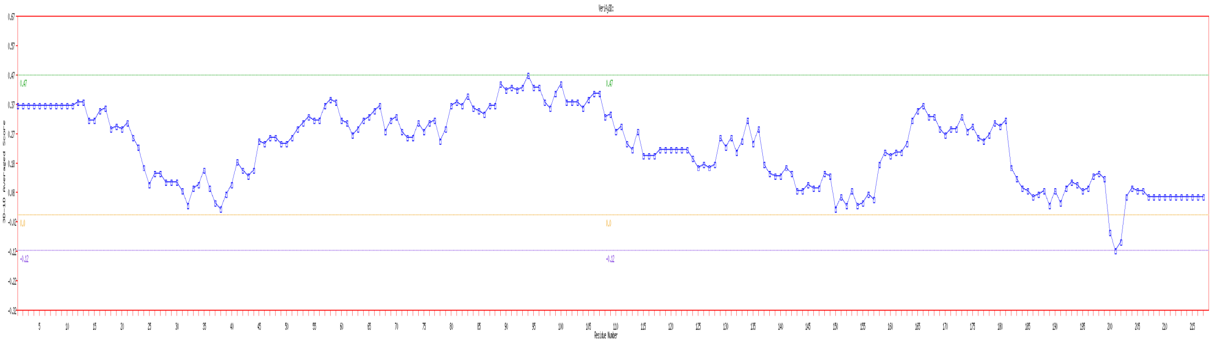

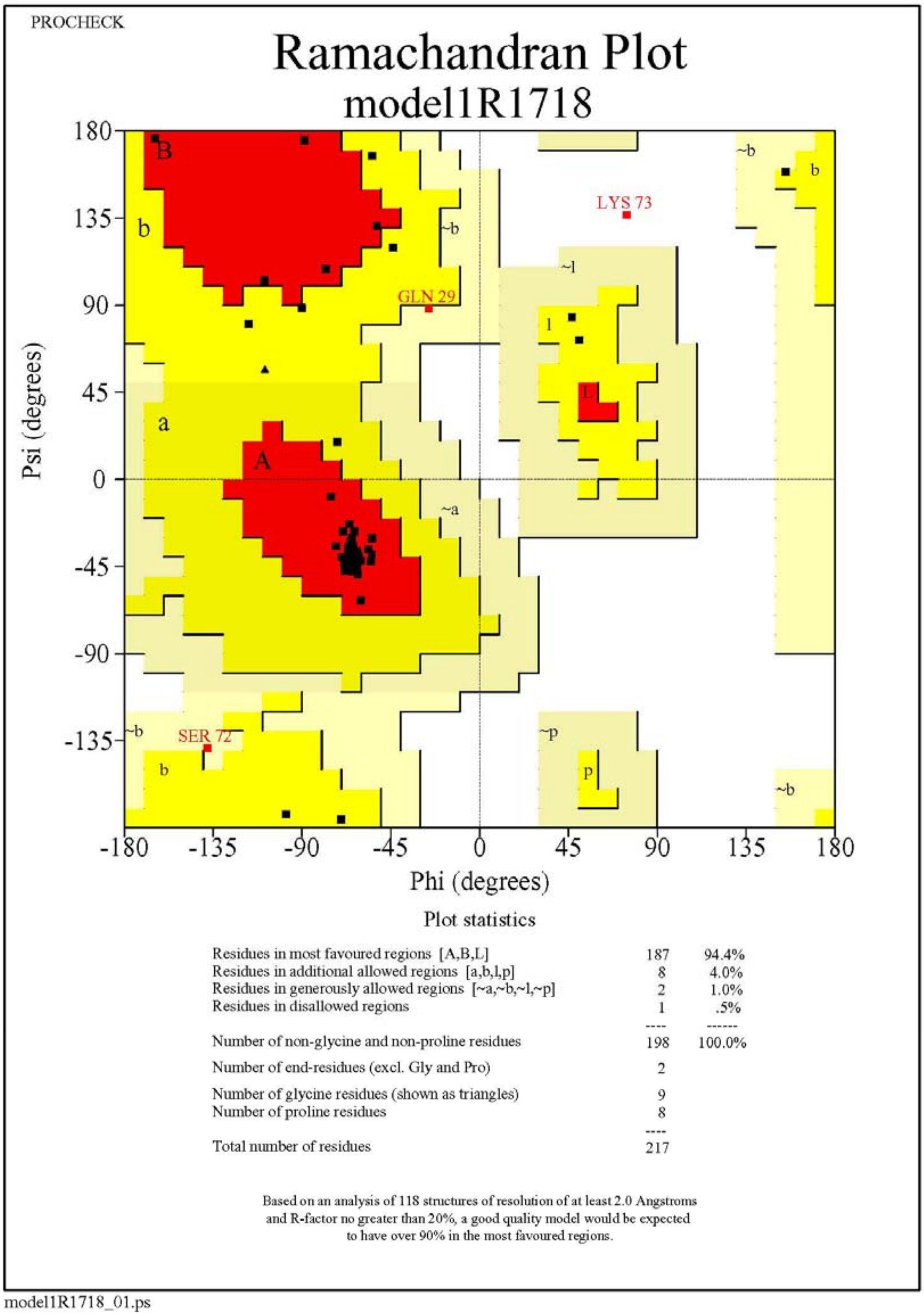

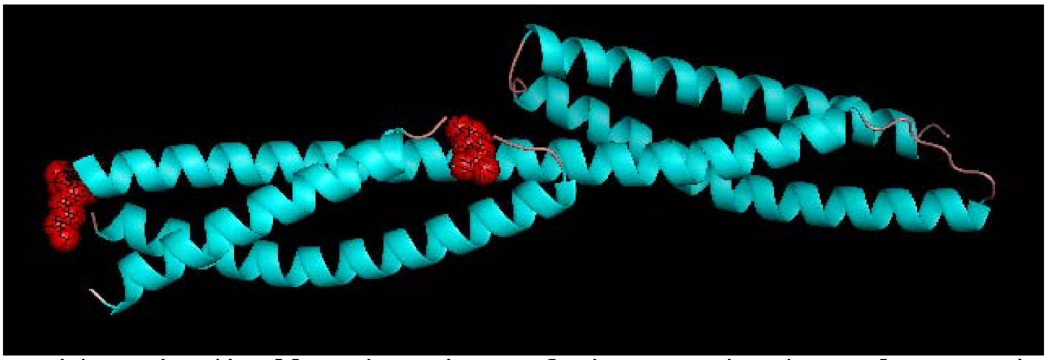

Residues in disallowed regions of the Ramachandran plot are in red

R18-19 model

KNILSEFQRDLNEFVLWLEEADNIASIPLEPGKEQQLKEKLEQVKLLVEELPLRQGILKQLNET
GGPVLVSAPISPEEQDKLENKLKQTNLQWIKVSRALPEKQGEIEAQIKDLGQLEKKLEDLEEQ
LNHLLLWLSPIRNQLEIYNQPNQEGPFDVQETEIAVQAKQPDVEEILSKGQHLYKEKPATQPV
KRKLEDLSSEWKAVNRLLQELRAKQPDL

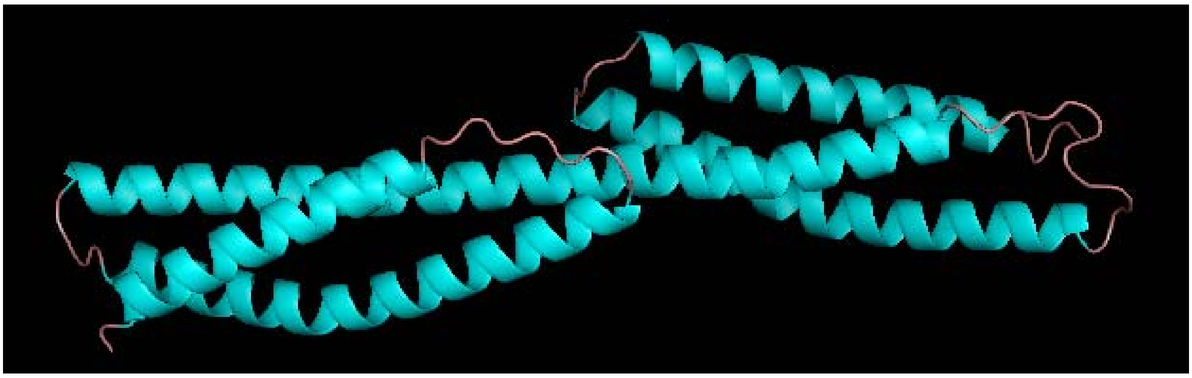

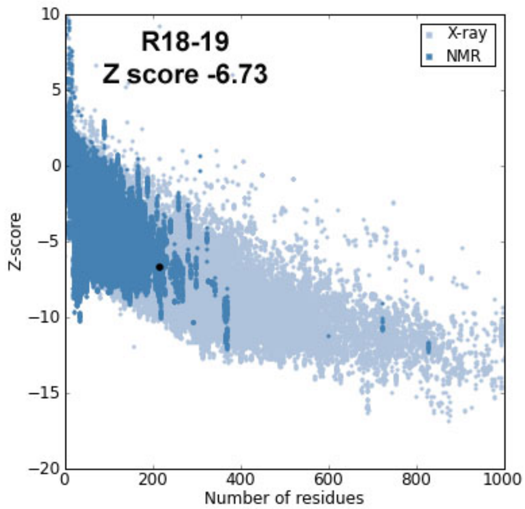

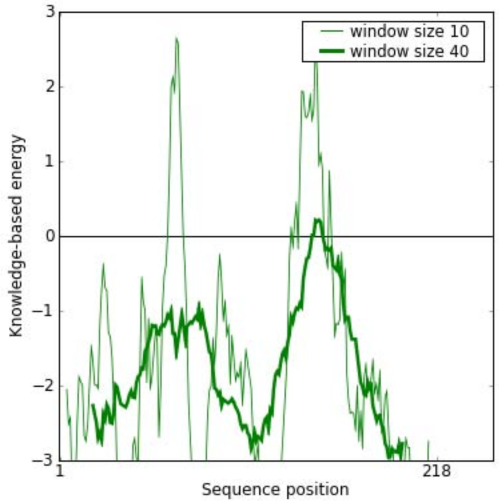

Verify3D structure evaluation

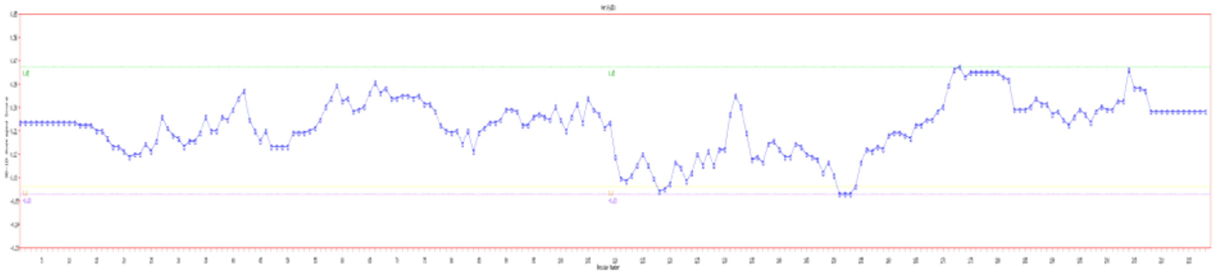

Fig S2

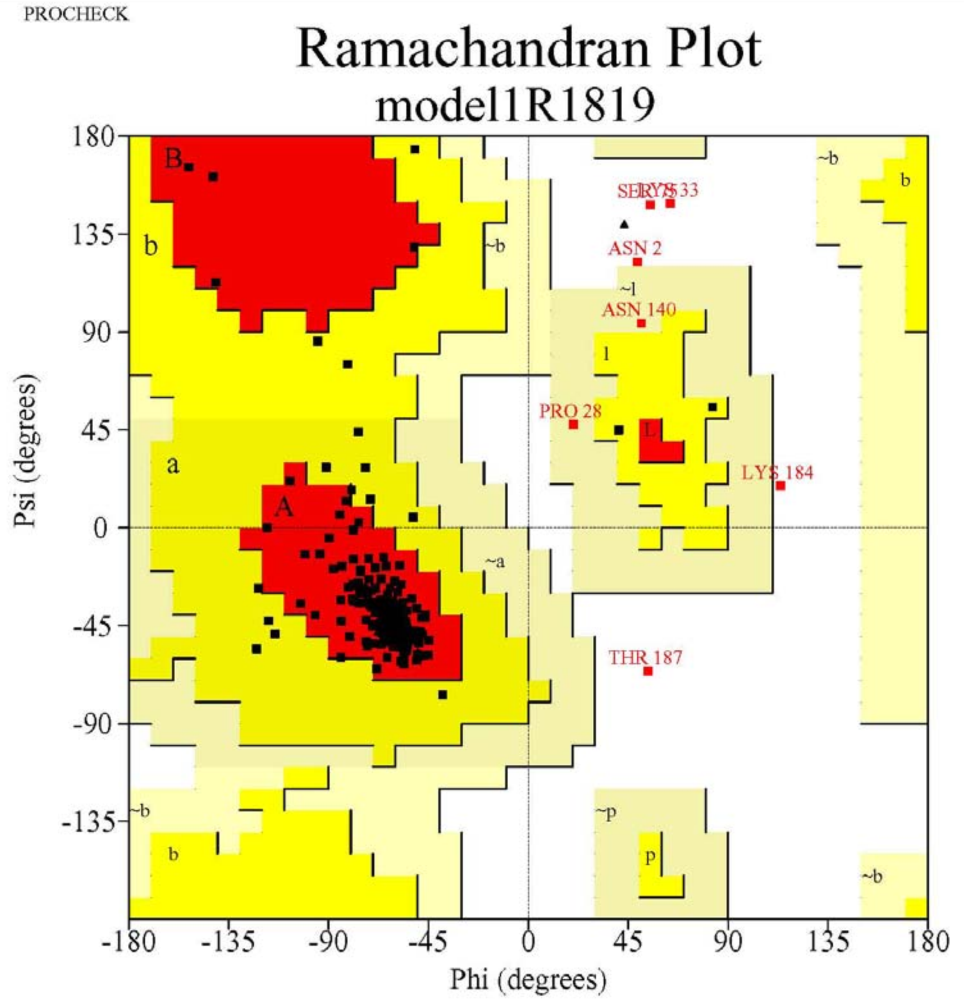

Plot statistics

Residues in most favoured regions [A,B,L]	175	90.2%
Residues in additional allowed regions [a,b,l,p]	13	6.7%
Residues in generously allowed regions [~a,~b,~l,~p]	1	.5%
Residues in disallowed regions	5	2.6%
	----	------
Number of non-glycine and non-proline residues	194	100.0%
Number of end-residues (excl. Gly and Pro)	2	
Number of glycine residues (shown as triangles)	8	
Number of proline residues	14	

Total number of residues	218	

Based on an analysis of 118 structures of resolution of at least 2.0 Angstroms and R-factor no greater than 20%, a good quality model would be expected to have over 90% in the most favoured regions.

model1R1819_01.ps

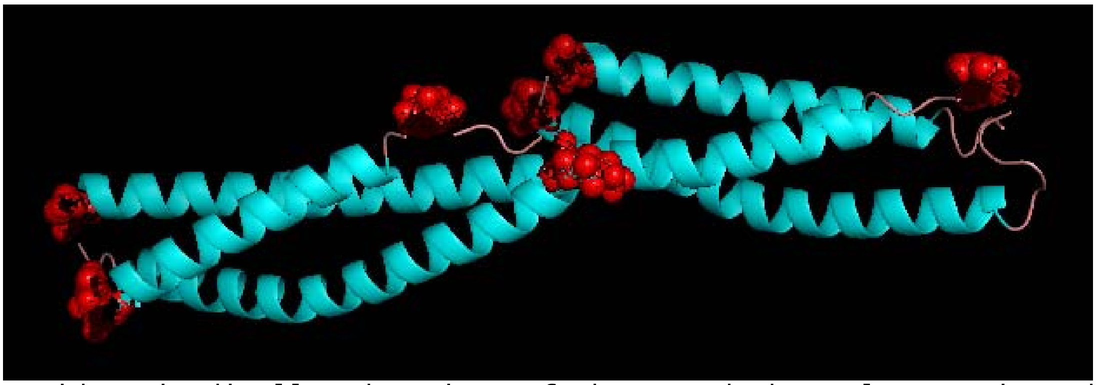

Residues in disallowed regions of the Ramachadran plot are in red

Fig S2

R20-21 model

VPALADFNRAWTELTDWLSLLDQVIKSQRVMVGDLEDINEMIIKQKATMQDLEQRRPQLEEL
ITAAQNLKNKTSNQEARTIITDRIERIQNQWDEVQEHLQNRRQQLNEM
LKDSTQWLEAKEEAEQVLGQARAKLESWKEGPYTVDAIQKKITETKQLAKDLRQWQTNVD
VANDLALKLLRDYSADDTRKVHMITENINASWRSIHKRVSEREAALEET

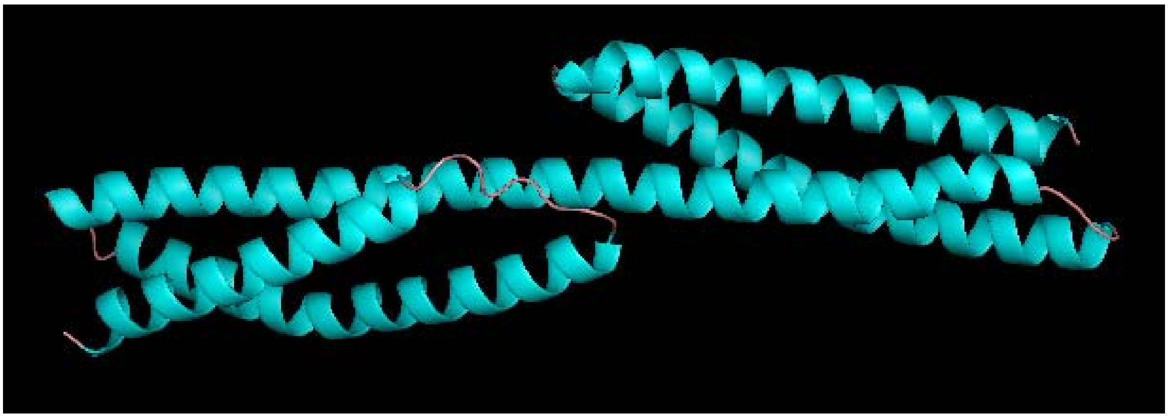

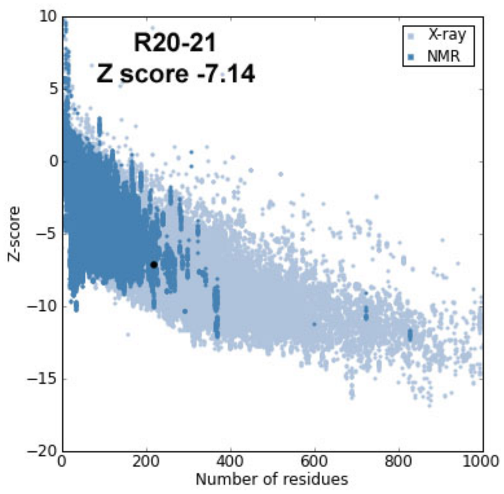

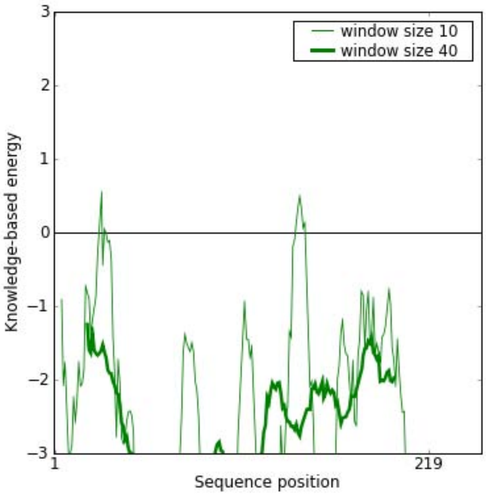

Verify3D structure evaluation

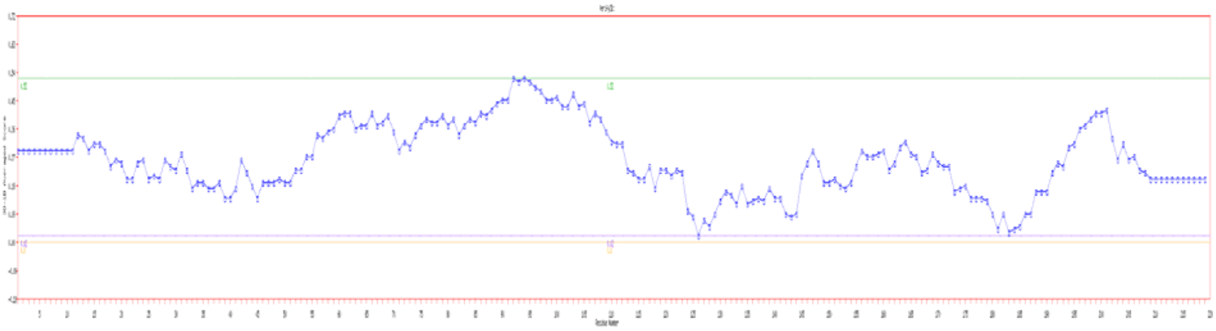

Fig S2

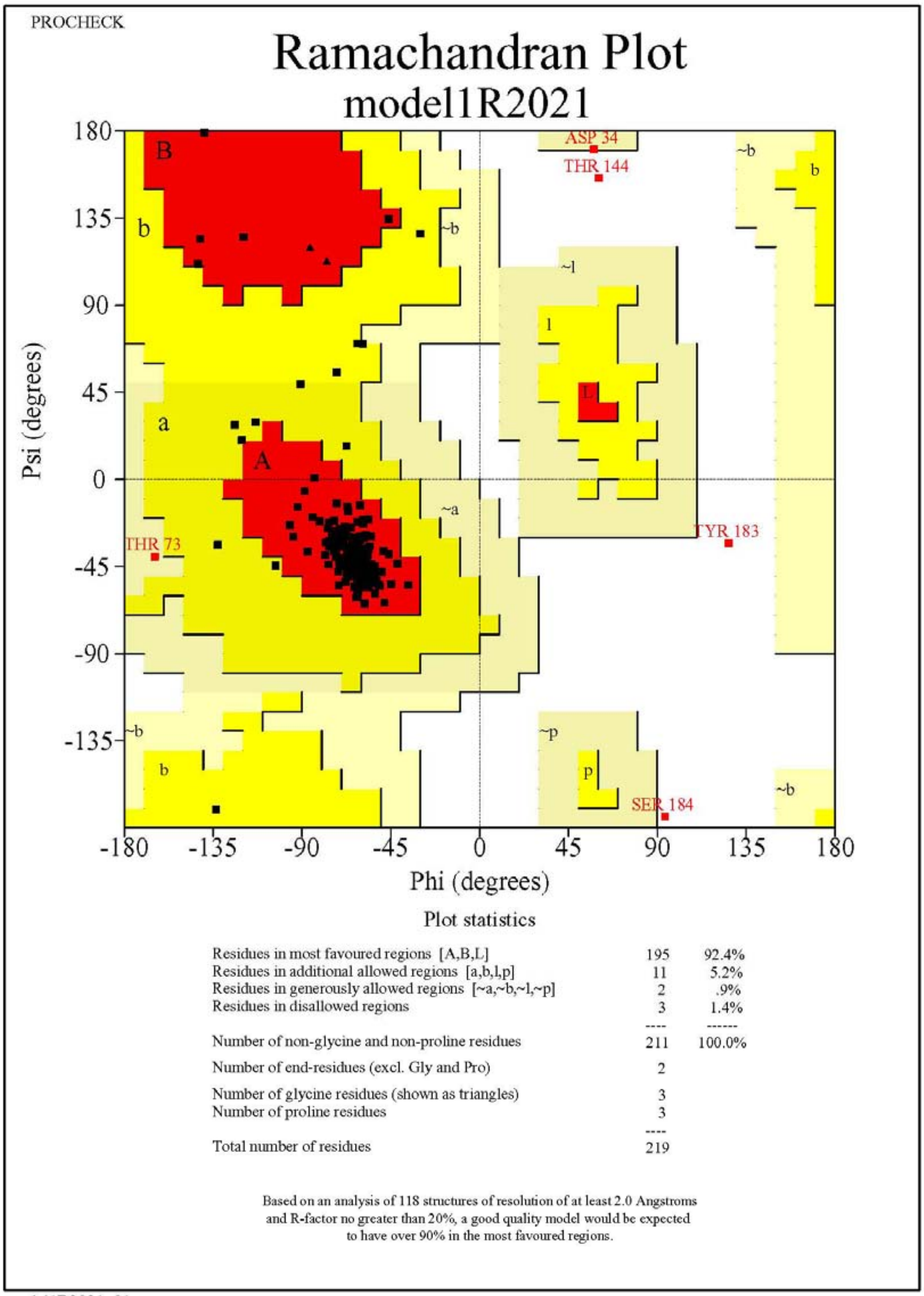

model1R2021_01.ps

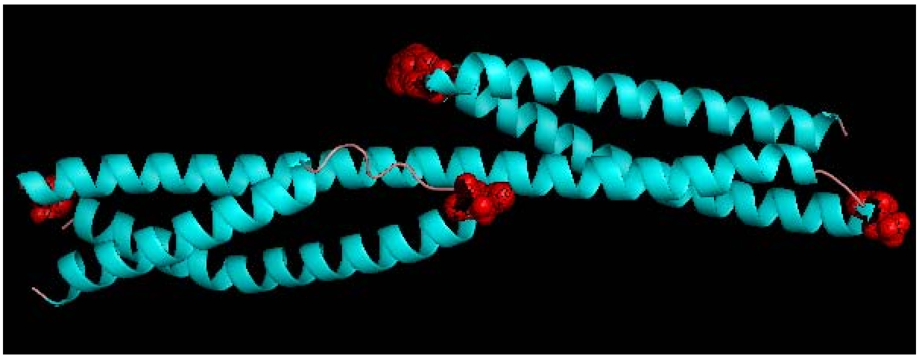

Residues in disallowed regions of the Ramachandran plot are in red

Fig S2

R21-22 model

LKDSTQWLEAKEEAEQVLGQARAKLESWKEGPYTVDAIQKKITETKQLAKDLRQWQTNVDVAN
DLALKLLRDYSADDTRKVHMITENINASWRSIHKRVSEREAALEET
HRLLQQFPLDLEKFLAWLTEAETTANVLQDATRKERELEDSKGVKELMKQWQDLQGEIEAHTDV
YHNLDENSQKILRSLEGSDDAVLLQRRLDNMNFKWSLLRKKSLNIRSHLEAS

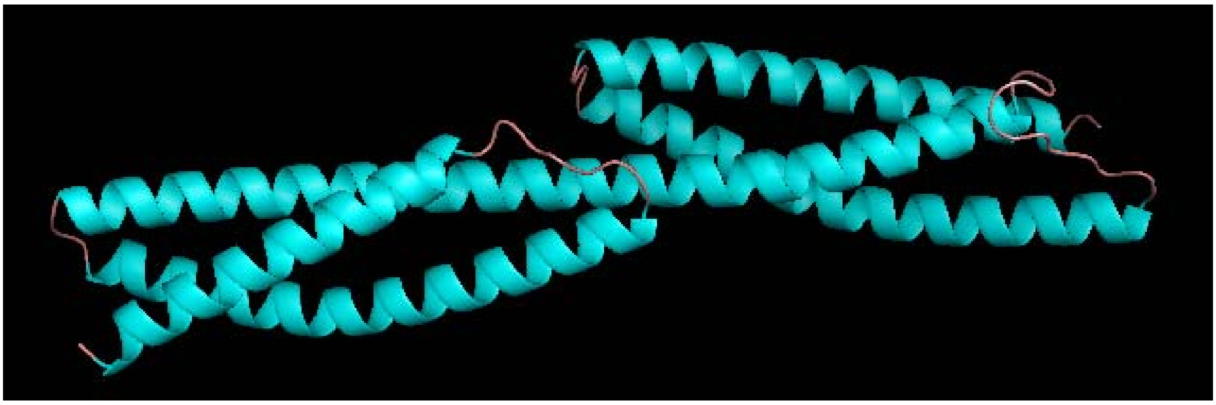

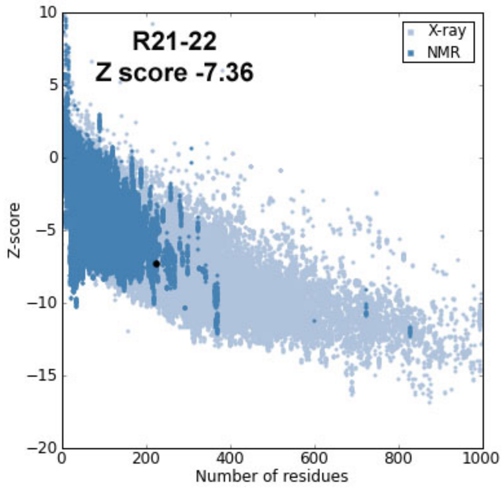

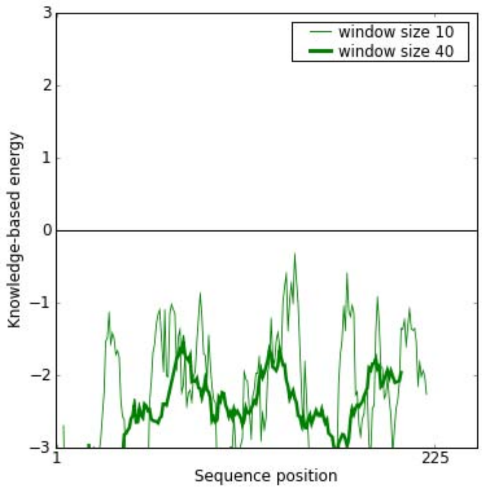

Verify3D structure evaluation

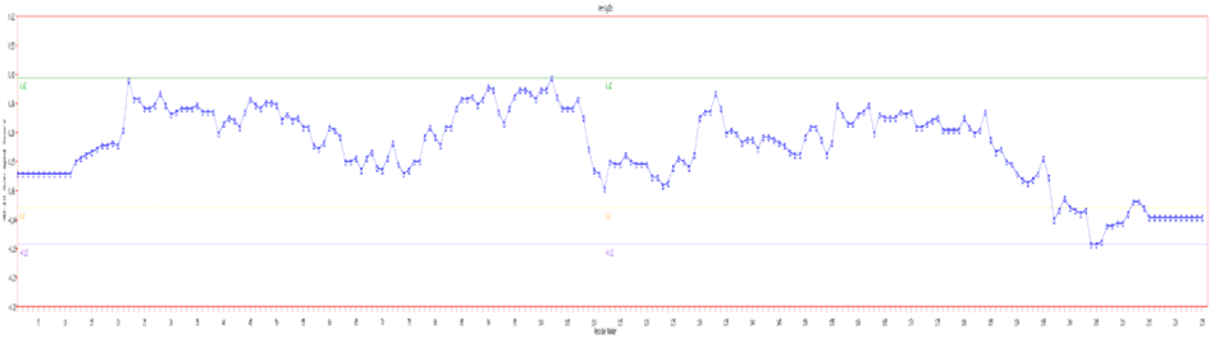

PROCHECK

Ramachandran Plot

model1R2122

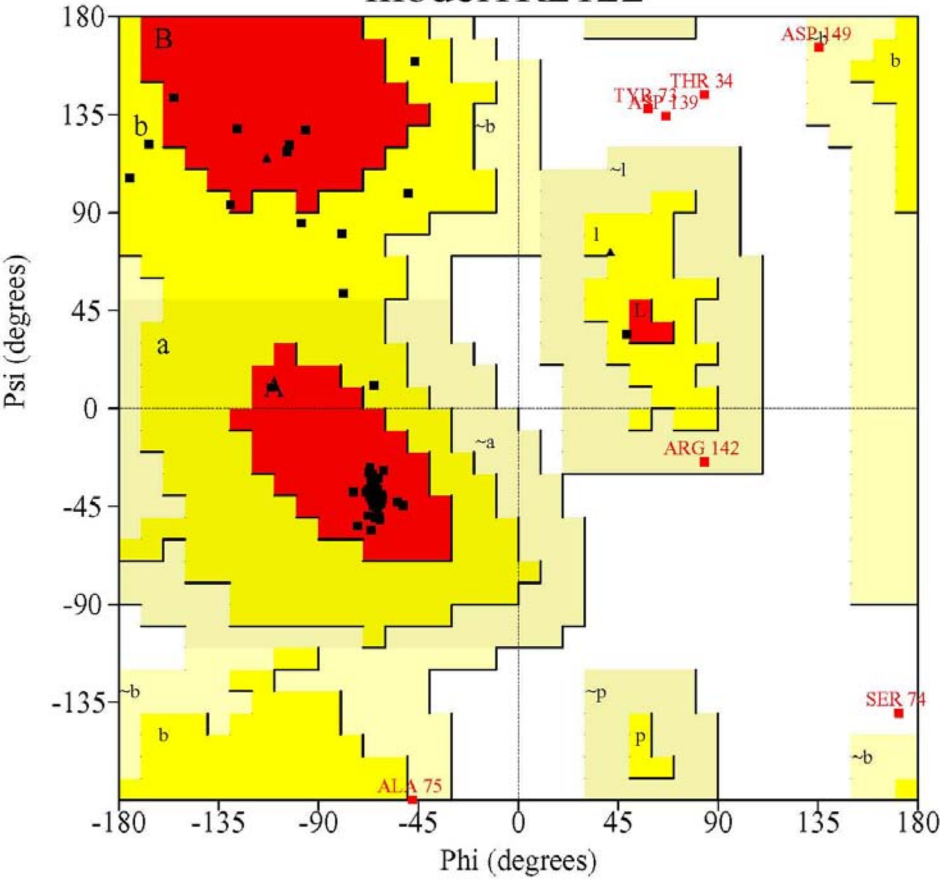

Plot statistics

Residues in most favoured regions [A,B,L]	201	93.1%
Residues in additional allowed regions [a,b,l,p]	8	3.7%
Residues in generously allowed regions [~a,~b,~l,~p]	3	1.4%
Residues in disallowed regions	4	1.9%
	----	------
Number of non-glycine and non-proline residues	216	100.0%
Number of end-residues (excl. Gly and Pro)	2	
Number of glycine residues (shown as triangles)	5	
Number of proline residues	2	

Total number of residues	225	

Based on an analysis of 118 structures of resolution of at least 2.0 Angstroms and R-factor no greater than 20%, a good quality model would be expected to have over 90% in the most favoured regions.

model1R2122_01.ps

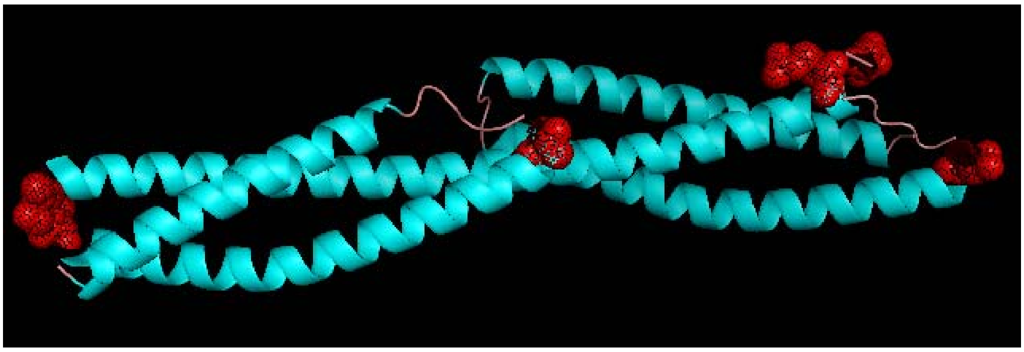

Residues in disallowed regions of the Ramachandran plot are in red

R22-23 model

HRLLQQFPLDLEKFLAWLTEAETTANVLQDATRKERLLEDSKGVKELMKQWQDLQGEIEAH
TDVYHNLDENSQKILRSLEGSDDAVLLQRRLDNMNFKWSELRKKSLNIRSHLEAS
SDQWKRLHLSLQELLVWLQLKDDELSRQAPIGGDFPAVQKQNDVHRAFKRELKTKEPVIMST
LETVRIFLTEQPLEGLEKLYQEPRELPPEERAQNVTRLLRKQAEEVNTEWEKLNLHSADWQR
KIDET

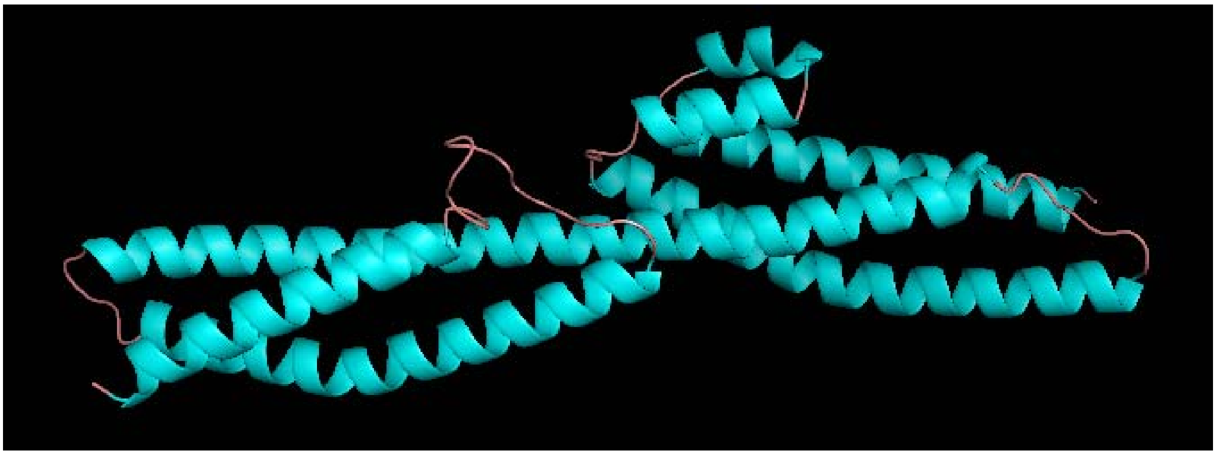

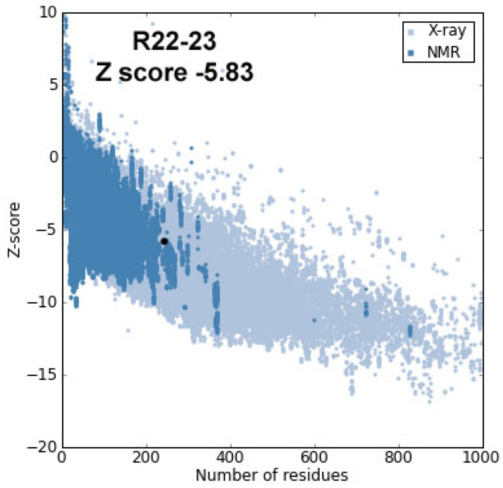

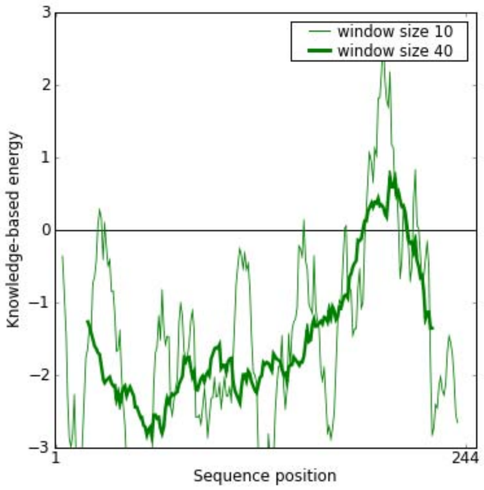

Verify3D structure evaluation

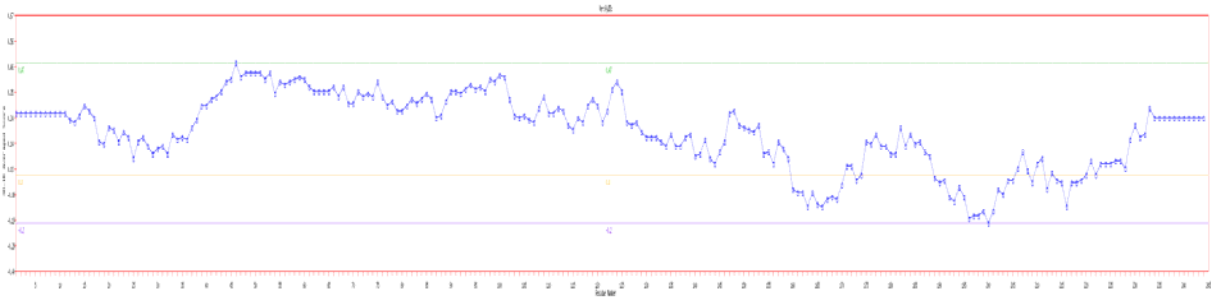

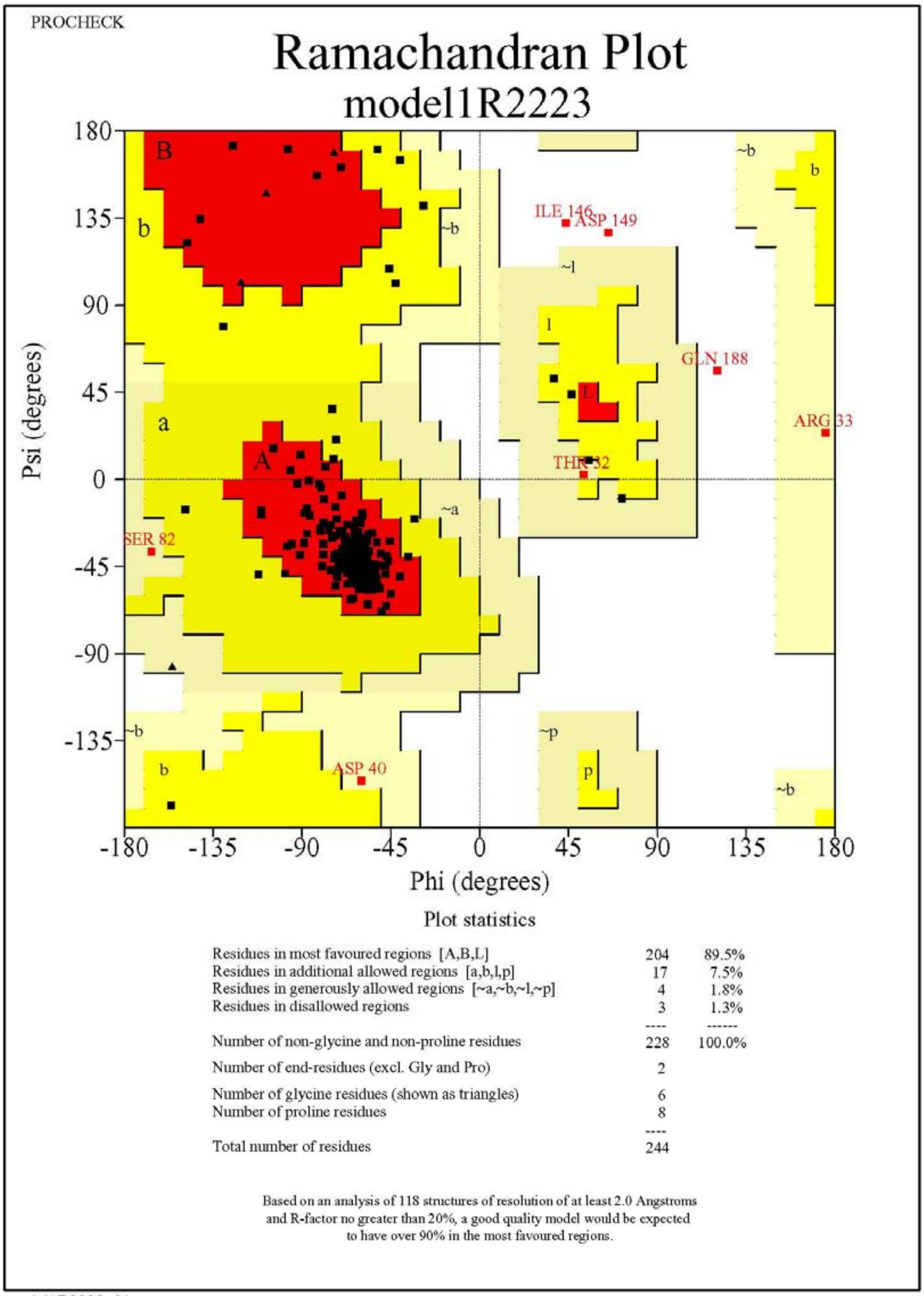

model1R2223_01.ps

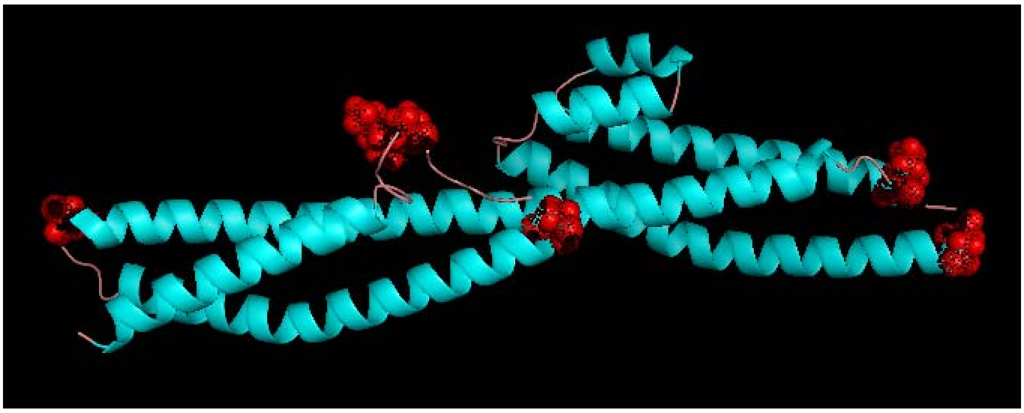

Residues in disallowed regions of the Ramachandran plot are in red

R23-24 model

SDQWKRLHLSLQELLVWLQLKDDELSRQAPIGGDFPAVQKQNDVHRAFKRELKTKEPVIM
STLETVRIFLTEQPLEGLEKLYQEPRELPPEERAQNVTRLLRKQAEEVNTEWEKLNLHSA
DWQRKIDETLERLQELQEATDELDLKLRQAEVIKGSWQPVGDLLIDSLQDHLEKVKALRG
EIAPLKENVSHVNDLARQLTTLGIQLSPYNLSTLEDLNTRWKLLQVAVEDRVRQLHE

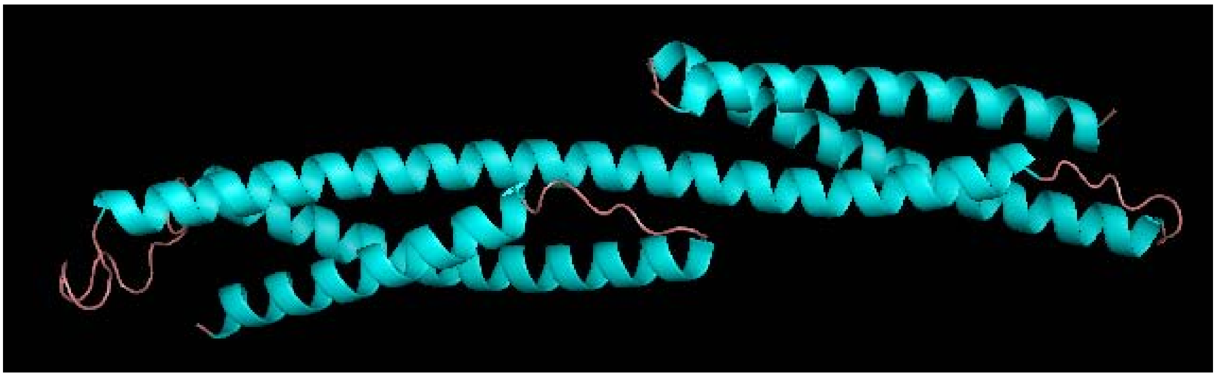

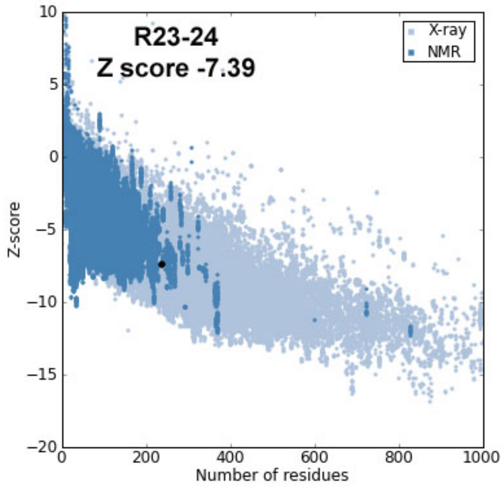

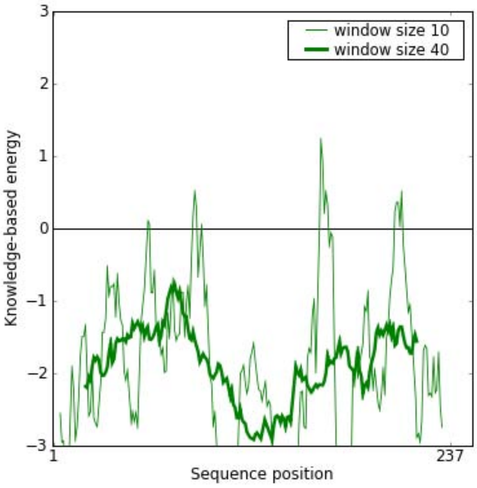

Verify3D structure evaluation

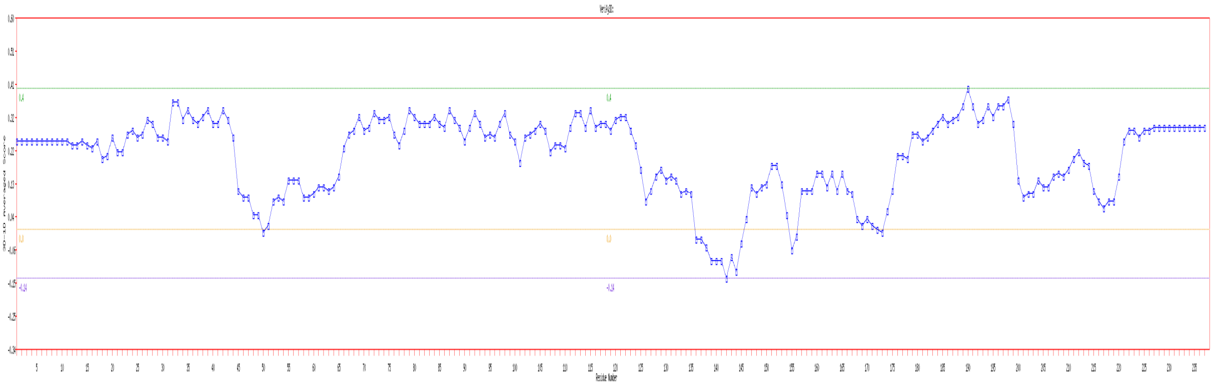

Fig S2

PROCHECK

Ramachandran Plot

model1R2324

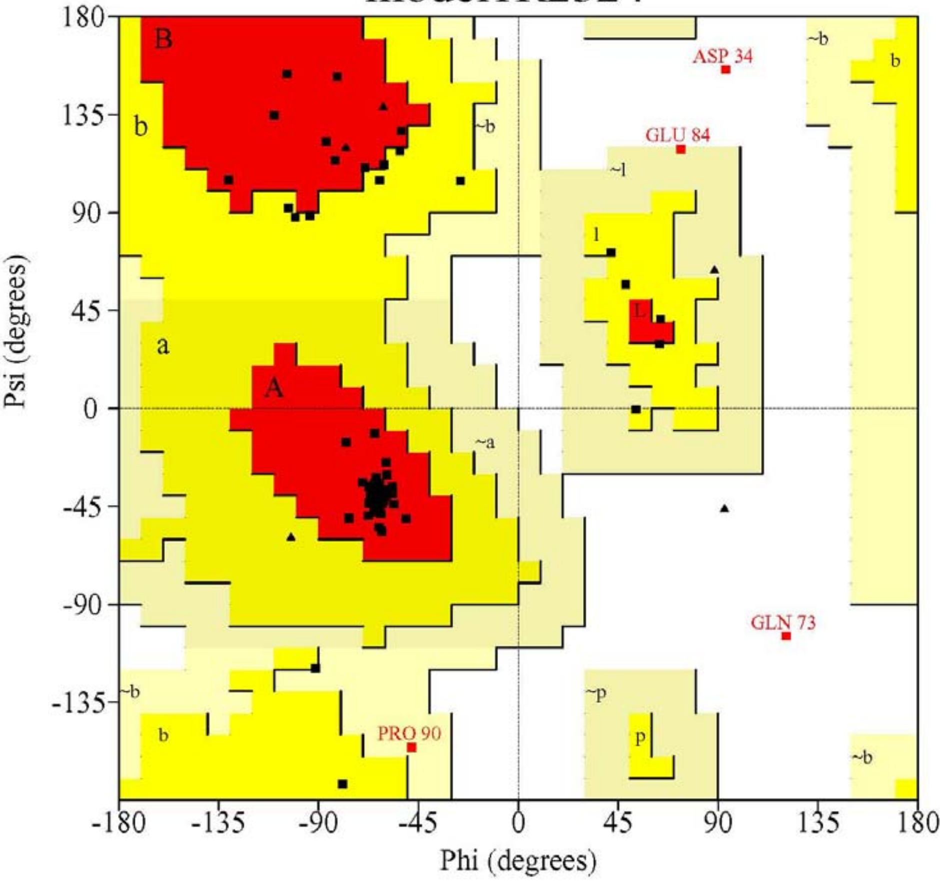

Plot statistics

Residues in most favoured regions [A,B,L]	203	93.1%
Residues in additional allowed regions [a,b,l,p]	12	5.5%
Residues in generously allowed regions [~a,~b,~l,~p]	1	.5%
Residues in disallowed regions	2	.9%
	----	------
Number of non-glycine and non-proline residues	218	100.0%
Number of end-residues (excl. Gly and Pro)	2	
Number of glycine residues (shown as triangles)	7	
Number of proline residues	10	

Total number of residues	237	

Based on an analysis of 118 structures of resolution of at least 2.0 Angstroms and R-factor no greater than 20%, a good quality model would be expected to have over 90% in the most favoured regions.

model1R2324_01.ps

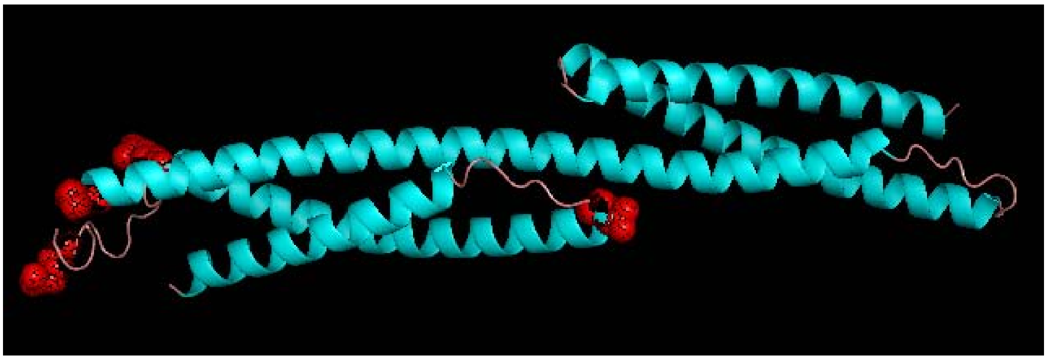

Residues in the disallowed regions of the Ramachandran plot are in red
